# A randomized, multicentre, open-label phase II proof-of-concept trial investigating the clinical efficacy and safety of the addition of convalescent plasma to the standard of care in patients hospitalized with COVID-19: the Donated Antibodies Working against nCoV (DAWn-Plasma) trial

**DOI:** 10.1186/s13063-020-04876-0

**Published:** 2020-11-27

**Authors:** Timothy Devos, Tatjana Geukens, Alexander Schauvlieghe, Kevin K. Ariën, Cyril Barbezange, Myriam Cleeren, Veerle Compernolle, Nicolas Dauby, Daniël Desmecht, David Grimaldi, Bart N. Lambrecht, Anne Luyten, Piet Maes, Michel Moutschen, Marta Romano, Lucie Seyler, Michel Toungouz Nevessignsky, Katleen Vandenberghe, Johan van Griensven, Geert Verbeke, Erika Vlieghe, Jean Cyr Yombi, Laurens Liesenborghs, Peter Verhamme, Geert Meyfroidt

**Affiliations:** 1 University Hospitals Leuven (UZ Leuven), Leuven, Belgium; 2 Catholic University of Leuven (KU Leuven), Leuven, Belgium; 3 Universitair Ziekenhuis Gent, Ghent, Belgium; 4 Instituut voor Tropische Geneeskunde, Antwerp, Belgium; 5 Sciensano, Elsene, Belgium; 6 Rode Kruis Vlaanderen, Mechelen, Belgium; 7 Universite Libre de Bruxelles Institut d’Immunologie Medicale, Bruxelles, Belgium; 8 Universite de Liege, Liege, Belgium; 9 Universite Libre de Bruxelles, Bruxelles, Belgium; 10 Leuven Coordinating Centre, Leuven, Belgium; 11 Katholieke Universiteit Leuven Rega Institute for Medical Research, Leuven, Belgium; 12 Universitair Ziekenhuis Brussel, Bruxelles, Belgium; 13 Croix Rouge de Belgique, Bruxelles, Belgium; 14 Interuniversity Institute for Biostatistics and statistical Bioinformatics, Leuven, Belgium; 15 Universitair Ziekenhuis Antwerpen, Antwerpen, Belgium; 16 Cliniques Universitaires Saint-Luc, Sint-Lambrechts-Woluwe, Belgium

**Keywords:** SARS-CoV-2, COVID-19, Convalescent plasma, Antibodies, Immunity

## Abstract

**Background:**

The COVID-19 pandemic has imposed an enormous burden on health care systems around the world. In the past, the administration of convalescent plasma of patients having recovered from SARS and severe influenza to patients actively having the disease showed promising effects on mortality and appeared safe. Whether or not this also holds true for the novel SARS-CoV-2 virus is currently unknown.

**Methods:**

DAWn-Plasma is a multicentre nation-wide, randomized, open-label, phase II proof-of-concept clinical trial, evaluating the clinical efficacy and safety of the addition of convalescent plasma to the standard of care in patients hospitalized with COVID-19 in Belgium. Patients hospitalized with a confirmed diagnosis of COVID-19 are eligible when they are symptomatic (i.e. clinical or radiological signs) and have been diagnosed with COVID-19 in the 72 h before study inclusion through a PCR (nasal/nasopharyngeal swab or bronchoalveolar lavage) or a chest-CT scan showing features compatible with COVID-19 in the absence of an alternative diagnosis. Patients are randomized in a 2:1 ratio to either standard of care and convalescent plasma (active treatment group) or standard of care only. The active treatment group receives 2 units of 200 to 250 mL of convalescent plasma within 12 h after randomization, with a second administration of 2 units 24 to 36 h after ending the first administration. The trial aims to include 483 patients and will recruit from 25 centres across Belgium. The primary endpoint is the proportion of patients that require mechanical ventilation or have died at day 15. The main secondary endpoints are clinical status on day 15 and day 30 after randomization, as defined by the WHO Progression 10-point ordinal scale, and safety of the administration of convalescent plasma.

**Discussion:**

This trial will either provide support or discourage the use of convalescent plasma as an early intervention for the treatment of hospitalized patients with COVID-19 infection.

**Trial registration:**

ClinicalTrials.govNCT04429854. Registered on 12 June 2020 - Retrospectively registered.

**Supplementary information:**

The online version contains supplementary material available at 10.1186/s13063-020-04876-0.

## Administrative information

**Table:** 

Title {1}	A randomized, multicentre, open-label phase II proof-of-concept trial investigating the clinical efficacy and safety of the addition of convalescent plasma to the standard of care in patients hospitalized with COVID-19: the Donated Antibodies Working against nCoV (DAWn-Plasma) trial.
Trial registration {2a and 2b}.	Clinicaltrials.gov, Identifier: NCT04429854. Registered 12 June 2020 - Retrospectively registered, https://clinicaltrials.gov/ct2/show/NCT04429854.
Protocol version {3}	Version 2.3
Funding {4}	Conduct of the study: The Belgian Health Care Knowledge Centre (KCE). Set-up and maintenance of the trial’s website: Life Sciences Research Partners (LSRP).
Author details {5a}	Timothy Devos, MD PhD, Department of Haematology, UZ Leuven and Department of Microbiology and Immunology, Laboratory of Molecular Immunology (Rega Institute), KU Leuven, Leuven, Belgium. Tatjana Geukens, MD, Laboratory for Translational Breast Cancer Research, KU Leuven, Leuven, Belgium. Alexander Schauwvlieghe, MD, Department of Haematology, UZ Leuven, Leuven, Belgium. Kevin K. Ariën, PhD, Virology Unit, Institute of Tropical Medicine Antwerp, Antwerp, and Department of Biomedical Sciences, Faculty of Pharmaceutical, Biomedical and Veterinary Sciences, University of Antwerp, Antwerp, Belgium. Cyril Barbezange, MD PhD, Infectious Diseases in Humans Scientific Directorate, Sciensano, Belgium. Myriam Cleeren, Department of Haematology, UZ Leuven, Leuven, Belgium. Veerle Compernolle, MD PhD, Rode Kruis-Vlaanderen, Mechelen, Belgium. Nicolas Dauby, MD PhD, Department of Infectious diseases, UMC Saint-Pierre, Brussels, Belgium and Institute for Medical Immunology, Université Libre de Bruxelles (ULB), Brussels, Belgium. Daniël Desmecht, PhD, Department of Morphology and Pathology Université de Liège, Liège, Belgium. David Grimaldi, MD PhD, Department of Intensive Care Medicine, CUB-Erasme, Université Libre de Bruxelles (ULB), Brussels, Belgium. Bart N. Lambrecht, MD PhD, Department of Respiratory Diseases, UZ Gent, Gent, Belgium. Anne Luyten, Leuven Coordinating Centre (LCC), KU Leuven, Leuven, Belgium. Piet Maes, PhD, Department of Microbiology, Immunology and Transplantation, Rega Institute, KU Leuven, Leuven, Belgium. Michel Moutschen, MD PhD, Department of Infectious Diseases and General Internal Medicine, Université de Liège, Liège, Belgium. Marta Romano, PhD, Infectious Diseases in Humans Scientific Directorate, Sciensano, Belgium. Lucie Seyler, MD PhD, Department of Infectious Diseases, UZ Brussel, Brussels, Belgium. Michel Toungouz Nevessignsky, MD PhD, Croix Rouge de Belgique, Suarlée, Belgium. Katleen Vandenberghe, PhD, Leuven Coordinating Centre (LCC), KU Leuven, Leuven, Belgium. Johan van Griensven, MD PhD, Institute of Tropical Medicine, Antwerp, Belgium. Geert Verbeke, MD PhD, Department of Public Health and Primary Care, KU Leuven, Leuven, Belgium and Interuniversity Institute for Biostatistics and statistical Bioinformatics (I-BioStat), KU Leuven, Leuven, and Hasselt University (UHasselt), Hasselt, Belgium. Erika Vlieghe, MD PhD, Department of Infectious Diseases, General Internal Medicine and Tropical Medicine, Antwerp University Hospital, Antwerp, Belgium. Jean Cyr Yombi, MD PhD, Department of Internal Medicine and Infectious diseases, Cliniques Universitaires Saint-Luc, UCLouvain, Brussels, Belgium. Laurens Liesenborghs, MD PhD, Laboratory of Virology and Chemotherapy (Rega Institute), Leuven, Belgium. Peter Verhamme, MD PhD, Department of Cardiovascular Diseases, UZ Leuven, Leuven, Belgium. Geert Meyfroidt, MD PhD, Department of Intensive Care Medicine, UZ Leuven, Leuven, Belgium.
Name and contact information for the trial sponsor {5b}	UZ Leuven Prof. Dr. Geert Meyfroidt geert.meyfroidt@uzleuven.be
Role of sponsor {5c}	The trial was designed and the protocol was written by the sponsor and approved by the KCE. The sponsor was responsible for submission to regulatory authorities, set up of the trial and selection of the investigators, and will be responsible for monitoring of the study, compliance with safety regulations, labelling, reporting and record-keeping.

## Introduction

### Background and rationale {6a}

In December 2019, the Wuhan Municipal Health Committee identified an outbreak of viral pneumonia cases of unknown cause. Coronavirus RNA was quickly identified in some of these patients. This novel coronavirus has been named SARS-CoV-2, and the disease was caused by this virus COVID-19. Currently, there are no approved therapeutic agents available for coronaviruses [1].

Plasma collected from patients who have recovered from a SARS-CoV-2 infection can contain antiviral antibodies detectable through serological testing [2]. It is unclear to what extent these antibodies provide anti-viral protection, but at least a subset of patients presents with high titres of neutralizing antibodies, or with specific potent antibodies irrespective of the titre [3]. Passing on this plasma to patients newly diagnosed with SARS-CoV-2 infection might help them to clear the infection more rapidly, as it could provide them with passive polyclonal antibodies and thus reduce the antiviral load. The treatment effect of plasma has already been shown in some viral infections but has not been studied in patients with SARS-CoV-2 infection. A recent meta-analysis has shown that convalescent plasma reduces mortality compared to standard of care in patients with SARS and severe influenza infections [4]. Importantly, these studies were heterogeneous on the timing of plasma administration and the threshold on anti-virus antibodies in convalescent donors. It is particularly successful when administered early after symptom onset. On the other hand, convalescent plasma has proven not to be effective against other viral infections (e.g. Ebola). It additionally carries a risk of antibody-dependent enhancement (ADE), which could result in worsening of the acute respiratory distress syndrome (ARDS), and in Argentine haemorrhagic fever convalescent plasma administration significantly reduced mortality rates, but was found to be associated with a late neurological syndrome [5].

Knowledge regarding the immune response to SARS-CoV-2 infection is rather limited at this point. It seems that seroconversion occurs within 2 weeks after infection. Recently, a case series showed that convalescent plasma possibly had a contributory effect on the clinical improvement of 5 critically ill patients with SARS-CoV-2 and acute respiratory distress syndrome [6]. Moreover, evaluation of 5000 patients having received COVID-19 convalescent plasma in the USA showed the rate of immediate serious adverse events related to the transfusion to be very low [7], noting that in an open-label setting, a control group was missing for comparison. Therefore, more data from controlled trials are urgently needed to assess the possible role of convalescent plasma during the COVID-19 pandemic.

This study will therefore evaluate the effect of passive immunotherapy with convalescent plasma in the treatment of patients with a newly diagnosed COVID-19 disease requiring hospitalization in a randomized controlled setting. The hypothesis is that early administration of plasma will help to counter the clinical deterioration by providing immediate (passive) immunity when pathology is still mainly driven by viral replication and hence will prevent the need for mechanical ventilation or death in the first 15 days after randomization. It is of utmost importance to identify treatment strategies that reduce the severity of the disease and thereby intensive care unit (ICU) demand during this pandemic with a high health care burden. Additionally, as strategies of prolonged distancing are likely to have a negative social and economic impact, there is a need for efficient and safe treatment perspectives for COVID-19 patients [8]. The study complies with the recommendations for outcomes as outlined by the World Health Organization (WHO) master template protocol [9, 10].

### Objectives {7}

The overall objective of the DAWn-plasma study is to evaluate the clinical efficacy and safety of the addition of convalescent plasma to the standard of care in patients hospitalized with COVID-19.

The primary endpoint of the study is the number of patients alive without mechanical ventilation at day 15 after hospitalization, with the null hypothesis being that convalescent plasma will not be effective to prevent the need for mechanical ventilation or death.

Secondary endpoints are as follows:

- Clinical status of subject at day 15 and day 30 (on a 10-point “WHO progression” ordinal scale):
  0. Uninfected, no viral RNA detected
  1 Ambulatory, asymptomatic, viral RNA detected
  2 Ambulatory, symptomatic, ndependent
  3 Ambulatory, symptomatic, assistance needed
  4 Hospitalized, mild disease, no oxygen therapy needed
  5 Hospitalized, mild disease, oxygen by mask of nasal prongs
  6 Hospitalized, severe disease, oxygen by NIV or high flow
  7 Hospitalized, severe disease, intubation and mechanical ventilation (pO_2_/FiO_2_ > =150 OR SpO_2_/FiO_2_ ≥ 200)
  8 Hospitalized, severe disease, mechanical ventilation (pO_2_/FiO_2_ < 150 OR SpO_2_/FiO_2_ < 200) OR vasopressors (norepinephrine > 0.3 μg/kg/min)
  9 Hospitalized, severe disease, mechanical ventilation pO_2_/FiO_2_ < 150 AND vasopressors (norepinephrine > 0.3 μg/kg/min), OR dialysis OR ECMO
  10 Death, dead
- Cumulative clinical status of subject up to day 15 (on a 10-point ordinal scale): the sum of the daily clinical status score for days 1 up to 15
- Proportion of patients having been on mechanical ventilation or are dead at 30 days and 90 days after randomization
- Status on an ordinal scale assessed daily while hospitalized and on days 15 and 30
- Time to clinical improvement (number of days from hospitalization to first 2-point improvement from highest previously recorded clinical state on the 10-point ordinal scale)
- Duration of hospitalization
- Duration of supplemental oxygen treatment
- Duration of mechanical ventilation.
- Need for and duration of intensive care stay
- Need for and duration of extracorporeal membrane oxygenation (ECMO)
- Date and cause of death (if applicable).
- Adverse events graded as severe (SAEs)
- Venous thromboembolism: deep vein thrombosis or pulmonary embolism
- Transfusion-related side effects such as transfusion-related acute lung injury, serious allergic transfusion reactions and transfusion-associated circulatory overload.
- Correlation between clinical outcome and titre of anti-SARS-CoV-2 neutralizing antibodies in transfused plasma units
- Safety of convalescent plasma therapy
- Effect of plasma therapy on quality of life 30 days after randomization
- Vital signs, being daily highest temperature measured during hospitalization with a maximum of 14 days after hospitalization and highest flow of oxygen given (in L/min) daily during hospitalization with a maximum of 14 days after hospitalization

### Trial design {8}

This DAWn-Plasma study is a randomized, open-label clinical trial to evaluate the safety and efficacy of the addition of convalescent plasma to standard of care in hospitalized adult patients diagnosed with COVID-19. The outcomes of the study protocol are in part based on the draft master protocol of the WHO for trials that evaluate the safety and efficacy of investigational therapeutics for the treatment of COVID-19 in hospitalized patients.

This study is a phase II proof-of-concept multicentre trial. It will compare standard of care vs. standard of care with convalescent plasma. Since the beginning of the COVID-19 pandemic, the standard of care (SOC) has been mostly supportive, in view of the lack of evidence for specific therapies for this novel disease. However, the standard of care may change during the course of the study as (inter) national guidelines might be subject to change as results from randomized controlled trials (RCTs) on anti-viral, anti-inflammatory or anti-coagulation therapy become available. The clinical outcomes of this study have been chosen based on the outcomes of the WHO master template for clinical studies to allow pooling of the data with other ongoing studies.

The DAWn-Plasma will randomize with a 2:1 allocation to SOC combined with convalescent plasma versus SOC.

## Methods: Participants, interventions and outcomes

### Study setting {9}

DAWn-Plasma is a multicentre study and will recruit patients in 25 Belgian hospitals, both academic and non-academic. The contributing institutions are mentioned on clinicaltrials.gov (NCT NCT04429854).

### Eligibility criteria {10}

Participants eligible for inclusion in this trial must meet all of the following criteria:

- Subject (≥ 18 years old) or legally authorized representative provides informed consent prior to initiation of any study procedures. When signed informed consent is not possible (e.g. due to restrictions to prevent viral transmission), verbal informed consent in the presence of an independent witness will be obtained and documented in the medical files. Signed informed consent will be obtained as soon as the safety concerns are mitigated
- Subject (or legally authorized representative) understands and agrees to comply with planned study procedures
- Male or non-pregnant female adult ≥ 18 years of age at time of enrolment
- Patient should be hospitalized
- Has a confirmed diagnosis of SARS-CoV-2 infection, defined as *either*:
  a Laboratory-confirmed SARS-CoV-2 infection as determined by PCR, or other commercial or public health assay in any specimen as diagnosed within 72 h prior to randomization, or
  b The combination of upper or lower respiratory infection symptoms (fever, cough, dyspnoea, desaturation) and typical findings on chest CT scan and absence of other plausible diagnoses
- Illness of any duration, and at least one of the following:
  a Radiographic infiltrates by imaging (chest X-ray, CT scan or other), or
  b Abnormalities on clinical assessment (evidence of rales/crackles on exam) and oxygen saturation (SpO_2_) ≤ 94% on room air, or
  c Requiring supplemental oxygen.
- ABO D typing of the patient should be done at least once and the result should be known at time of inclusion.

Participants eligible for this trial must not meet any of the following criteria:

- Receiving invasive (any mode where a patient has been intubated endotracheally or via tracheostomy) or non-invasive mechanical ventilation before or upon randomization.
- Pregnancy or breastfeeding.
- Any medical condition which would impose an unacceptable safety hazard by participation to the study, as deemed by the investigator.
- Patients with a history of a documented grade 3 allergic reaction after the administration of fresh frozen plasma (i.e. systemic reaction with cardiovascular and/or respiratory involvement).
- Patients that have treatment restrictions that exclude mechanical ventilation and/or endotracheal intubation.

### Who will take informed consent? {26a}

Potential participants are screened on the emergency ward or upon arrival at the COVID-ward. Accordingly, an emergency physician or a supervising physician at the COVID-ward that is trained in the protocol will first assess the patient’s interest in the study. In case a patient is interested to participate, the investigators of the DAWn-study team again check the eligibility criteria and contact the patient to provide more information and obtain an informed consent.

The consent form includes a short and comprehensible summary of the rationale of the trial, the trial design and the study drug. This is followed by an elaborate form, where all study-related procedures, clinical data collection (for instance clinical scores and vital signs), biosample collection (for instance blood draws and nasal swabs) and the potential risks (potential adverse events) and benefits (potential positive effect of the intervention, contribution to knowledge production) from the study are explained. Data management and ethical approval are detailed, as well as the insurance policy. The investigator also verbally explains this consent form and is available for questions before the patient is asked to sign the consent form. A copy of the informed consent form is attached to this manuscript as Supplementary Material.

### Additional consent provisions for collection and use of participant data and biological specimens {26b}

In case no DAWn-investigator is on duty on the COVID-ward at the time of inclusion, obtaining a written consent within the time window of inclusion, but without wasting extra personal protective equipment is difficult. In order to avoid this, we were granted permission by the ethical committee to do the first contact between the DAWn-investigator and patient by telephone. The consent form is read and illustrated by the investigator on the phone, and the patient gives verbal consent. An independent witness is in the presence of the patient or the investigator to confirm that the patient has understood the study procedures and agrees with them. Data collection, randomization and, in case the patient is randomized to the active treatment group, administration of the plasma can then already start awaiting the signed version of the written informed consent form that will be sent to the investigator by the patient at a point in time when it is safe to do so.

## Interventions

### Explanation for the choice of comparators {6b}

The addition of convalescent plasma to standard of care will be compared to standard of care treatment for patients hospitalized with COVID-19. No active comparator drug is imposed by the trial.

### Intervention description {11a}

#### Screening/baseline

**Fig. 1:**
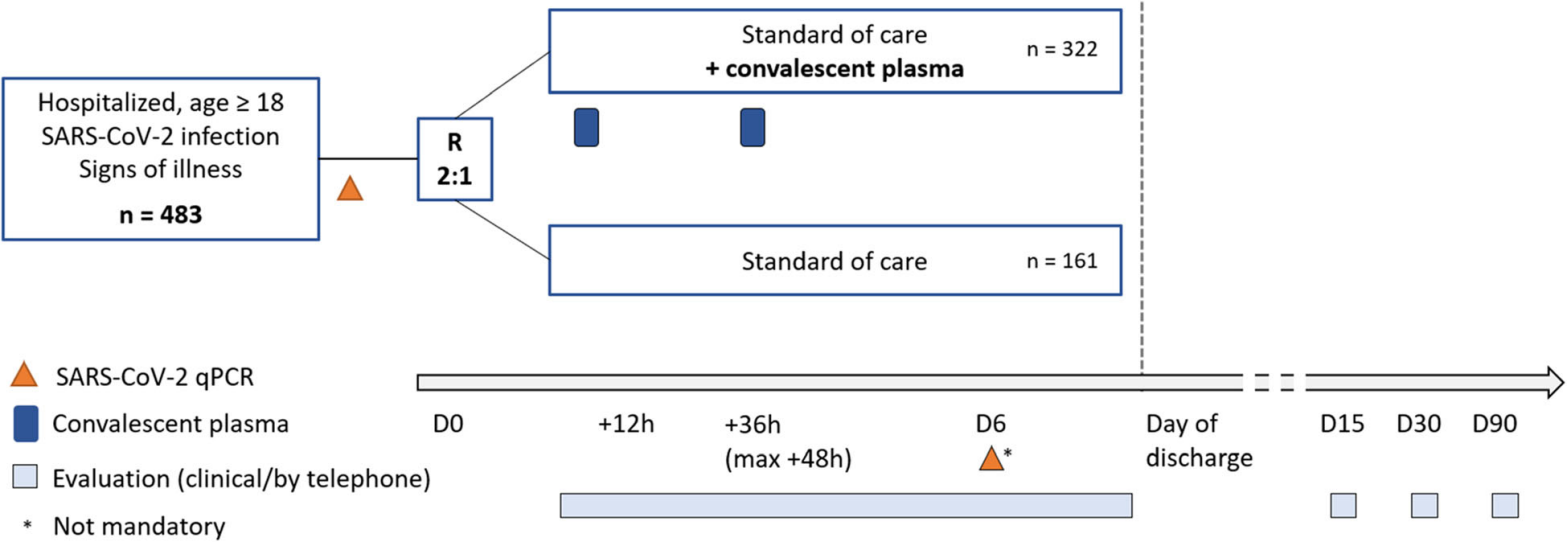
Overview of the study interventions

Demographic parameters will be obtained. Medical history will be obtained as part of routine clinical care. Concomitant medication will be reviewed using the electronic medical files. A first blood group determination should be done upon hospitalization of the patient to facilitate fast plasma administration following randomization. A nasopharyngeal swab for viral qPCR analysis is taken at baseline, on day 0 (+/− 2) (Fig. 1).

#### Randomization

To ensure the integrity of the trial, a randomization procedure through the software REDCap has been established, generated by the data management unit of the clinical trial centre Leuven. Patients from all centres will be randomized centrally, in a 2 (active treatment group) to 1 (SOC) ratio.

#### Administration of trial product

In case the patient is randomized to the active treatment group, convalescent plasma (2 units of 200 to 250 mL) will be administered within 12 h after randomization, with a second administration (2 units of 200 to 250 mL) 24 to 36 h after ending the first administration. Plasma should not be transfused at the usual speed of 30 to 45 min but should be administered at a maximal speed of 50 to 100 mL per hour, in order to reduce the risk of volume overload. Parameters will be monitored during the administration of plasma according to good clinical practice.

COVID-19 convalescent plasma (CCP) donors are recruited in a population of patients that were infected with COVID-19 and recovered. At least 28 days should have passed since full recovery and disappearance of the symptoms. Potential donors must at least fulfil national legal requirements for eligibility of donors to donate blood or plasma. The Blood Establishment qualify donations from donors with neutralizing antibody titres greater or equal to 1/320 as appropriate for this study.

#### Daily assessments until discharge

The following information will be retrieved daily until the day the patient is discharged from the hospital: (1) vital signs including SpO_2_, (2) clinical data for assessment of study outcomes and (3) adverse event evaluation. Serious adverse events and adverse events grade IV will be collected even when these are not part of the endpoints of the study.

#### Visit at day 15 (+/− 2), 30(+/− 3) and 90 (+/− 5)

In case the patient is no longer hospitalized on the days a clinical assessment is needed for evaluation of the primary and secondary endpoints, these visits can be replaced by telephone contacts with the patient enquiring about their status.

#### Additional sampling

The study includes two optional samples:

- At baseline: on day 0 (+/− 2): blood samples for the assessment of immunoparesis against SARS-CoV-2 and antibody clearance. This blood samples should be collected before the first infusion of convalescent plasma. Samples will be stored locally and shipped in batches to the central reference lab. The practical details of local storage and shipment can be found in the lab manual.
- On day 6 (+/− 2): blood samples for the assessment of immunoparesis against SARS-CoV-2 and antibody clearance. Samples will be stored locally and shipped in batches to the central reference lab. The practical details of local storage and shipment can be found in the lab manual.

The study includes a sample to be taken, when feasible:

- Viral qPCR (nasopharyngeal swab) at day 6 (+/− 2).

### Criteria for discontinuing or modifying allocated interventions {11b}

Participants may voluntarily discontinue trial treatment and/or prematurely end their participation in the trial for any reason at any time. In such case, the investigator must make a reasonable effort to contact the participant (e.g. via telephone, e-mail, letter) in order to document the primary reason for this decision.

The investigator may also decide at any time during the course of the trial, to temporarily interrupt or permanently discontinue the trial treatment if it is deemed that continuation would be detrimental to, or not in the best interest of the participant.

Similarly, the sponsor, Ethics Committee (EC) or authorized regulatory authority can decide to halt or prematurely terminate the trial when new information becomes available whereby the rights, safety and well-being of trial participants can no longer be assured, when the integrity of the trial has been compromised, or when the scientific value of the trial has become obsolete and/or unjustifiable.

Circumstances requiring premature treatment interruption or discontinuation of the trial, include but are not limited to: (1) safety concerns related to blood product or unacceptable intolerability (potentially life-threatening transfusion reaction during plasma infusion), (2) trial participation while in violation of the inclusion and/or exclusion criteria and (3) pregnancy or the intention of becoming pregnant. In any such case of early trial termination and/or treatment interruption/discontinuation, the investigator will continue to closely monitor the participant’s condition and ensure adequate medical care and follow-up. Additionally, these patients will continue to be followed for the primary outcome and their data will be included in intention-to-treat analyses.

For participants whose status is unclear because they fail to appear for trial visits without stating an intention to discontinue or withdraw, the investigator must make every effort to demonstrate “due diligence” by documenting in the source documents which steps have been taken to contact the participant to clarify their willingness and ability to continue their participation in the trial (e.g. dates of telephone calls, registered letters). A participant should not be considered lost to follow-up until due diligence has been completed.

### Strategies to improve adherence to interventions {11c}

For the administration of convalescent plasma, a specific standard operating procedure (SOP) was created. Detailed information on administration is provided within the hospital’s electronic system and is displayed when nurses administer the plasma. Permanent remote assistance is available as investigators are standby 24 h a day, 7 days a week.

### Relevant concomitant care permitted or prohibited during the trial {11d}

Patients will receive the standard of care treatment for COVID-19 as pointed out by national and international guidelines at the time of diagnosis. Any drugs or procedures required in that context are permitted. The administration of any investigational medicinal products (IMPs) other than plasma is not allowed. No other drugs or procedures are prohibited during this trial.

### Provisions for post-trial care {30}

As per European legislation, the sponsor has a full insurance that covers the costs of potential harms.

### Outcomes {12}

The primary outcome of the study is the number of patients alive without mechanical ventilation at day 15 after hospitalization. The primary objective of the study is to avoid further clinical decline by administering convalescent plasma to hospitalized patients early after symptom onset to provide immediate (passive) immunity. The hypothesis is that early administration of plasma will help to counter the clinical deterioration when pathology is mainly driven by viral replication and, hence, will prevent the need for mechanical ventilation or death in the first 15 days after randomization. The null hypothesis is that convalescent plasma will not be effective to prevent the need for mechanical ventilation or death.

Secondary outcomes are defined in the “Outcomes” section of this protocol (cfr. supra).

Exploratory outcomes are as follows:

- Qualitative and quantitative PCR for SARS-CoV-2 in (nasopharyngeal) swab on day 6 (when feasible)
- Comparing clinical efficacy of convalescent plasma in patients that already had anti-COVID19 antibodies before the administration of the plasma compared with patients that did not. These results will not be available real-time but are subject to a post hoc analysis

### Participant timeline {13}

Table 1 shows the participant timeline.

**Table 1:** Participant timeline

Day +/− window	Screen	Baseline	Daily until discharge	Within 12 h after randomization*	24–36 h after 1st administration*	6 +/− 2	15 +/− 2	30 +/− 3	Day 90+/− 5
	− 1 or 0	0							
**Assessments/procedures**									
**Eligibility**									
Informed consent	X								
Demographics and medical history	X								
Review COVID-19 criteria	X								
In- and exclusion criteria	X								
ABO D typing^1^	X								
**Study intervention**									
Randomization		X							
Administration of Plasma				X	X				
**Study procedures**									
Vital signs including SpO2		X	Daily until discharge						
Clinical data collection		X	Daily until discharge						
Targeted medication review		X	Daily until discharge						
Targeted adverse event evaluation when it occurs		X	Daily until discharge						
Electrocardiogram (ECG)		X							
Evaluation by telephone							X	X	X
**Laboratory**									
CRP, haematology, chemistry, kidney and liver test	X	At clinician’s discretion	At clinician’s discretion						
Pregnancy test for females of childbearing potential	X								
Viral qPCR (Nasopharyngeal swab)	X					If feasible			
Blood for COVID-19 antibody titre testing and immunoparesis (optional) ^2^	X					X			
Quality of life (QoL) scoring^3^	X							X	X

^1^ABO D typing has to be performed twice at two different, independent time points and the two ABO D results have to be identical before the blood institution can release the plasma units. Before randomization, the ABO D typing should be done at least once and the result should be known

^2^One serum tube of 10 mL and one EDTA tube of 10 mL. This blood is drawn if feasible and in sites that agree to participate

^3^QoL scoring using the EQ-5D-5L questionnaire. QoL scoring at day+ 30 is optional if the patient is still hospitalized; if the patient is at home at day+ 30, scoring will be done by telephone call

#### Sample size {14}

Despite rapid dissemination of data from clinical case series and some early-stage clinical trials, detailed information about the course of the disease is limited in this stage of the COVID-19 pandemic. The sample sizes presented here are only illustrative.

Furthermore, in the absence of treatments with a known benefit, rapid changes in standard of care are to be expected and important signs of a benefit or harm of a treatment under investigation will require rapid reporting. Safety issues will be continuously monitored by a Data and Safety Monitoring Committee (DSMB), and if at any stage evidence emerges that any one treatment arm is definitely inferior it can be decided that the study will be discontinued. Conversely, if good evidence emerges while the trial is continuing that some other treatment(s) should also be being evaluated, then it can be decided that one or more extra arms or strata will be added while the trial is in progress.

Around 20% of patients hospitalized for a SARS-CoV-2 infection are admitted to the ICU with respiratory failure. When admitted to the ICU, 80% of these patients need mechanical ventilation. With the administration of convalescent plasma as early as possible, we hope to decrease the proportion of patients who have a clinical decline and need ICU support. We assume by providing passive immunity with convalescent plasma, we are able to reduce the proportion of patients admitted to the ICU from 20 to 15%. Furthermore, we assume that, even when admitted to ICU, the need for mechanical ventilation will be reduced from 80 to 50%. With a power of 0.8, a delta of 8.5% (16% in the control group and 7.5% in the invention group), a randomization ratio of 2:1 and an alpha of 0.05, sample size estimates to detect a difference between both groups is 483 patients with 322 patients and 161 patients in the intervention and standard of care group, respectively (using a Pearson chi-square test for proportion difference). We therefore propose these numbers as a pragmatic initial sample size.

Outcomes are in line with other studies with convalescent plasma that are being set up or just started recruitment in other countries. A huge effort is now made on an international level to combine all results of other studies studying the added value of convalescent plasma. All results will be made available in real-time in an international database. This allows to make sound scientific conclusions in a limited time frame. At the time this international database is available, an amendment will be made to the institutional review board.

#### Recruitment {15}

All patients diagnosed with COVID-19 are screened at the emergency ward or upon arrival at the COVID-ward. An emergency physician or a physician at the COVID-ward that is trained in the protocol will first assess the patient’s interest in the study. In case a patient is interested to participate, the investigators of the DAWn-study team again check the eligibility criteria and contact the patient to provide more information and obtain informed consent. By doing so, every patient that gets hospitalized with COVID-19 is screened upon admission.

## Assignment of interventions: allocation

### Sequence generation {16a}

A randomization procedure through a computerized system (REDCap) has been established, generated by the data management unit of the clinical trial centre Leuven, which ensures the integrity of the trial.

### Concealment mechanism {16b}

In this open-label study, patients are allocated in order of diagnosis using a computerized system (REDCap) applying an unknown allocation sequence.

### Implementation {16c}

The investigators will randomize the patients using a computerized system (REDCap).

## Assignment of interventions: blinding

### Who will be blinded {17a}

The study is an open-label study, the study participants and clinical staff are not blinded.

### Procedure for unblinding if needed {17b}

N/A.

## Data collection and management

### Plans for assessment and collection of outcomes {18a}

Source data will be collected and recorded in the participant’s files/medical records. Worksheets may be used for capturing some specific data in order to facilitate the completion of the electronic case report form (eCRF). Any such worksheets will become part of the participant’s source documentation and will be filed together with or as part of the medical records (during but also following completion of the trial).

It remains the responsibility of the Investigator to check that all data relating to the trial, as specified in the trial protocol, are entered into the eCRF in accordance with the instructions provided and that the forms are filled out accurately, completely and in a timely manner.

eCRFs are provided by the sponsor for each participant. A copy of the eCRF is attached to this manuscript as Supplementary Material. The trial data will be transcribed from the source records (i.e. participant’s medical file or trial-specific source data worksheets) into an eCRF by trial staff. Transcription to the eCRF will be done as soon as possible during hospitalization or after a participant visit or telephone contact and in a pseudonymized manner using a unique identifier assigned by the sponsor.

### Plans to promote participant retention and complete follow-up {18b}

If patients are discharged from the hospital, they may be called at certain time points in order to have sufficient data for all endpoints previously described. The same data will be collected from participants where a protocol violation has occurred.

### Data management {19}

Data are stored and pseudonymized in the REDCap system allowing secure and General Data Protection Regulation (GDPR)-compliant data handling. The trial data manager will perform extensive consistency checks on the received data. Queries will be issued in case of inconsistencies in accordance with internal procedures. A Data Management Plan (DMP) will be developed to map data flows, data validation measures that will be taken, how (interim) database lock(s) will be managed and, as applicable, the role and responsibilities of the DSMB.

### Confidentiality {27}

The trial will be conducted in compliance with the requirements of the EU GDPR 2016/679, the relevant Belgian laws implementing the GDPR including the Belgian Privacy Act of 30 July 2018 on the protection of privacy in relation to the processing of personal data. Any collection, processing and disclosure of personal data, such as participant health and medical information, is subject to compliance with the aforementioned personal data protection laws (cfr. Data Processing Annex (DPA) in Appendix I of the protocol provided as an appendix to this paper). In case personal data is transferred outside the European Economic Area, safeguards will be taken to ensure that appropriate protection travels with the data in accordance with the GDPR.

Any personal data shall be treated as confidential at all times including during collection, handling and use or processing and the personal data (including in any electronic format) shall be stored securely at all times and with all technical and organizational security measures that would be necessary for compliance with EU and national data protection legislation (whichever is more stringent). The sponsor shall take appropriate measures to ensure the security of all personal data and guard against unauthorized access thereto or disclosure thereof or loss or destruction while in its custody.

### Plans for collection, laboratory evaluation and storage of biological specimens for genetic or molecular analysis in this trial/future use {33}

The following analyses will be performed on samples collected during the trial:

- Immunoparesis: Serum samples from day 0 (baseline) and day 6 are temporarily stored at the study sites and sent in batch to four collaborating laboratories: REGA institute Leuven, Sciensano, University of Liège and Institute of Tropical Medicine Antwerp. A CPE-based neutralization test is performed on serum to detect SARS-CoV-2 neutralizing antibodies, as the assay is considered the standard for coronavirus serologic analysis.
- Quantitative PCR analysis: Qualitative PCR analysis (nasopharyngeal swab or bronchoalveolar lavage) is performed on day 0 and day 6 as mentioned before. The rest medium of this is temporarily stored at the study sites and consequently sent in batch to the biobank of the central study site (UZ Leuven) to perform quantitative PCR analysis at a later stage.

## Statistical methods

### Statistical methods for primary and secondary outcomes {20a}

This is an open-label controlled randomized trial testing a superiority hypothesis with a two-sided type I error rate of 0.05. In this exploratory study, secondary hypotheses will be tested in a non-hierarchical way. These will be described according to the appropriate summary statistics (e.g., proportions for categorical data, means with 95% confidence intervals for continuous data, median for time-to-event data). A statistical analysis plan (SAP) will be developed and filed with the study sponsor prior to database lock.

The study design allows standard care or best supportive care to be changed in function of the Belgian Sciensano recommendations for treating COVID-19. Standards of care may rapidly change in pandemic situations, even during the enrolment of study participants. For differences between centres, or new recommendations for standard care, statistical adjustments will be made in the analysis.

The following analysis sets will be defined:

- Full analysis set (FAS) and modified-intention-to-treat (mITT, an intention-to-treat analysis allowing for justified exclusion of some randomized individuals, e.g. in the case of post-randomization considered ineligibility): The FAS will include all randomized patients according to their randomized treatment. Patients randomized to the interventional group will be excluded if they did not receive any dose of study blood product. The FAS will be used for the evaluation of all efficacy endpoints. mITT will be applied as primary analysis and FAS as secondary analysis.
- Safety set (SS): The SS will include all patients who were randomized according to their actual treatment. Patients randomized to the interventional group who did not receive any study treatment will be included in the SOC group. The SS will be used for the evaluation of all safety parameters.

1. Analysis of the primary efficacy endpoint: number of patients alive without mechanical ventilation at day 15 after randomization.

The null hypothesis tested is that the proportion of patients that meet the primary endpoint is equal in the standard of care and experimental treatment arm. The alternative hypothesis is that those proportions are different. The primary analysis will be based on a Cochran–Mantel–Haenszel test, stratified for centre. As a secondary analysis, a logistic mixed model will be used with random centre effect and random centre by treatment interaction, allowing to study how the treatment effects vary between centres.

2. Analysis of the secondary endpoint(s)

For the analysis of the WHO outcome, used as a sensitivity analysis, we use a cumulative clinical severity score, based on the 10-category ordinal scale, for the first 15 days (or other time points, based on the blinded interim analysis as described above). This score is calculated by adding the daily severity score (highest score for that day) for each day from day 1 to day 15, thus providing a cumulative measure of disease severity during the course of the disease. Appropriate methods will be used to account for patients for whom the status is not known for the full 15 days.

The null hypothesis being tested is that the mean cumulative clinical severity score during the first 15 days is the same for the standard of care and experimental treatment arms. Because means of summed scores over a number of days are expected to be symmetrically distributed, we will use a *t* test to compare the mean cumulative clinical severity score on day 15 between the treatment and the standard of care group. The treatment effect will be estimated by the difference in mean scores between the treatment groups.

As a co-WHO outcome, according to the WHO suggestions, we will use the ordinal scale to estimate a proportional odds model. For this model, the primary hypothesis test will be based on a test of whether the common odds ratio for treatment is equal to one. For large sample sizes, the hypothesis test is nearly the same as the Wilcoxon rank-sum test.

Therefore, the procedure produces a valid *p* value regardless of whether the proportional odds model is correct. Nonetheless, estimation and confidence intervals do require the model to be correct. Accordingly, we will evaluate model fit using a goodness-of-fit likelihood ratio test. A stratified hypothesis test to account for baseline severity of the disease will be used. The treatment effect will be estimated by the common odds ratio obtained from the proportional odds logistic regression model.

- All-cause mortality rates will be estimated by the treatment group using the Kaplan-Meier method. The resulting Kaplan-Meier curves will be compared using a log-rank test. The treatment effect will be estimated by the hazard ratio using a Cox regression.
- Time-to-event parameters with competing risk (time to clinical improvement, composite cardiac endpoint): event rates will be estimated using cumulative incidence functions (CIF), the resulting CIF curves will be compared using Gray’s test. The treatment effect will be estimated by the subdistribution hazard ratio.
- Duration of hospital and ICU stay: both parameters will be analysed as time-to-event parameters with competing risk, whereby the event of interest is discharge from hospital/ICU and the competing risk is hospital/ICU death.
- Continuous normally distributed variables (e.g. QTc) will be analysed using a 2-sample *t* test. Treatment effects will be estimated by the difference in mean values between the groups. If applicable, changes from baseline will be calculated. Comparisons between treatment groups will be done by performing an analysis of covariance (ANCOVA) on the post-baseline value, using the baseline value as a covariate.
- Continuous non-normally distributed variables (clinical status, National Early Warning Score (NEWS) score, duration of supplemental oxygen, duration of mechanical ventilation) will be analysed using a Wilcoxon rank-sum test. Change in ordinal scale at specific time points will be compared using Wilcoxon rank-sum tests.

Missing data procedures will be described in the SAP.

3. Safety analyses

Adverse events (AE) will be analysed univariately and as a composite endpoint. Time-to-event methods will be used for death and the composite endpoint. Each adverse event will be counted once for a given participant and graded by severity and relationship to COVID-19 or study intervention.

Adverse events leading to premature discontinuation from the study intervention and serious treatment-emergent AEs will be described as part of the primary publication of the study results.

4. Baseline descriptive statistics

Baseline characteristics will be summarized by treatment arm. For continuous measures, the mean and standard deviation will be summarized. Categorical variables will be described by the proportion in each category (with the corresponding sample size numbers).

### Interim analyses {21b}

No formal interim analysis will be planned. A DSMB will monitor ongoing results to ensure patient well-being and safety as well as study integrity. The DSMB, containing one statistician, will have the opportunity to ask for data, to require analyses or to compare Belgian trial results with results available from other countries. The DSMB will be asked to recommend early termination or modification only when there is clear and substantial evidence of a safety issue.

Early analyses include monitoring enrolment, baseline characteristics and follow-up rates throughout the course of the study by the study team. Analyses will be conducted blinded to treatment assignment by the DSMB.

### Methods for additional analyses (e.g. subgroup analyses) {20b}

Subgroup analyses for the primary and selected secondary outcomes will evaluate the treatment effect across the following subgroups: duration of symptoms prior to enrolment, age groups, disease severity at baseline, presence of COVID-19 antibodies prior to convalescent plasma administration and co-morbidities. A forest plot will display confidence intervals across subgroups. Interaction tests will be conducted to determine whether the effect of treatment varies by subgroup.

### Methods in analysis to handle protocol non-adherence and any statistical methods to handle missing data {20c}

In the case of missing data, multiple imputation will be performed. The exact number of iterations will be set when it will be clear how many data are missing. We do not expect much data to be missing as this is a prospective trial with dedicated data management.

### Plans to give access to the full protocol, participant level-data and statistical code {31c}

The full protocol is available as an appendix to this paper. At this time no public access to the patient dataset is planned.

## Oversight and monitoring

### Composition of the coordinating Centre and trial steering committee {5d}

The DAWn-Plasma trial steering committee consists of specialists in the field of haematology, infectiology, respiratory diseases, intensive care, general internal medicine, haemovigilance and biostatistics. The committee is composed in such a way that the different key role playing institutions of the DAWn-Plasma trial are represented.

### Composition of the data monitoring committee, its role and reporting structure {21a}

Monitoring of the trial will be performed by qualified individuals (independent from the site trial staff) according to the monitoring plan. The sponsor and investigator/participating site will permit direct access to the trial data and corresponding source data and to any other trial-related documents or materials to verify the accuracy and completeness of the data collected.

The data safety and monitoring committee (DMC) consists of 6 qualified members, independent from the site trial staff. Their scientific independence is assured through a DMC charter and terms of reference. Because of the exceptional circumstances, the DMC is part of UZ Leuven.

DMC will monitor ongoing results to ensure patient well-being and safety as well as study integrity. It will be asked to recommend early termination or modification only when there is clear and substantial evidence of a safety issue.

### Adverse event reporting and harms {22}

Investigators will seek information on AEs during each patient contact. All events, whether reported by the patient or noted by trial staff, will be recorded in the patient’s medical record and the (e) CRF within a reasonable time after becoming aware. If available, the diagnosis should be reported on the AE form, rather than the individual signs or symptoms. If no diagnosis is available, the investigator should record each sign and symptom as individual AEs.

### Frequency and plans for auditing trial conduct {23}

The investigator will permit direct access to trial data and documents for monitoring, audits and/or inspections by authorized entities such as but not limited to: the sponsor or its designees and competent regulatory or health authorities. As such, eCRFs, source records and other trial-related documentation (e.g. the Trial Master File, pharmacy records) must be kept current, complete and accurate at all times.

### Plans for communicating important protocol amendments to relevant parties (e.g. trial participants, ethical committees) {25}

As per good practice, trial participants will be informed of significant changes during the trial. Yearly updates will be given to the ethical committee.

### Dissemination plans {31a}

The primary paper will be published in a peer-reviewed journal and presented at international meetings.

## Discussion

The administration of convalescent plasma has previously proven to be a promising strategy in enhancing patients’ immune response to infectious diseases such as severe influenza and SARS. Whether or not this approach could help avoiding serious complications from SARS-CoV-2 infection is currently unknown, and uncertainty exists regarding the most appropriate timing and dose of convalescent plasma to be administered in that setting. In this trial, we have chosen the administration of a high dose of CCP, with 4 units being administered within a 36-h timeframe. Additionally, donors are required to have high neutralizing antibody titres of at least 1/320, which is a demanding and strict requirement because we hypothesize that high antibody titres are important for the efficiency of the CCP-treatment. The titres reached in the patient receiving the plasma are monitored throughout the study. Moreover, in order to optimize the interpretability of results coming from different sites, a study in which the four laboratories performing the neutralization tests on serum for the DAWn-Plasma trial are correlating their results is currently ongoing. A high quality of the plasma used in this trial is therefore ensured and carefully monitored. Additionally, full characterization of CCP will be performed later on, to further investigate the specific role of neutralizing antibodies and non-neutralizing antibodies in the plasma.

With regards to the timing of treatment, the DAWn-Plasma investigators have chosen to administer the convalescent plasma in an early time-frame after diagnosis and hospitalization for COVID-19. Randomization is allowed for up to 72 h after COVID-19 diagnosis, and the first units are administered within 12 h after randomization to the active treatment arm. A CCP-unit is infused over 3 h, which was deemed the safest among standard procedures of plasma transfusion by the investigators of this trial.

This trial is a prospective randomized controlled trial. In the past, non-randomized convalescent plasma trials have been carried out for other viral infections [11, 12], so there is an urgent need for prospective randomized trials to prove the efficacy (or not) of convalescent plasma therapy. Currently, patients are being enrolled in 20 sites with a possibility of expanding to a total of 25 sites. The participating hospitals are geographically spread across the country. This will help in faster accrual of the number of patients required for this study and is an advantage especially in the context of the expected evolution of the pandemic, with local flare-ups of SARS-CoV-2 infection rates. Also, convalescent plasma can be obtained from local donors and the Red Cross (both Rode Kruis-Vlaanderen and Croix-Rouge Service du Sang) has long-standing experience with the distribution of blood products to different sites.

At UZ Leuven, this trial is coordinated together with other trials of the DAWn consortium in a central command unit. Qualified personnel, existing of both Medical Doctors and Clinical Trial Assistants, screen newly diagnosed and hospitalized patients for eligibility criteria for the DAWn trials. They are trained in the protocols and coordinate the inclusion of patients based on procedures agreed upon between investigators beforehand. This central command unit is a useful structure to ensure continuous availability of trained personnel. Not only is the collaboration between investigators within UZ Leuven well-coordinated, the collaboration of the DAWn-Plasma team with the different parties involved -such as the different participating sites, the blood establishments and the laboratories- is also coordinated on a high level and runs efficiently.

The limitations of the study are the following: (1) the DAWn-Plasma trial is an open-label study. (2) The informed consent of the patient is not obtained through standard procedures because of safety measures that restrict the transfer of paper documents out of the COVID-19 ward. However, a procedure has been put in place to ensure the patient is fully informed and his/her verbal consent is witnessed by an independent person, and written informed consent is additionally sent to the investigators later on. (3) Another possible limitation of this study is that the novelty of this disease results in rapidly changing guidelines on the standard of care for treating these patients and that this might influence the DAWn-Plasma trial and potentially impose adaptations in the study protocol. (4) And lastly, the unpredictability of the COVID-19 pandemic leads to uncertainties regarding inclusion rates for this study, as well as to uncertainty regarding event rates in case good treatment strategies would become available.

## Trial status

The first patient was included on the 2nd of May 2020. We expect enrolment to be completed by July 2021, provided that the pandemic remains active in Belgium.

## Supplementary Information

**Additional file 1.**

**Additional file 2.**

**Additional file 3.**

**Additional file 4.**

## Data Availability

After the completion of the study the sponsor will transfer a copy of the pseudonymized study data set to the KCE.

## Abbreviations

(e)CRF: (electronic) Case Report Form
(q)PCR: (quantitative) Polymerase Chain Reaction
AE: Adverse event
ADE: Antibody-dependent enhancement
ANCOVA: Analysis of covariance
ARDS: Acute respiratory distress syndrome
CCP: COVID-19 convalescent plasma
CIF: Cumulative incidence functions
DMC: Data safety and Monitoring Committee
DMP: Data Management Plan
DSMB: Data and Safety Monitoring Committee
EC: Ethics Committee
ECG: Electrocardiogram
ECMO: Extra-corporeal membrane oxygenation
FAS: Full analysis set
GDPR: General Data Protection Regulation
ICU: Intensive care unit
IMP: Investigational medicinal product
mITT: Modified-intention-to-treat
NEWS: National Early Warning Score
KCE: Belgian Health Care Knowledge Centre
RCT: Randomized controlled trial
QoL: Quality of life
SAE: Serious adverse event
SAP: Statistical analysis plan
SOC: Standard of care/best supportive care
SOP: Standard operating procedure
SpO2: Oxygen saturation
SS: Safety set
WHO: World Health Organization
