# Correction to: A randomized, multicentre, open-label phase II proof-of-concept trial investigating the clinical efficacy and safety of the addition of convalescent plasma to the standard of care in patients hospitalized with COVID-19: the Donated Antibodies Working against nCoV (DAWn-Plasma) trial

**DOI:** 10.1186/s13063-020-04947-2

**Published:** 2020-12-14

**Authors:** Timothy Devos, Tatjana Geukens, Alexander Schauwvlieghe, Kevin K. Ariën, Cyril Barbezange, Myriam Cleeren, Veerle Compernolle, Nicolas Dauby, Daniël Desmecht, David Grimaldi, Bart N. Lambrecht, Anne Luyten, Piet Maes, Michel Moutschen, Marta Romano, Lucie Seyler, Michel Toungouz Nevessignsky, Katleen Vandenberghe, Johan van Griensven, Geert Verbeke, Erika Vlieghe, Jean Cyr Yombi, Laurens Liesenborghs, Peter Verhamme, Geert Meyfroidt

**Affiliations:** 1 University Hospitals Leuven (UZ Leuven), Leuven, Belgium; 2 Catholic University of Leuven (KU Leuven), Leuven, Belgium; 3 Universitair Ziekenhuis Gent, Ghent, Belgium; 4 Instituut voor Tropische Geneeskunde, Antwerp, Belgium; 5 Sciensano, Elsene, Belgium; 6 Rode Kruis Vlaanderen, Mechelen, Belgium; 7 Universite Libre de Bruxelles Institut d ‘Immunologie Medicale, Bruxelles, Belgium; 8 Universite de Liege, Liege, Belgium; 9 Universite Libre de Bruxelles, Bruxelles, Belgium; 10 Leuven Coordinating Centre, Leuven, Belgium; 11 Katholieke Universiteit Leuven Rega Institute for Medical Research, Leuven, Belgium; 12 Universitair Ziekenhuis Brussel, Bruxelles, Belgium; 13 Croix Rouge de Belgique, Bruxelles, Belgium; 14 Interuniversity Institute for Biostatistics and statistical Bioinformatics, Leuven, Belgium; 15 Universitair Ziekenhuis Antwerpen, Antwerpen, Belgium; 16 Cliniques Universitaires Saint-Luc, Sint-Lambrechts-Woluwe, Belgium

**Correction to: Trials 21, 981 (2020)**

**https://doi.org/10.1186/s13063-020-04876-0**

Following publication of the original article [1], we were notified of a typing mistake in one of the author names.

Originally published name: Alexander Schauvlieghe.

Corrected name: Alexander Schauwvlieghe.

The original article has been corrected.
