# Supplementary material for: A randomized, multicentre, open-label phase II proof-of-concept trial investigating the clinical efficacy and safety of the addition of convalescent plasma to the standard of care in patients hospitalized with COVID-19: the Donated Antibodies Working against nCoV (DAWn-Plasma) trial
Source: Trials. 2020 Nov 27;21:981. doi: 10.1186/s13063-020-04876-0 (PMC7691949; doi:10.1186/s13063-020-04876-0)
Supplement: Supplementary file 1 — Additional file 1. [file 13063_2020_4876_MOESM1_ESM.docx]

## DAWn-Studies: Collaborators

The DAWn study team is grateful to all patients, their family members and all health care workers who helped to perform clinical studies in extraordinary and challenging times during this COVID-19 pandemic, and by doing so have contributed to fighting SARS-CoV-2.

| **Steering Committee DAWn-studies** | |
| --- | --- |
| Eric Van Wijngaerden | Coordinating Investigator D-Itraco |
| Wim Janssens | Coordinating Investigator D-Azithro |
| Thomas Vanassche | Coordinating Investigator D-Antico |
| Geert Meyfroidt | Coordinating Investigator D-Plasma |
| Peter Verhamme | DAWn-RCT coordinator (chair) |
| Laurens Liesenborghs | Principal investigator D-Itraco |
| Robin Vos | Principal investigator D-Azithro |
| Timothy Devos | Principal investigator D-Plasma |
| Jan Gunst | On behalf of D-Antico |
| Joost Wauters | D-Antico and D-Itraco investigator |
| Paul De Munter | D- Itraco investigator |
| Johan Neyts | D-Itraco investigator / Expert virologist |
| Carine Wouters | D-Antico investigator |
| Steffen Rex | D-Antico investigator |
| Lieven Dupont | D-Azithro investigator |
| Isabel Spriet | Expert pharmacologist |
| Geert Verbeke | Senior study statistician |
| Kathleen Claes | Clinical Operational Director, on behalf of sponsor |
| Wim Robberecht | CEO UZ Leuven, on behalf of sponsor |
| Chris Van Geet | Vice-Rector Biomedical sciences, on behalf of sponsor |
|  |  |
| **Advisory Committee** | |
| Willy Peetermans | Chairman Internal Medicine |
| Stefan Janssens | Head of Cardiovascular Diseases |
| Greet Van den Berghe | Head of Intensive Care Medicine |
| Katrien Lagrou | Laboratory Medicine |
| Peter Vandenberghe | Head of Haematology |
| Geert Verleden | Head of Respiratory Diseases |
| Dirk Kuypers | Head of Nephrology |
| Sandra Verelst | Head or Emergency Medicine |
| Marc Van de Velde | Head of Anesthesiology |
| Marc Van Ranst | Head of Laboratory Medicine |
|  |  |
| **Study Coordinators DAWn-studies** | |
| Barbara Debaveye | Clinical Trial Coordinator |
| Helga Ceunen | Clinical Trial Coordinator |
| Veerle Servaes | Study Coordinator D-Antico |
| Anna Ockerman | Study Coordinator D-Antico |
| Caroline Devooght | Study Coordinator D-Antico |
| Maylorie 't Lam | Study Coordinator D-Azithro |
| Kaat Haesendock | Study Coordinator D-Azithro |
| Myriam Cleeren | Study Coordinator D-Plasma |
| Jill Pannecoucke | Study Coordinator D-Plasma |
| Elisabeth Porcher | Study Coordinator D-Plasma |
| Katrien Cludts | Study Coordinator |
| Evelyn Marcelis | Study Coordinator |
| Annemie Devroye | Study Coordinator |
| Sophie Achten | Study Coordinator |
|  |  |
| **Leuven Coordinating Centre (LCC)** | |
| Rik Hendrickx |  |
| Anne Luyten |  |
| Katleen Vandenberghe |  |
| Peter Van Rompaey |  |
|  |  |
| **COVID-19 Clinical Coordinators UZ Leuven** | |
| Willy Peetermans | Department of General Internal Medicine |
| Alexander Wilmer | Department of General Internal Medicine |
| Tom Adriaenssens | Department of Cardiovascular Diseases |
| Stefanie Vandervelden | Department of Emergency Medicine |
| Philippe Dewolf | Department of Emergency Medicine |
| Marijke Peetermans | Department of General Internal Medicine |
| Philippe Meersseman | Department of General Internal Medicine |
| Christophe Vandenbriele | Department of Cardiovascular Diseases |
| Liesbeth Henckaerts | Department of General Internal Medicine |
| Greet Hermans | Department of General Internal Medicine |
| Peter Vanbrabant | Department of General Internal Medicine |
| Nathalie Lorent | Department of Respiratory Diseases |
| Laurent Godinas | Department of Respiratory Diseases |
| Pascal Van Bleyenbergh | Department of Respiratory Diseases |
| Els Wauters | Department of Respiratory Diseases |
| Marion Delcroix | Department of Respiratory Diseases |
| Dieter Dauwe | Department of Intensive Care Medicine |
| Michaël Casaer | Department of Intensive Care Medicine |
| Yves Debaveye | Department of Intensive Care Medicine |
| Lars Desmet | Department of Intensive Care Medicine |
| Erwin De Troy | Department of Intensive Care Medicine |
| Greet De Vlieger | Department of Intensive Care Medicine |
| Greta Van den Berghe | Department of Intensive Care Medicine |
| Renata Haghedooren | Department of Intensive Care Medicine |
| Catherine Ingels | Department of Intensive Care Medicine |
| Bart Jacobs | Department of Intensive Care Medicine |
| Jan Muller | Department of Intensive Care Medicine |
| Dirk Vlasselaers | Department of Intensive Care Medicine |
| Marc Van de Velde | Department of Anesthesiology |
| Johan De Coster | Department of Anesthesiology |
| An Schrijvers | Department of Anesthesiology |
| Veerle De Sloovere | Department of Anesthesiology |
| Arne Neyrinck | Department of Anesthesiology |
| Astrid Barbé | Department of Anesthesiology |
| Steve Coppens | Department of Anesthesiology |
| Raf Van den Eynde | Department of Anesthesiology |
| Kathleen Fagard | Department of Geriatric Medicine |
| Evelien Gielen | Department of Geriatric Medicine |
| João Pedro Guedelha Sabino | Department of Gastroenterology |
|  |  |
| **Laboratory Medicine UZ Leuven** | |
| Pieter Vermeersch |  |
| Katrien Lagrou |  |
| Ann Verdonck |  |
| Koen Poesen |  |
| Marc Jacquemin |  |
| Soumia Arredouani |  |
|  |  |
| **Radiology Department** | |
| Adriana Dubbeldam |  |
| Walter De Wever |  |
|  |  |
| **Coordinating Investigator Medical Support Team** | |
| Iwein Gyselinck |  |
| Matthias Engelen |  |
| Laure-Anne Teuwen |  |
| Tatjana Geukens |  |
| Vincent Geldhof |  |
| Quentin Van Thillo |  |
| Ewout Landeloos |  |
|  |  |
| Yannick Van Herck |  |
| Anke Van Herck |  |
| Pierre Van Mol |  |
|  |  |
| **Pharmacy Safety Monitoring** | |
| Isabel Spriet |  |
| Lorenz Van der Linden |  |
| Charlotte Quintens |  |
| Greet Van de Sijpe |  |
| Ruth Van Daele |  |
| Matthias Gijsen |  |
|  |  |
| **ECG safety monitoring** | |
| Bert Vandenberk | Department of Cardiovascular Medicine |
| Rik Willems | Department of Cardiovascular Medicine |
|  |  |
| [**Leuven Biostatistics and Statistical Bioinformatics Centre (L-BioStat)**](https://www.kuleuven.be/wieiswie/en/unit/50000696) | |
| Geert Verbeke |  |
| Ann Belmans |  |
| Kris Bogaerts |  |
|  |  |
| **Data Safety Monitoring Board** | |
| Séverine Vermeire | Chair |
| Emmanuel Lesaffre | Statistician |
| Joris Ector | Cardiologist |
| Jan de Hoon | Pharmacologist |
| Patrick Verschueren | Rheumatologist |
| Heidi Sterckx | Secretary |
|  |  |
| **Clinical Trial Center (CTC) UZ Leuven** | |
| Hilde De Tollenaere |  |
| Peter Van Rompaey |  |
| Klara Vlassak |  |
| Heidi Sterckx |  |
| Katrien Boulanger |  |
| Jean-Jacques Derèze |  |
|  |  |
| **Other Support** | |
| Diane De Wyngaert | Administrative support |
| Wouter Cypers | IT |
| Jurgen Silence | IT |
| Alexander Otten | IT |
| Kevin Vits | IT |
| Ruth Storme | Ethical Committee |
| Kathleen Schuyten | Administrative support |
| Nadine Ectors | Biobank |
| Loes Linsen | Biobank |
| Dirk Jochmans | Rega Institute |
| Inge Wullaert | KU Leuven fundraising |
| Nadine Loenders | KU Leuven fundraising |
| Kristine Chapelle | KU Leuven fundraising |
| Bas Aerts |  |
|  |  |
| **All Health Care Workers of all COVID-19 units UZ Leuven** | |

Table 1. Collaborators of the DAWn studies. D-Itraco = DAWn-Itraconazole; D-Azithro= DAWn-Azithromycine; D-Antico = DAWn-Antico; D-Plasma = DAWn-Plasma; D-RCT = DAWn-Randomized Controlled Trails, i.e. D-Itraco, D-Azithro, D-Antico and D-Plasma;
