# Supplementary material for: A randomized, multicentre, open-label phase II proof-of-concept trial investigating the clinical efficacy and safety of the addition of convalescent plasma to the standard of care in patients hospitalized with COVID-19: the Donated Antibodies Working against nCoV (DAWn-Plasma) trial
Source: Trials. 2020 Nov 27;21:981. doi: 10.1186/s13063-020-04876-0 (PMC7691949; doi:10.1186/s13063-020-04876-0)
Supplement: Supplementary file 2 — Additional file 2. [file 13063_2020_4876_MOESM2_ESM.pdf]

CLINICAL TRIAL PROTOCOL

Donated antibodies working against nCoV

DAWN-Plasma

Version number: v 2.3– date 27/04/2020

Sponsor

University Hospitals Leuven (UZ Leuven)

Herestraat 49, B-3000 Leuven

Coordinating Investigator

Professor Dr. Geert Meyfroidt

DAWN study coordination

Professor Dr. Peter Verhamme

Confidentiality Statement

The information in this document is strictly confidential and is available for review to Investigators, potential Investigators and appropriate Ethics Committees, Institutional Review Boards or Competent Authorities. No disclosure should take place without written authorization from the Sponsor.

CLINICAL TRIAL PROTOCOL HISTORY

CTP / Amendment #	Date	Reason for amendment
DAWN-Plasma v1.0	*6-4-2020*	
DAWN-Plasma v2.0	*10-4-2020*	
DAWN-Plasma v2.1	*15-4-2020*	
DAWN-PLASMA V2.2	*24-4-2020*	
DAWN-PLASMA V2.3	*27-4-2020*	

LIST OF PARTICIPATING SITES

(as applicable)

List Of Participating Sites	Principal Investigator
UZ Leuven	Professor Dr Timothy Devos
CHU Brugmann	Dr Evelyne Maillart
Erasmus Ziekenhuis Brussel	Professor Dr David Grimaldi
UZ Brussel	Dr Lucie Seyler
UMC Sint-Pieter Brussel	Dr Nicolas Dauby
AZ Delta Roeselare	Dr Bernard Bouckaert
CHU Liège Sart-Tilman	Professor Dr Michel Moutschen
Cliniques Universitaires St Luc	Professor Dr Jean Cyr Yombi
CHC Liège Mont Légia	Dr Laurent Jadot
Institut Bordet	Dr Georgala Aspasia
ZNA	Dr Niels Van Regenmortel
AZ Groeninge	Dr Matthias Leys
CHR Jolimont Mons-Hainaut	Dr Clotilde Visée
CHR Citadelle Liège	Dr Martial Moonen

SIGNATURES

Title: **Donated antibodies working against nCoV (DAWN-Plasma)**

Protocol: **DAWN-Plasma**

The undersigned confirm that the above referenced protocol has been acknowledged and accepted, and agree to conduct the Trial in compliance with the approved protocol, and will adhere to: the principles outlined in the requirements for the conduct of clinical trials in the EU as provided for in Directive 2001/20/EC or the EU Regulation 536/2014 (as soon as in effect) and any subsequent amendments thereto, the ICH guidelines, the most recent version of the Declaration of Helsinki, the Belgian law of May 7th 2004 regarding experiments on the human person (as amended) or the Belgian law of May 7th 2017 related to clinical trials on medicinal products for human use (as soon as in effect), the EU General Data Protection Regulation 2016/679 (GDPR), the relevant Belgian laws implementing the GDPR, the Belgian Law of August 22nd 2002 on patient rights, the Sponsor's applicable SOPs, and other regulatory requirements as applicable.

The undersigned agree not to disclose the confidential information

contained in this document for any purpose other than the evaluation or conduct of the Trial, without prior written consent of the Sponsor.

The undersigned also commit to making the findings of the Trial publicly available through publication and/or other dissemination tools, in accordance with this protocol and applicable regulations, without any unnecessary delay and to provide an honest, accurate and transparent account of the Trial; and to explain any discrepancies or deviations from the approved Trial protocol.

Coordinating Investigator

Professor Dr Geert Meyfroidt	....	....
Name & Title	Signature	Date

Principal Investigator (Participating Site) *(in case of monocentric Trial, the Principal Investigator is the same as the Coordinating Investigator)*

Professor Dr Timothy Devos

	....	....
Name & Title	Signature	Date

Coordination Dawn trials

Professor Dr Peter Verhamme

	....	....
Name & Title	Signature	Date

TABLE OF CONTENTS

CLINICAL TRIAL PROTOCOL 1
CLINICAL TRIAL PROTOCOL HISTORY 2
LIST OF PARTICIPATING SITES 2
SIGNATURES 3
TABLE OF CONTENTS 4
LIST OF ABBREVIATIONS 7
FUNDING AND SUPPORT 9
ROLES AND RESPONSIBILITIES 9
TRIAL SYNOPSIS 10
TRIAL FLOWCHART DAWN-PLASMA 13
2 Background and Rationale 15
3 Trial Objectives and Design 16
3.1 Trial objectives 16
3.2 Trial outcomes 17
3.2.1 Primary outcome 17
3.2.2 Secondary outcome 17
3.2.3 Exploratory outcomes 18
3.3 Trial Design 18
3.4 Expected Duration of the Trial 19
4 Trial Population / Eligibility Criteria 19
4.1 Inclusion criteria 19
4.2 Exclusion criteria 20
5 Trial Procedures 20
5.1 Participant Consent and withdrawal of consent 20
5.2 Selection of Participants / Recruitment 21
5.3 Randomization Procedure 21
5.4 Trial Procedures 21
5.4.1 By visit 21
5.4.2 Laboratory tests 22
5.4.3 Other investigations 22
5.4.4 Exploratory investigations 22
5.5 Premature discontinuation of Trial treatment 23
6 Trial Intervention 24
6.1 Collection of convalescent plasma 24
6.2 Convalescent Plasma and Dosing Regimen 25
6.3 Concomitant / Prohibited Medication / Treatment 26

7 Safety 27
7.1 Specification, timing and recording of safety parameters 27
7.1.1 Definitions 27
7.1.2 Adverse Events that do not require reporting 27
7.1.3 Recording and reporting of Adverse Events 28
7.1.4 Assessment 28
7.1.5 Timelines for reporting 29
7.1.6 Follow-up 29
7.1.7 Death 29
7.1.8 Reporting requirements to Ethics Committee's (EC's) and Competent Authorities (CA's) 29
7.1.9 Sponsor's reporting of Suspected Unexpected Serious Adverse Reactions (=SUSARs) 29
7.1.10 Annual reporting 30
7.1.11 Data and safety monitoring board (DSMB) and Treatment stopping rules 30
8 Statistics and Data Analysis. 31
8.1 Sample Size Determination 31
8.2 Statistical Analysis 32
8.3 Data & Safety Monitoring Board (DSMB) 34
9 Data handling 35
9.1 Data Collection Tools and Source Document Identification 35
9.1.2 Legal requirements 35
9.2 Audits and Inspections 36
9.3 Monitoring 36
9.4 Archiving 36
10 Ethical and Regulatory Considerations 37
10.1 Ethics Committee (EC) review & reports 37
10.2 Regulatory Compliance 37
10.3 Protocol / GCP compliance 37
10.4 Data protection and participant confidentiality 37
10.5 Insurance 38
10.6 Access to the Study Data by KCE and similar institutes in the EU 38
10.7 Amendments 39
10.8 Post-Trial activities 39
11 Research Registration, Dissemination of Results and Publication Policy 40
12 Intellectual Property 40
13 Joint Commission International (JCI) 40
References 41
APPENDICES 43
14 Appendix 1: Data Processing Annex (DPA) 43

Definitions: 43

LIST OF ABBREVIATIONS

Abbreviation	Definition
(e)CRF	(electronic) Case Report Form
AE	Adverse Event
AESI	Adverse Event of Special Interest
APR	Annual Progress Report
ASR	Annual Safety Report
AR	Adverse Reaction
CA	Competent Authority
CI	Coordinating Investigator
CIOMS	Council for International Organizations of Medical Sciences
CM	Concomitant Medication
CSR	Clinical Study Report
CTP	Clinical Trial Protocol
DMP	Data Management Plan
DPA	Data Processing Annex
DSMB	Data and Safety Monitoring Committee
DSUR	Development Safety Update Report
EC	Ethics Committee
ECG	Electrocardiogram
EoT	End of Trial
FPFV	First Patient First Visit
GCP	Good Clinical Practice (latest version of ICH E6)
IB	Investigator's Brochure
ICF	Informed Consent Form
ICH	International Conference on Harmonisation
ISF	Investigator Site File
LPLV	Last Patient Last Visit
MAH	Marketing Authorisation Holder
MP	Monitoring Plan
PI	Principal Investigator (Participating Site)
PRO	Patient Reported Outcome
SAE	Serious Adverse Event
SAP	Statistical Analysis Plan
SAR	Serious Adverse Reaction
SOC	Standard of Care / Best supportive care
SmPC	Summary of Product Characteristics
SUSAR	Suspected Unexpected Serious Adverse Reaction
TMF	Trial Master File

TSC	Trial Steering Committee

FUNDING AND SUPPORT

Funder	Type of Financial or Non-Financial Support

No fault liability insurance has been taken out by UZ Leuven for treating and/or compensating Trial participants who are harmed as a consequence of participation in the Trial.

ROLES AND RESPONSIBILITIES

The Principle Investigator (PI) is responsible for the conduct of the Trial at his/her Participating Site, and for protecting the rights, safety and well-being of the Trial participants. As such the PI must ensure adequate supervision of the Trial conduct at the Participating Site. If any tasks are delegated, the PI will maintain a log of appropriately qualified persons to whom he/she has delegated specified Trial-related duties. The PI will ensure that adequate training is provided and documented for all Trial staff, prior to conducting assigned Trial-related activities.

It is the Coordinating Investigator's (CI's) responsibility to supervise the general conduct (e.g. Trial progress, communication, protocol training and support of the participating sites, annual reporting to the Ethics Committee (EC), end of Trial notification(s) and results reporting…) of the Trial. The CI fulfils both Investigator and Sponsor responsibilities, as outlined in International Conference on Harmonisation – Good Clinical Practice (ICH-GCP) E6(R2) and applicable regulations.

PI and CI shall each be referred to as « Investigator(s)».

TRIAL SYNOPSIS

Title of clinical Trial («Trial»)	Donated antibodies working against nCoV (DAWN-Plasma)
Protocol Short Title Acronym	**DAWN-Plasma**
Trial Phase (I, II, III, IV)	phase II proof-of-concept study
Sponsor name	University Hospitals Leuven (UZ Leuven)
Coordinating Investigator	Prof. Dr. Geert Meyfroidt
Contact Address CI	Herestraat 49, 3000 Leuven (UZ Leuven)
E-mail	geert.meyfroidt@uzleuven.be
Phone	+32 16 34 40 21
Medical condition or disease under investigation	COVID-19
Trial rationale	To evaluate clinical efficacy and safety of convalescent plasma for COVID-19
Primary objective	The overall objective of the study is to evaluate the clinical efficacy and safety of best supportive care combined with convalescent plasma versus best supportive care only in patients hospitalized with COVID-19.
Secondary objective(s)	To evaluate clinical efficacy of convalescent plasma vs. SOC as assessed by Clinical Severity, Oxygenation, Ventilation, Hospitalisation.
Trial Design	Randomized, Open-label, Multicentre, Adaptive Study design
EudraCT number	N/A
Other public database nbr	/
Principal Investigators and Participating Sites	University Hospitals Leuven (UZ Leuven): local PI = Prof. Dr. Timothy Devos<br><br>Cfr list op participating sites page 2
Medical condition or disease under investigation	COVID-19
Outcomes	**Primary outcome**<br>Patients requiring mechanical ventilation or death<br><br>**Key secondary outcome**<br>Clinical status of subject at day 15 and day 30 (on a 10-point "WHO progression" ordinal scale):<br>0. Uninfected. Non viral RNA detected<br>1. Ambulatory, Asymptomatic, viral RNA detected<br>2. Ambulatory, Symptomatic, Independent<br>3. Ambulatory, Symptomatic, Assistance needed<br>4. Hospitalized, mild disease, No oxygen therapy needed<br>5. Hospitalized, mild disease, Oxygen by mask of nasal prongs<br>6. Hospitalized, severe disease, Oxygen by NIV or High flow<br>7. Hospitalized, severe disease, Intubation and mechanical ventilation (pO2/FiO2>=150 OR SpO2/FIO2>=200)

	8. Hospitalized, severe disease, Mechanical ventilation (pO2/FiO2<150 OR SpO2/FIO2<200) OR vasopressors (norepinephrine >0.3 microg/kg/min)<br>9. Hospitalized, severe disease, Mechanical ventilation pO2/FiO2<150 AND vasopressors (norepinephrine >0.3 microg/kg/min), OR Dialysis OR ECMO<br>10. Death, Dead

Sample Size	483 patients with 2:1 randomization<br>• 322 patients receiving plasma<br>• 161 patients receiving SOC
Blood product, dosage and route of administration	4 units of convalescent plasma:<br>• 2 units of plasma are administered within 12h after randomization, but preferably as soon as practically possible<br>• 2 units of plasma should be administered between 24h and 36h after the first infusion<br><br>Other investigational products may be added as part of the adaptive study design.
Labs conducting neutralizing antibody testing	REGA institute Leuven (Piet Maes); Sciensano (Cyril Barbezange), University of Liège (Daniel Desmecht), ITM (Kevin Arïen).
Active comparator product(s)	none
Maximum duration of treatment and Follow Up of a Participant	90 days of follow-up
Maximum duration of entire Trial	18 months
Date anticipated First Participant First Visit (FPFV)	To be determined
Date anticipated Last Patient Last Visit (LPLV)	Unknown

TRIAL FLOWCHART DAWN-PLASMA

Schedule of Events – Trial specific Procedures / Assessments

	Screen	Baseline							
**Day +/- window**	**-1 or 0**	**0**	**Daily until discharge**	**Within 12 hours after randomization***	**24-36 hours afer 1st administration***	**6 +/- 2**	**15 +/- 2**	**30 +/- 3**	**Day 90+/-5**
**Assesments/Procedures**									
**ELIGIBILITY**									
Informed consent	X								
Demographics & Medical History	X								
Review COVID-19 criteria	X								
In- and exclusion criteria	X								
ABO D typing *[1]	X								
**STUDY INTERVENTION**									
Randomization		X							
Administration of Plasma				X	X				
**STUDY PROCEDURES**									
Vital signs including SpO2		X	Daily until discharge						
Clinical data collection		X	Daily until discharge						
Targeted medication review		X	Daily until discharge						
Targeted Adverse event evaluation when it occurs		X	Daily until discharge						
ECG		X							
Evaluation by telephone							X	X	X

**LABORATORY**									
CRP, haematology, chemistry, kidney and liver test	X	At clinician's discretion	At clinician's discretion						
Pregnancy test for females of childbearing potential	X								
Viral qPCR (Nasopharyngeal swab)	X					If feasible			
Blood for COVID-19 antibody titer testing and immunoparesis (optional) *2	X					X			
Quality of life scoring *3	X							X	X

*1 ABO D typing has to be performed twice at two different, independent time points and the two ABO D results have to be identical before the blood institution can release the plasma units. Before randomisation the ABO D typing should be done at least once and the result should be known.
*2 one serum tube of 10 ml and one EDTA tube of 10 mL. This blood is drawn if feasible and in sites that agree to participate.
*3 Q of L scoring using the EQ-5D-5L questionnaire. QofL scoring at day+30 is optional if the patient is still hospitalized; if patient is at home at day+30, scoring will be done by telephone call.

2 Background and Rationale

In December 2019, the Wuhan Municipal Health Committee identified an outbreak of viral pneumonia cases of unknown cause. Coronavirus RNA was quickly identified in some of these patients. This novel coronavirus has been designated SARS-CoV-2, and the disease caused by this virus has been designated COVID-19. Currently there are no approved therapeutic agents available for coronaviruses[1].

The Direct antivirals working against nCoV (DAWN) master study protocol allow to investigate promising drug compounds or convalescent plasma in a proof-of-concept study. The study complies with the recommendations for outcomes as outlined by the WHO master template protocol (https://www.who.int/emergencies/diseases/novel-coronavirus-2019/technical-guidance/early-investigations; and https://www.who.int/emergencies/diseases/novel-coronavirus-2019/global-research-on-novel-coronavirus-2019-ncov assessed on March 20th 2020). The design is adaptive, i.e. it allows to add and remove treatment arms and or strata for based on the most updated information.

Based on the current information, different strategies hold promise for a successful reduction of COVID-19 disease burden. The first is **anti-viral therapy** to reduce viral replication. The second strategy is to modify the inappropriate **host inflammatory response**. Widespread systemic inflammation and subsequent activation of the coagulation and complement system have repeatedly been described in severe COVID-19[3,4].

Another potentially attractive treatment option for COVID-19 infection is the administration of **convalescent plasma**.

Plasma collected from patients who have recovered from a SARS-CoV-2 infection, contains antiviral antibodies. This could help newly diagnosed patients with SARS-CoV-2 infection to clear the infection more rapidly. This plasma provides passive polyclonal antibody administration and thus immediate immunity. The treatment effect of plasma has already been shown in other viral infections but has not been studied in patients with SARS-CoV-2 infection. A recent meta-analysis has shown that convalescent plasma reduces mortality compared to standard of care in patients with SARS and severe influenza infections. Importantly, these studies were heterogeneous on the timing of plasma adminitstration and the threshold on anti-virus antibodies in convalescent patients. It is particularly successful when administered early after symptom onset (PMID 25030060).

Information on immune response on SARS-CoV-2 infection is rather limited at this point. It seems that seroconversion occurs within 2 weeks after infection. Recently, case series showed that convalescent plasma possibly had a contributory effect on clinical improvement of 5 critically ill patients with SARS-CoV-2 and acute respiratory distress syndrome (PMID 32219428) . This study will evaluate the effect of passive immunotherapy with recovalsescent plasma for the treatment of patients with a newly diagnosed SARS-CoV-2 infection.

However, convalescent plasma has proven not to be effective against other viral infection (e.g. Ebola) and carries a risk of ADE (antibody-dependent enhancement), which could result in worsening of the ARDS. Hence, the efficacy and safety of convalescent plasma should be studied in a randomized controlled setting.

The amount of administered plasma varied in studies investigating the added value of convalescent plasma in the SARS epidemic in 2003. The volume of plasma infused ranged from 160mL to 640mL (PMID 15616839). There is no consensus in literature on the optimal volume of convalescent plasma for maximum treatment effect. In this study, we will investigate the added value of 4 units of plasma. This allows to administer plasma of different donors with a variable titer of anti-COVID 19 antibodies to the same patient, ensuring that enough anti-COVID19 antibiodies will provide passive immunisation.

Eligible adult patients who tested positive for SARS-CoV-2 and are admitted to the hospital will be randomized and assessed daily during hospitalization. Discharged patients will be contacted by telephone at days 15, 30 and 90. All subjects will undergo efficacy and safety assessments, including laboratory assays, which are aligned with clinical care. Only an extra blood sample will be drawn at baseline. Also blood samples and nasopharyngeal swabs will be done according to clinical need (standard of care). If feasible, an additional nasopharyngeal swab will be taken on day 6. The study should not put an extra burden on healthcare workers and on the hospital's resources.

3 Trial Objectives and Design

3.1 Trial objectives

The study objectives are adapted from the WHO master protocol that was proposed to streamline interventional studies in patients with COVID-19. (https://www.who.int/emergencies/diseases/novel-coronavirus-2019/technical-guidance/early-investigations; https://www.who.int/emergencies/diseases/novel-coronavirus-2019/global-research-on-novel-coronavirus-2019-ncov assessed on March 20th 2020).

The overall objective of the DAWN-plasma study is to evaluate the clinical efficacy and safety of convalescent plasma relative to the standard of care in patients hospitalized with COVID-19.

Secondary objectives are to evaluate clinical efficacy of convalescent plasma as compared to the control arm.

Clinical Severity

Ordinal scale:

- time to an improvement of one category from admission on an ordinal scale.
- subject clinical status on an ordinal scale at days 3, 5, 8, 11, 15 and 30.
- mean change in the ranking on an ordinal scale from baseline to days 3, 5, 8, 11, 15 and 30 from baseline.

Oxygenation

- oxygenation free days in the first 30 days (to day 29).
- incidence and duration of new oxygen use during the trial.

Mechanical Ventilation

- the proportion of patients requiring invasive mechanical ventilation
- ventilator free days alive in the first 30 days (to day 29).
- incidence and duration of new mechanical ventilation use during the trial.

Hospitalization

- duration of hospitalization (days).

Mortality

- 15-day mortality
- 30-day mortality
- 90-day mortality

Evaluate the safety of the interventions through 30 days of follow-up as compared to the control arm as assessed by

- transfusion-related adverse reaction
- discontinuation or temporary suspension of drug administration (for any reason).
- cumulative incidence of serious adverse events (SAEs) and adverse events (AEs) graded as severe.

3.2 Trial outcomes

The study outcomes are based on the WHO master protocol.

3.2.1 Primary outcome

Primary outcome of the study is <u>the number of patients alive without mechanical ventilation at day 15 after hospitalization</u>.

<u>Rationale:</u> the primary objective of the study is to avoid further clinical decline by administering convalescent plasma to hospitalized patients early after symptom onset to provide immediate (passive) immunity. The hypothesis is that early administration of plasma will help to counter the clinical deterioration when pathology is mainly driven by viral replication, and hence will prevent the need for mechanical ventilation or death in the first 15 days after randomization. The null hypothesis is that convalescent plasma will not be effective to prevent the need for mechanical ventilation or death. It is of utmost importance to find treatment that reduces ICU demand as current strategies of prolonged distancing likely to have negative social and economic impact (PMID 32291278).

3.2.2 Secondary outcome

1) outcomes as per WHO criteria:

Clinical status of subject at day 15 and day 30 (on a 10-point "WHO progression" ordinal scale):

0. Uninfected. Non viral RNA detected
1. Ambulatory, Asymptomatic, viral RNA detected
2. Ambulatory, Symptomatic, Independent
3. Ambulatory, Symptomatic, Assistance needed
4. Hospitalized, mild disease, No oxygen therapy needed
5. Hospitalized, mild disease, Oxygen by mask of nasal prongs
6. Hospitalized, severe disease, Oxygen by NIV or High flow
7. Hospitalized, severe disease, Intubation and mechanical ventilation (pO2/FiO2>=150 OR SpO2/FIO2>=200)
8. Hospitalized, severe disease, Mechanical ventilation (pO2/FiO2<150 OR SpO2/FIO2<200) OR vasopressors (norepinephrine >0.3 microg/kg/min)
9. Hospitalized, severe disease, Mechanical ventilation pO2/FiO2<150 AND vasopressors (norepinephrine >0.3 microg/kg/min), OR Dialysis OR ECMO
10. Death, Dead

Cumulative clinical status of subject up to day 15 (on a 10-point ordinal scale):
The sum of the daily clinical status score for days 1 up to 15.

Theoretical minimum is 21 (best-case scenario, discharge immediately after enrollment) and theoretical maximum is 105 (death on first study day).

2) secondary outcomes (listing):

- proportion of patients on mechanical ventilation or death within 30 days and 90 days after randomisation.
- status on an ordinal scale assessed daily while hospitalized and on days 15 and 30.
- time to clinical improvement (number days from hospitalization to first 2-point improvement from highest previously recorded clinical state on the 7-point ordinal scale)
- duration of hospitalization
- duration of supplemental oxygen
- duration of mechanical ventilation.
- need for and duration of intensive care stay.
- need for and duration of ECMO.
- date and cause of death (if applicable).
- adverse events graded as severe or SAEs
- venous thromboembolism: deep vein thrombosis and pulmonary embolism
- transfusion related side effects like transfusion related acute lung injury, serious allergic transfusion reactions and transfusion associated circulatory overload.
- correlation between clinical outcome and titer of anti-SARS-Cov-2 neutralizing antibodies in transfused plasma units
- safety of convalescent plasma therapy
- effect of plasma therapy on quality of life 30 days after randomization
- Vital signs:
    - daily highest temperature measured during hospitalization with a maximum of 14 days after hospitalization
    - Highest amount of oxygen given (in L/min) daily during hospitalization with a maximum of 14 days after hospitalization

3.2.3 Exploratory outcomes

- qualitative and quantitative PCR for SARS-CoV-2 in (naopharyngeal) swab on day 6 (when feasible)
- Comparing clinical efficacy of convalescent plasma in patients that already had anti-COVID19 antibody specificities with patients that did not have antibodies before the administration of plasma. These results will not be available real-time but are subject to a post-hoc analysis.

3.3 Trial Design

This **DAWN-plasma** study is a **randomized, open-label clinical trial** to evaluate the safety and efficacy of convalescent plasma in hospitalized adult patients diagnosed with COVID-19.

The outcomes of the study protocol are in part based on the draft master protocol of the WHO for trials that evaluate safety and efficacy of investigational therapeutics for the treatment of COVID-19 in hospitalized patients.

The study is a **phase 2 proof-of-concept multicenter trial**.

The DAWN study will compare standard of care vs. standard of care with convalescent plasma. Since there are no current approved treatment options for COVID-19, the standard of care is mostly supportive. However, the standard of care will reflect the guidance by (inter)national guidelines and hence may change during the course of the study. The clinical outcomes of this study have been choosen based on the outcomes of the WHO master template for clinical studies to allow pooling of the data with other ongoing studies.

The DAWN-PLASMA will randomize with a 2:1 allocation to SOC combined with convalescent plasma versus SOC.

If judged necessary, the trial can be extended to add a third arm to allow the participation of centers participating to other clinical trials. This will be based on appropriate statistical considerations; randomization to this third arm and analysis of this third arm data will then be described in the SAP.
A third arm can be included provided that no selection bias is introduced. This means that sites should not decide up front which patients are eligible for either study as this may introduce bias in allowing patients to enter one study or the other.

3.4 Expected Duration of the Trial

The **DAWN PLASMA** is expected to start April 2020, with a duration of 18 months.

4 Trial Population / Eligibility Criteria

4.1 Inclusion criteria

Participants eligible for inclusion in this Trial must meet **all** of the following criteria:

1. Subject (≥18 years old) or legally authorized representative provides informed consent prior to initiation of any study procedures. When signed informed consent is not possible (e.g. due to restrictions to prevent viral transmission), verbal informed consent in the presence of a witness will be obtained and documented in the medical files. Signed informed consent will be obtained as soon as the safety concerns are mitigated.
2. Subject (or legally authorized representative) understands and agrees to comply with planned study procedures.
3. Male or non-pregnant female adult ≥18 years of age at time of enrolment.
4. Patient should be hospitalized
5. Has a confirmed diagnosis of SARS-CoV-2 infection, defined as *either:*
    a. laboratory-confirmed SARS-CoV-2 infection as determined by PCR, or other commercial or public health assay in any specimen as diagnosed within 60 hours prior to randomization **or**
    b. The combination of upper or lower respiratory infection symptoms (fever, cough, dyspnea, desaturation) **and** typical findings on chest CT scan **and** absence of other plausible diagnoses
6. Illness of any duration, and at least one of the following:
    a. Radiographic infiltrates by imaging (chest x-ray, CT scan, etc.), or

   b. Clinical assessment (evidence of rales/crackles on exam) AND SpO2 ≤ 94% on room air, or
   c. Requiring supplemental oxygen.
7. ABO D typing of the patient should be done at least once and the result should be known.

All participants that are considered for Trial participation, per the above criteria will be documented on the Screening Log, including Screen Failures.

4.2 Exclusion criteria

Participants eligible for this Trial must **not** meet any of the following criteria:

1. Receiving invasive (any mode where a patient has been intubated endotracheally, or via tracheostomy) or non-invasive (for instance, but not restricted to CPAP, PSV, PCV, SiMV) mechanical ventilation before or upon randomization.
2. Pregnancy or breast feeding.
3. Any medical condition which would impose an unacceptable safety hazard by participation to the study.
4. Patients with a documented grade 3 allergic reaction after the administration of fresh frozen plasma (i.e. systemic reaction with cardiovascular and/or respiratory involvement)
5. Patients that have treatment restriction that excludes mechanical ventilation and/or endotracheal intubation

Participants who meet one or more of the above exclusion criteria **must not proceed** to be enrolled/randomized in the Trial and will be identified on the Screening Log as Screen Failure.

5 Trial Procedures

5.1 Participant Consent and withdrawal of consent

The Trial will be conducted only on the basis of prior informed consent by the Trial participants and/or their legally authorized representative(s). As such, no Trial-related procedures will be conducted prior to obtaining written informed consent from potential Trial participants.

When signed informed consent is not permitted because of safety regulations related to the prevention of the transmission of SARS-CoV-2, verbal informed consent shall be documented in the medical records. Signed informed consent shall then be obtained as soon as permitted based on safety regulations to prevent the transmission of SARS-CoV-2.

The process for obtaining and documenting initial and continued informed consent from potential Trial participants will be conducted in accordance with ICH-GCP E6(R2), applicable regulatory requirements and internal Standard Operating Procedures (SOPs).

All originally signed obtained Informed Consent Forms (ICFs) must be retained/archived in the Investigator Site File (ISF) at the Participating Site and must not be destroyed (even when a scanned copy is available) before expiration of the legal archiving term as defined in the protocol section entitled “Archiving”.

Participants may voluntarily withdraw consent to participate in the Trial for any reason at any time. The participant’s request to withdraw from the Trial must always be respected without prejudice or consequence to further treatment. Consent withdrawal will be documented in the participant’s medical

record. The PI must take into account the consequences of such withdrawal: (1) further use of personal data/Trial data, (2) use of human biological materials already collected, (3) safe transition to alternative treatment options, etc. as applicable

5.2 Selection of Participants / Recruitment

Only adult hospitalized patients diagnosed with COVID-19 will be included.

5.3 Randomization Procedure

To ensure the integrity of the Trial, a randomization procedure through a computerized system has been established, generated by the data management unit of the clinical trial center leuven. For the multicentre DAWN-PLASMA study a randomization ratio of 2 convalescent plasma to 1 usual care will be allocated.

5.4 Trial Procedures

5.4.1 By visit

Screening:
Patients with documented COVID-19 who require hospitalization will be screened for eligibility. Informed consent will be obtained. When written informed consent is not possible due to restrictions to prevent the transmission of SARS-CoV-2, oral informed consent will be documented in the medical files, and completed with written informed consent as soon as the restrictions do no longer apply.
Demographic parameters will be obtained. Medical history will be obtained as part or routine clinical care. When study-related procedures impose an additional burden on the clinical care of patients, they can be waived.
A first blood grouping should be done upon hospitalization of the patient to facilitate fast plasma administration following randomization.

Baseline / Randomization:
Parameters should be obtained as part of routine clinical care.
When study related procedures impose an additional burden on the clinical care of patients, they can be waived. Study blood product will be administered when randomized to the investigational blood product arm. Medication will be reviewed using the electronic medical files. Serious adverse events and adverse events grade IV will be collected when these are not outcomes of the study.

Administration of Blood product
Convalescent plasma (2 units of approximately 250mL) will be administered within 12 hours after randomization, with a second administration (2 units of approximately 250mL) 24 to 36 hours after the first administration.

Plasma should not be transfused with the usal speed of 30 to 45 minutes but should be administered at a *maximal speed of 50-100 millilitres* per hour. This will reduce the risk of volume overload significantly.

Daily assessments until discharge:
- Vital signs including SpO2
- Clinical data collection for assessment of study outcomes
- Adverse event evaluation

Serious adverse events and adverse events grade IV will be collected when these are not outcomes of the study. When study-related procedures impose an additional burden on the clinical care of patients, they can be waived.

Visit at Day 15 (+/-2), 30(+/-3) and 90 (+/-5)
These visits can be phone visits when patients are no longer hospitalized.

5.4.2 Laboratory tests

To avoid burden on clinical care in a time of a strained health care system, laboratory tests are part or routine clinical care and are not mandatory, but when available, a targeted set will be collected for exploratory purposes (including but not limited to CRP, total white cell and lymphocyte count, haemoglobin, platelets, creatinine, hsTroponinT, LDH, ferritin, glucose, total bilirubin, ALT, and AST, D-dimers).

If feasible, a blood sample from baseline before plasma transfusion should be stored locally and shipped to the central lab to allow to see whether anti-COVID 19 antibodies are already present and to allow for extra analyses after the outbreak when resourrces are more available.

5.4.3 Other investigations

The study includes two optional samples:

- at baseline: on Day 0 (+/- 2): blood samples for the assessment of immunoparesis against SARS-CoV-2 and antibody clearance. This blood samples should be collected before the first infusion of convalescent plasma. Samples will be stored locally and shipped in batches to the central reference lab. The practical details of local storage and shipment can be found in the lab manual.

- on Day 6 (+/- 2): blood samples for the assessment of immunoparesis against SARS-CoV-2 and antibody clearance. Samples will be stored locally and shipped in batches to the central reference lab. The practical details of local storage and shipment can be found in the lab manual.

The study includes a sample to be taken, when feasible:

- viral qPCR (nasopharyngeal swab) at Day 6 (+/- 2)

(the viral qPCR (nasopharyngeal swab) at baseline, on day 0 (+/- 2), is mandatory)

5.4.4 Exploratory investigations

A telephone call on D90 (+/-5days) will check for hospital admission or survival status.

5.5 Premature discontinuation of Trial treatment

Participants may voluntarily discontinue Trial treatment and/or prematurely end their participation in the Trial for any reason at any time. In such case the Investigator must make a reasonable effort to contact the participant (e.g. via telephone, e-mail, letter) in order to document the primary reason for this decision.

The Investigator may also decide at any time during the course of the Trial, to temporarily interrupt or permanently discontinue the Trial treatment if it is deemed that continuation would be detrimental to, or not in the best interest of the participant.

Similarly, the Sponsor, Ethics Committee or authorized regulatory authority can decide to halt or prematurely terminate the Trial when new information becomes available whereby the rights, safety and well-being of Trial participants can no longer be assured, when the integrity of the Trial has been compromised, or when the scientific value of the Trial has become obsolete and/or unjustifiable.

Circumstances requiring premature treatment interruption or discontinuation of the Trial, include but are not limited to:

- Safety concerns related to Blood product or unacceptable intolerability (potentially life-threatening transfusin reaction during plasma infusion)/
- Trial participation while in violation of the inclusion and/or exclusion criteria
- Pregnancy
- Intention of becoming pregnant
- …

In any such case of early Trial termination and/or treatment interruption/discontinuation, the Investigator will continue to closely monitor the participant's condition and ensure adequate medical care and follow-up.

For participants whose status is unclear because they fail to appear for Trial visits without stating an intention to discontinue or withdraw, the Investigator must make every effort to demonstrate "due diligence" by documenting in the source documents which steps have been taken to contact the participant to clarify their willingness and ability to continue their participation in the Trial (e.g. dates of telephone calls, registered letters, etc.).

A participant should not be considered lost to follow-up until due diligence has been completed.

6 Trial Intervention

DAWN PLASMA

**Generic Drug Name**<br>**(& company brand name)**		**Used within Indication? (Y or N)**
Convalescent plasma	Plasma	N

6.1 Collection of convalescent plasma

Donor eligibility
Convalescent plasma donors should be recruited in a population of patients that were infected with COVID-19 and recovered. Potential donors must at least fulfill national legal requirements for eligibility of donors to donate blood or plasma.
The collection of blood products is defined by the Belgian Blood legislation (Law July 5 th 1994, and R.D. April 4 th 1996). The plasma used within this trial is collected according to the Belgian legislation.
Potential donors can be identified through collaboration with treating hospitals or practitioners or via direct search for patients cured from COVID-19.
Personal data sharing strategies must comply with national and EU data protection rules.

The following criteria for donor eligibility should be applied additional to national legal requirements:

1. an asymptomatic donor with prior diagnosis of COVID-19 documented by a laboratory test, a clinical picture with radiological confirmation of COVID-19 disease.
2. at least 28 days should have passed since full recovery and disappearance of the symptoms. Blood establishments might modify the timing of plasma collection when findings regarding the timing of optimal and maximal antibody production in those who have recovered from COVID-19 become available and is confirmed acceptable by AFMPS/FAGG.
3. Donors without a history of blood transfusion or without history of tissue or organ transplantation.
4. Donors without positive screening for irregular antibodies.
5. Female donors can only be recruited if either no history of pregnancy or if tested and found negative for anti-HLA/HPA/HNA antibodies using a validated assay. Standard donor criteria for blood or plasma donation must be met.
6. Informed consent in accordance with the Belgian Blood Legislation and national and EU data protection rules.

Plasma collection:
Collection, processing and storage
Donors will ideally donate plasma by plasmapheresis, but where apheresis is not sufficient to supply enough plasma, whole blood can also be collected, with plasma separation in the blood establishment. Eligible plasmapheresis donors are allowed, according to Belgian Blood legislation, to donate a total volume per year of 650 ml per session, 2 liter per month and 15 liter per year. The interdonation interval is at least one week. Plasma obtained by plasmapheresis and processed should be split before freezing into 2-3 separate units (e.g. 3x200 ml). Final products should be specifically labelled as COVID-19 Convalescent

Plasma and stored in a dedicated location. The processing that is routinely used for pathogen reduction by Methylene Blue will be applied according to standard practice in the blood establishment.

The production process and product characteristics will comply with current Belgian Blood legislation. For this trial, volumes will be between 200 and 250 ml. The product can be stored for 36 months at a temperature below- 25°C or for 3 months at a temperature between -18°C and -25°C.

Data collected from donors

The following data should be collected:

- date of nasopharyngeal swab that subsequently proved COVID-19-positive by PCR (or alternative date of radiological confirmation)

- Time from diagnosis until resolution of symptoms

Testing of donated plasma

Defined SARS-CoV-2 neutralizing antibody titers will be measured in the donated plasma. A Blood or Plasma sample from all donors will be stored in tube with gel for that purpose and for testing of antibody specificities.

Blood establishments will determine SARS-CoV-2 neutralizing antibody titers at or prior to the first donation to allow the selection of donors with high titers. The neutralizing antibody titers will be repeated with a maximal interval of one month. Samples of donations done in-between will be stored for later testing.

The Blood Establishment will qualify donations from donors with neutralizing antibody titers greater or equal to 1/320 as appropriate for this study.

If an adequate correlation between neutralizing activity and Elisa antibody testing for SARS-CoV2 were to be demonstrated, this assay could replace the test for neutralizing antibodies. Additional archive samples of the donated plasma will be saved for reference studies e.g. frozen aliquots from plasma samples taken at the time of donation. In emergency cases, plasma from convalescent donors might be released for transfusion without any antibody testing, then archived samples should be tested at a later date once testing is available.

When the measured neutralizing activity in the collected plasma is considered to be too low, the plasma should be made available for other use.

6.2 Convalescent Plasma and Dosing Regimen

The study design is adaptive, to allow the adjustment of a treatment arm/stratum, the addition of a new treatment arm/stratum or the removal of a treatment arm/stratum based on the most updated information in a rapidly evolving field, based on the continuous assessment of the existing evidence available for the Blood Product and other potential drug candidates.

The DAWN-PLASMA will randomize participants 2:1 to standard of care in combination with the convalescent plasma to standard of care alone. Convalescent plasma will be administered to hospitalized patients.

Patients will get a total of 800 -1000 mL plasma divided in 4 doses. A single unit has a volume that ranges from 200mL to 250mL.The first dose will be administered within 12 hours of randomization, with the absolute goal to have the first infusion of 2 units of convalescent plasma as early as practically possible after randomization. The administration of the second dose will occur between 24h and 36h after the first convalescent plasma infusion. Importantly, not the usual 30 to 45 minutes infusion time of plasma will be used but convalescent plasma should be given at the maximal speed of 50 – 100 millilitres per hour. This will reduce the risk of volume overload significantly. Determination of ABO compatibility, duration of administration should follow local SOP and plasma should be transfused within six hour after thawing. Plasma will be provided by the local blood bank. Convalescent plasma is stored in a limited number of places. Products can only be shipped to the local site only when the patient is randomized.

6.3 Concomitant / Prohibited Medication / Treatment

There are currently no approved treatments for COVID-19. Patients will receive the standard of care as continuously updated by national and international guidance.

7 Safety

7.1 Specification, timing and recording of safety parameters

- Grade 4 adverse events (life-threathening or urgent intervenstion required)
- SAEs.
- Transfusion related reactions. Transfusion of plasma is procedure with a long-standing experience. The only safety concern with convalescent plasma during acute hospital are transfusion related side effects like transfusion related acute lung injury, serious allergic transfusion reactions and transfusion associated circulatory overload.
  Patients with a documented grade 3 allergic reaction to fresh frozen plasma will be excluded from this trial.

7.1.1 Definitions

Adverse Event (AE)

An AE is any untoward medical occurrence in a patient or subject during an experiment, and which does not necessarily have a causal relationship with this treatment.
An AE can therefore be any unfavourable and unintended sign (including an abnormal laboratory finding), symptom or disease temporally associated with the use of a product, whether or not considered related to the product. Any worsening (i.e., any clinically significant adverse change in the frequency or intensity of a pre-existing condition) should be considered an AE.

Adverse Reaction (AR) or Adverse Drug Reaction (ADR)

An AR is any untoward and unintended responses to an investigational medicinal product or to an experiment and, when an investigational product is concerned, related to any dose administered.

Serious Adverse Event (SAE)

An SAE is untoward medical occurrence that results in any of the following:

- Death
- A life-threatening[a] experience
- In-patient hospitalisation or prolongation of existing hospitalisation
- A persistent or significant disability or incapacity
- A congenital anomaly or birth defect
- Important medical events that may be considered an SAE when - based on appropriate medical judgement - they may jeopardise the subject and may require medical or surgical intervention to prevent one of the above outcomes

[a] The term "life threatening" in the definition of SAE refers to an event in which the subject was at risk of death at the time of the event. It does not refer to an event which hypothetically might have caused death if it was more severe.

7.1.2 Adverse Events that do not require reporting

In general, the following should not be reported as AEs:

- Pre-existing conditions, including those found as a result of screening (these should be reported as medical history or concomitant illness).
- Pre-planned procedures, unless the condition for which the procedure was planned has worsened from the first trial-related activity after the subject has signed the informed consent.

The following events not to be considered as SAEs are:

- Pre-planned hospitalisations unless the condition for which the hospitalisation was planned has worsened from the first trial-related activity after the subject has signed the informed consent.
- Hospitalisation as part of a standard procedure for protocol therapy administration. However, hospitalisation or prolonged hospitalisation for a complication of therapy administration will be reported as an SAE.
- Hospitalisation or prolongation of hospitalisation for technical, practical, or social reasons, in absence of an AE.

For this trial, only Adverse Events graded as severe shall be collected, i.e. adverse events that are life-threatening and/or require an urgent intervention.

7.1.3 Recording and reporting of Adverse Events

Investigators will seek information on AEs during each patient contact. All events, whether reported by the patient or noted by trial staff, will be recorded in the patient's medical record and in the (e)CRF within a reasonable time after becoming aware. If available, the diagnosis should be reported on the AE form, rather than the individual signs or symptoms. If no diagnosis is available, the Investigator should record each sign and symptom as individual AEs.

The following minimum information should be recorded for each AE:

- AE description
- start and stop date of the AE
- severity
- seriousness
- causality assessment to the Blood product and/or study procedures
- outcome

As stated, selected (S)AEs will be reported. In a large outbreak like the COVID-19 pandemic the registration of all AEs is not feasible.

7.1.4 Assessment

All AEs must be evaluated by an Investigator as to:

- **Seriousness:** whether the AE is an SAE. See above for the seriousness criteria.
- **Severity:**
  - Severity must be evaluated by an Investigator according to the following definitions:
    - *Mild* – no or transient symptoms, no interference with the subject's daily activities
    - *Moderate* – marked symptoms, moderate interference with the subject's daily activities
    - *Severe* – considerable interference with the subject's daily activities, unacceptable
- **Causality:**
  - *None* – An AE which is not related to the Blood product or experiment
  - *Unlikely* – An AE for which an alternative explanation is more likely (e.g. concomitant medication(s), concomitant disease(s)), and/or the relationship in time suggests that a causal relationship is unlikely
  - *Possible* – An AE which might be due to the use of the Blood Product or the experiment. An alternative explanation is inconclusive. The relationship in time is reasonable; therefore the causal relationship cannot be ruled out.
  - *Probable* - An AE which might be due to the use of the Blood Product or the experiment. The relationship in time is suggestive (e.g. confirmed by dechallenge). An alternative explanation is less likely.
  - *Definitely* – An AE which is listed as a possible adverse reaction and cannot be reasonably explained by an alternative explanation. The relationship in time is very suggestive (e.g. it is confirmed by dechallenge and rechallenge).

7.1.5 Timelines for reporting

For this trial, only Adverse Events grade 4 shall be collected, i.e. adverse event that are life-threatening and/or require an urgent intervention. Adverse events that are also collected as outcomes will not be separately reported.

All SAEs must be reported to the Sponsor within 24 hours of the trial staff becoming aware of the event. The immediate report shall be followed by detailed, written reports. The immediate and follow-up reports shall identify subjects by Trial identification.

SAE details will be reported by the Investigator to the Sponsor:

- By completing the SAE form in the (e)CRF

7.1.6 Follow-up

The Investigator must record follow-up information by updating the patient's medical records and the appropriate forms in the (e)CRF. The worst-case severity and seriousness of an event must be kept throughout the trial.

SAE follow-up information should only include new (e.g. corrections or additional) information and must be reported within 24 hours of the Investigator's first knowledge of the information. This is also the case for previously non-serious AEs which subsequently become SAEs.

- All *SAEs* must be followed up until the outcome of the event is 'recovered', 'recovered with sequelae', 'not recovered' (in case of death due to another cause) or 'death' (due to the SAE) and until all related queries have been resolved, or until end of trial (whichever occurs first).
- *Non-serious AEs* must be followed up until the patient's last study visit, and until all related queries have been resolved.

SAEs after the end of the trial: If the Investigator becomes aware of an SAE with suspected causal relationship to the Blood Product or experiment after the subject has ended the trial, the Investigator should report this SAE within the same timelines as for SAEs during the trial.

7.1.7 Death

All deaths will be reported without delay to the sponsor (irrespective of whether the death is related to disease progression, the Blood Product, study procedure or is an unrelated event). The sponsor will notify all deaths, as soon as possible after becoming aware, to the Central EC and the EC of the concerned site and provide additional information if requested.

7.1.8 Reporting requirements to Ethics Committee's (EC's) and Competent Authorities (CA's)

The Investigator is responsible for ensuring that all safety events are recorded in the (e)CRF and reported to the Sponsor in accordance with instructions provided below.

The Sponsor will promptly evaluate all SAEs against medical experience to identify and expeditiously communicate possible new safety findings to Investigators, EC's and applicable CA's based on applicable legislation.

7.1.9 Hemovigilance

Any transfusion-related reactions in the study, must be reported to the Compentent Authorities according to the hemovigilance guidelines and local hospital procedures.

7.1.10 Annual reporting

The Sponsor has the obligation to, once a year throughout the clinical trial (or on request), submit a progress report to the EC's and CA's containing an overview of all SARs occurred during the reporting period and taking into account all new available safety information received during the reporting period.

7.1.11 Data and safety monitoring committee (DSMB) and treatment stopping rules

Data monitoring committee will composed of specialists in transfusion medicine, immunology, infectiology and a statistician. The members are independent from the Sponsor.

Given the severity of illness in COVID-19, there are no pre-specified study stopping rules for safety. The protocol team will review AE / SAE data on an ongoing basis. If there are a concerning number of unexpected AEs, the DSMB will be asked to review safety data in an ad hoc meeting.

The DSMB will review safety data after 50 of the subjects are entered into the trial, a DSMB review will be done every 3 months and ad hoc reviews will be undertaken if there are other specific safety concerns. The study will not stop enrolment awaiting these DSMB reviews, though the DSMB may recommend temporary or permanent cessation of enrolment based on their safety reviews. There are no pre-specified statistical stopping rules.

8 Statistics and Data Analysis.

Statistical analysis will be performed in accordance with ICH E9; a detailed description of the analysis is provided in the separate Trial-specific Statistical Analysis Plan (SAP). ICH E3 and E8 will guide the structure and content of the clinical trial report. A brief summary is provided here. Details will be described in the SAP.

Primary outcome

The primary outcome is <u>the number of patients alive without mechanical ventilation</u>.

The plan is to evaluate the primary outcome on Day 15, in line with the WHO master protocol.

Secondary outcomes

The WHO outcome is based on an ordinal severity scale with 11 categories. This scale has been proposed by the WHO for COVID-19 related research and has been previously used in trials of patients with influenza. Previously reported studies and ongoing studies record the same primary outcome, which allows cross-study data pooling. For the primary analysis, we use a cumulative clinical severity score, based on the 11-category ordinal scale, for the first 15 days (or other time point, based on the blinded interim analysis as described above).

This score is calculated by adding the daily severity score (highest score for that day) for each day from day 1 to day 15, thus providing a cumulative measure of disease severity during the course of the disease. The null hypothesis being tested is that the mean cumulative clinical severity score during the first 15 days is the same for the standard of care and experimental treatment arms. This is a easy to monitor endpoint.

8.1 Sample Size Determination

Despite rapid dissemination of data from clinical case series and some early stage clinical trials, detailed information about the course of the disease is limited in this stage of the COVID-19 pandemia. The samples sizes presented here are only illustrative.

Furthermore, in the absence of treatments with a known benefit, rapid changes in standard of care are to be expected and important signs of a benefit or a harm of a treatment under investigations will require rapid reporting. Safety issues will be continuously monitored by a Data and Safety Monitoring Committee, and if at any stage evidence emerges that any one treatment arm/stratum is definitely inferior then it can be decided that the study will be discontinued. Conversely, if good evidence emerges while the trial is continuing that some other treatment(s) should also be being evaluated then it can be decided that one or more extra arms or strata will be added while the trial is in progress.

Around 20% of patients hospitalized for a SARS-CoV-2 infection are admitted to the ICU with respiratory failure. When admitted to the ICU, 80% of these patients need mechanical ventilation. With the administration of convalescent plasma as early as possible, we hope to decrease the proportion of patients who have a clinical decline and need ICU support. We assume by providing passive immunity with convalescent plasma, we are able to reduce the proportion of patients admitted to the ICU from 20% to 15%. Furthermore, we assume that when admitted to ICU the need for mechanical ventilation is reduced from 80% to 50%. With a power of 0.8, a delta of 8.5% (16% in the control group and 7.5% in the invention group), a randomization ratio of 2:1 and an alpha of 0.05, sample size estimates to detect a difference

between both groups, is 483 patients with 322 patients and 161 patients in the invervention and standard of care group, respectively (using a Pearson Chi-square test for proportion difference).

We propose a pragmatic initial sample size of 483 patients (161 control patients, 322 in the plasma intervention group).

Outcomes are in line with other studies with convalescent plasma that are being set up or just started recruitment in other countries. A huge effort is now made on European scale to combine all results of other studies studying the added value of convalescent plasma. All results will be made real-time available in an European database. This allows to make sound scientific conclusions in limited time frame. When this European database is available, an amendment will be made to the institutional review board.

8.2 Statistical Analysis

8.2.1 Population for analysis

The following analysis sets will be defined:

Full Analysis Set (FAS) and modified-ITT (mITT): The FAS will include all randomised patients according to their randomised treatment. Patients randomised to the interventional group will be excluded if they did not receive any dose of study blood product The FAS will be used for the evaluation of all efficacy endpoints. mITT will be applied as primary analysis and FAS as second analysis.

Safety Set (SS): The SS will include all patients who were randomised according to their actual treatment. Patients randomised to the interventional group who did not receive any study treatment will be included in the Standard Of Care group. The SS will be used for the evaluation of all safety parameters.

8.2.2 Statistical Analyses

8.2.2.1 General Approach

This is an open label controlled randomized trial testing a superiority hypothesis with a two-sided type I error rate of 0.05. In this explorarory study, secondary hypotheses will be tested in a non-hierachical way. These will be described according to the appropriate summary statistics (e.g., proportions for categorical data, means with 95% confidence intervals for continuous data, median for time-to-event data).

A statistical analysis plan (SAP) will be developed and filed with the study sponsor prior to database lock.

8.2.2.2 Analysis of the Primary Efficacy Endpoint

Number of patients alive without mechanical ventilation at day 15 after randomization.

The null hypothesis tested is that the proportion of patients that meet the primary endpoint is equal in the standard of care and experimental treatment arm. The alternative hypothesis is that those proportions are different. The primary analysis will be based on a Cochran–Mantel–Haenszel test, stratified for centre. As a secondary analysis a logistic mixed model will be used with random centre

effect and random centre by treatment interaction, allowing to study how the treatment effects vary between centres.

8.2.2.3 Analysis of the Secondary Endpoint(s)

For the analysis of the WHO outcome, used as a sensitivity analysis, we use a cumulative clinical severity score, based on the 10-category ordinal scale, for the first 15 days (or other time point, based on the blinded interim analysis as described above). This score is calculated by adding the daily severity score (highest score for that day) for each day from day 1 to day 15, thus providing a cumulative measure of disease severity during the course of the disease. Appropriate methods will be used to account for patients for whom the status is not known for the full 15 days.

The null hypothesis being tested is that the mean cumulative clinical severity score during the first 15 days is the same for the standard of care and experimental treatment arms/strata. Because means of summed scores over a number of days are expected to be symmetrically distributed, we will use a t-test to compare the mean cumulative clinical severity score on day 15 between the treatment and the standard of care group. The treatment effect will be estimated by the difference in mean scores between the treatment groups.

As a co-WHO outcome, according to the WHO suggestions, we will use the ordinal scale to estimate a proportional odds model. For this model, the primary hypothesis test will be based on a test of whether the common odds ratio for treatment is equal to one. For large sample sizes, the hypothesis test is nearly the same as the Wilcoxon rank sum test.

Therefore, the procedure produces a valid p-value regardless of whether the proportional odds model is correct. Nonetheless, estimation and confidence intervals do require the model to be correct. Accordingly, we will evaluate model fit using a goodness-of-fit likelihood ratio test. A stratified hypothesis test to account for baseline severity of disease will be used. The treatment effect will be estimated by the common odds ratio obtained from the proportional odds logistic regression model.

1. All-Cause mortality rates will be estimated by treatment group using the Kaplan-Meier method. The resulting Kaplan-Meier curves will be compared using a log-rank test. The treatment effect will be estimated by the hazard ratio using a Cox regression.
2. Time-to-event parameters with competing risk (time to clinical improvement, composite cardiac endpoint): event rates will be estimated using cumulative incidence functions (CIF), the resulting CIF curves will be compared using Gray's test. The treatment effect will be estimated by the subdistribution hazard ratio.
3. Duration of hospital and ICU stay: both parameters will be analysed as time-to-event parameters with competing risk, whereby the event of interest is discharge from hospital/ICU and the competing risk is hospital/ICU death.
4. Continuous normally distributed variables (e.g. QTc) will be analysed using a 2-sample t-test. Treatment effects will be estimated by the difference in mean values between the groups. If applicable, changes from baseline will be calculated. Comparisons between treatment groups will be done by performing an analysis of covariance (ANCOVA) on the post-baseline value, using the baseline value as a covariate.
5. Continuous non-normally distributed variables (clinical status, NEWS score, duration of supplemental oxygen, duration of mechanical ventilation) will be analysed using a Wilcoxon rank-sum test. Change in ordinal scale at specific time points will be compared using Wilcoxon rank-sum tests.

Missing data procedures will be described in the SAP.

8.2.2.4 Safety Analyses

Safety endpoints are described above. These events will be analysed univariately and as a composite endpoint. Time-to-event methods will be used for death and the composite endpoint. Each AE will be counted once for a given participant and graded by severity and relationship to COVID-19 or study intervention.

Adverse events leading to premature discontinuation from the study intervention and serious treatment-emergent AEs will be described as part of the primary publication of the study results.

8.2.2.5 Baseline Descriptive Statistics

Baseline characteristics will be summarized by treatment arm. For continuous measures the mean and standard deviation will be summarized. Categorical variables will be described by the proportion in each category (with the corresponding sample size numbers).

8.2.2.6 Planned Interim and Early Analyses

Early analysis

Early analyses include monitoring enrolment, baseline characteristics, and follow-up rates throughout the course of the study by the study team. Analyses will be conducted blinded to treatment assignment by the DSMB.

Interim analyses

No formal interim analysis will be planned. A data and safety monitoring committee (DSMB) will monitor ongoing results to ensure patient well-being and safety as well as study integrity. The DSMB, containing one statistician, will have the opportunity to ask for data, to require analyses or to compare Belgian trial results with results available from other countries. The DSMB will be asked to recommend early termination or modification only when there is clear and substantial evidence of a safety issue.

8.2.2.7 Sub-Group Analyses

Subgroup analyses for the primary and selected secondary outcomes will evaluate the treatment effect across the following subgroups: duration of symptoms prior to enrolment, age groups, disease severity at baseline, presence of COVID-19 antibodies prior to convalescent plasma administration and co-morbidities. A forest plot will display confidence intervals across subgroups. Interaction tests will be conducted to determine whether the effect of treatment varies by subgroup.

8.3 Data & Safety Monitoring Board (DSMB)

The DSMB, composed of different members compared to the steering committee of the trial, will review safety data after 50 of the subjects are entered into the trial, a DSMB review will be done every 3 months and ad hoc reviews will be undertaken if there are other specific safety concerns. The study will not stop enrolment awaiting these DSMB reviews, though the DSMB may recommend temporary or permanent cessation of enrolment based on their safety reviews. There are no pre-specified statistical stopping rules.

Given the severity of illness in COVID-19, there are no pre-specified study stopping rules for safety. The protocol team will review AE / SAE data on an ongoing basis. If there are a concerning number of unexpected AEs, the DSMB will be asked to review safety data in an ad hoc meeting.

9 Data handling

9.1 Data Collection Tools and Source Document Identification

Data collection, handling, processing and transfer for the purpose of this Trial will be performed in compliance with applicable regulations, guidelines for clinical trials and internal procedures, as follows:

9.1.1.1 Data collection

Source Data will be collected and recorded in the Trial participant's files/medical records.

Worksheets may be used for capturing some specific data in order to facilitate completion of the eCRF. Any such worksheets will become part of the Trial participant's source documentation and will be filed together with or as part of the medical records (during but also following completion of the Trial).

It remains the responsibility of the Investigator to check that all data relating to the Trial, as specified in the Trial protocol, are entered into the eCRF in accordance with the instructions provided and that the forms are filled out accurately, completely and in a timely manner.

eCRFs are provided by the Sponsor for each participant. The Trial data will be transcribed from the source records (i.e. participant's medical file or Trial-specific source data worksheets) into an eCRF by Trial Staff. Transcription to the eCRF will be done as soon as possible after a participant visit and in a pseudonymized manner using a unique identifier assigned by the Sponsor.

The eCRFs will be available for review at the next scheduled monitoring visit (as applicable).

9.1.1.2 Data Validation

All data relating to the Trial must be prepared and validated by the Investigator. Any eCRF entries, corrections and alterations must be made by the Investigator or other authorized Trial staff.

Proper audit trails are available in REDCap to demonstrate the validity of the Trial data collected. This includes historical records of original data entries, by whom and when the data was entered, as well as detailed records of any corrections or additions made to the original data entry (i.e. who made the correction/addition, when and why), without obliterating the original data entry information.

9.1.1.3 Data Management

The Trial Data Manager will perform extensive consistency checks on the received data. Queries will be issued in case of inconsistencies in accordance with internal procedures. A Data Management Plan will be developed to map data flows, data validation measures that will be taken, how (interim) database lock(s) will be managed and, as applicable, the role and responsibilities of the Data Safety Monitoring Committee (DSMB)

9.1.1.4 Data Transfer

Any participant records or datasets that are transferred to the Sponsor or any partners of the Sponsor will contain the Trial-specific participant identifier only; participant names or any information which would make the participant identifiable will not be transferred. All pseudonymized data relating to the Trial must be transmitted in a secure manner to the Sponsor (see 9.1.2. legal requirements).

9.1.2 Legal requirements

All source data will be kept at a secured location with restricted access at all times. These data must be collected and processed with adequate precautions to ensure confidentiality and compliance with applicable data protection laws and regulations and more in particular the EU General Data Protection Regulation 2016/679 (GDPR) and relevant national laws implementing the GDPR. Appropriate technical and

organizational measures to protect the data against unauthorized disclosure or access, accidental or unlawful destruction, or accidental loss or alteration must be established. Trial staff whose responsibilities require access to personal data agree to keep the data confidential.

The Investigator and the Participating Site(s) (as applicable) shall treat all information and data relating to the Trial disclosed to them as confidential and shall not disclose such information to any third parties or use such information for any purpose other than the objectives of the Trial as described in this protocol. The collection, processing and disclosure of personal data, such as participant health and medical information is subject to compliance with applicable laws and regulations regarding personal data protection and the processing of personal data.

The Investigator will maintain all source documents and completed eCRF that support the data collected from each Trial participant, and will maintain a Trial Master File (TMF) containing all Trial documents as specified in ICH-GCP E6(R2) Chapter 8 entitled "Essential Documents for the Conduct of a Clinical Trial", and as specified by applicable regulatory requirement(s).
The Investigator will take appropriate measures to prevent accidental or premature destruction of these documents.

Transfer of the pseudonymized data will be performed via a secured method of transfer taking into account all applicable security arrangements and regulations (such as the European General Data Protection Regulation). The receiving party will be bound to keep the transferred data confidential at all times and to only process the data for the purpose of the Trial. To this end, Appendix 1 will apply.

9.2 Audits and Inspections

The Investigator will permit direct access to Trial data and documents for the purpose of monitoring, audits and/or inspections by authorized entities such as but not limited to: the Sponsor or its designees and competent regulatory or health authorities. As such eCRFs, source records and other Trial related documentation (e.g. the Trial Master File, pharmacy records, etc.) must be kept current, complete and accurate at all times.

9.3 Monitoring

In accordance with ICH-GCP E6(R2) the Sponsor is responsible for monitoring the Trial to ensure compliance with GCP and current legislation, and to verify, among other requirements, that proper written informed consent has been obtained and documented, that the Trial procedures have been followed as shown in the approved protocol, and that relevant Trial data have been collected and reported in a manner that assures data integrity. To this end Source Data will be compared with the data recorded in the eCRF. Monitoring of the Trial will be performed by qualified individuals (independent from the site Trial staff) according to the monitoring plan. The Sponsor and Investigator/Participating Site will permit direct access to the Trial data and corresponding Source Data and to any other Trial related documents or materials to verify the accuracy and completeness of the data collected. More details about the monitoring strategy are described in the Trial specific Monitoring Plan (MP).

9.4 Archiving

As specified in ICH-GCP E6(R2) section 8.1 Addendum the Sponsor and Investigator/Participating Site will maintain a record of the location(s) of all respective Essential Trial Documents (including but not limited to Source Documents, completed and final eCRF and ISF(s)/TMF). The Sponsor should ensure that the Investigator has control of and continuous access to the eCRF data reported to the Sponsor during the Trial.

The Investigator/Participating Site should have control of all Essential Documents and records generated by the Investigator/Participating Site before, during and following termination of the Trial.

The Sponsor is responsible for archiving Trial specific documentation (such as but not limited to the Trial protocol, any amendments thereto, the final Clinical Study Report (CSR) and the Trial database) according to ICH-GCP E6(R2). Source data and site-specific Trial documents (such as but not limited to the original signed ICFs) will be archived by the participating site(s) according to local practice, and for at least 25 years following termination of the Trial. Archived data may be held on electronic record, provided that media back-up exists, hard copies can be obtained, if required and measures are taken to prevent accidental or

premature loss or destruction of data. Destruction of Essential Documents will require written authorisation from the Sponsor.

10 Ethical and Regulatory Considerations

10.1 Ethics Committee (EC) review & reports

Before the start of the Trial, this protocol and other related documents (e.g. ICF, advertisements, IB, etc.) will be submitted for review to the EC and to the relevant CA for Trial authorization. The Trial shall not commence until such approvals have been obtained.

It is the responsibility of the CI to produce the Annual Progress Report (APR) and submit to the EC/CA within 30 days of the anniversary date on which favourable opinion to start the Trial was given, and annually until the Trial is declared ended.

The CI shall notify the EC/CA of the end of the Trial. Should the Trial be ended prematurely, the CI will notify the EC/CA and include the reasons for premature termination within 15 days of the decision. The CI will submit a final report with the results, including any publications/abstracts, to the EC/CA within 1 year or within 6 months for paediatric Trials.

10.2 Regulatory Compliance

The Trial will be conducted in compliance with the principles outlined in the requirements for the conduct of clinical Trials in the EU as provided for in Directive 2001/20/EC or EU Regulation 536/2014, as applicable, and any subsequent amendments, as well as in compliance with ICH-GCP E6(R2) guidelines, other GxP guidelines, the most recent version of the Declaration of Helsinki, the Belgian law of May 7th 2004 regarding experiments on the human person (as amended) or the Belgian law of May 7th 2017 on clinical Trials with medicinal products for human use, as applicable, and with the EU General Data Protection Regulation 2016/679 (GDPR), the relevant Belgian laws implementing the GDPR, the Belgian Law of August 22nd 2002 on patient rights and all other applicable legal and regulatory requirements.

10.3 Protocol / GCP compliance

The Trial must be performed in accordance with the protocol, current ICH-GCP guidelines, and applicable regulatory and country-specific requirements. GCP is an international ethical and scientific quality standard for designing, conducting, recording, and reporting studies that involve the participation of human participants. Compliance with this standard provides public assurance that the rights, safety, and well-being of Trial participants are protected, consistent with the principles that originated in the most recent version of the Declaration of Helsinki, and that the Trial data are credible, reliable and reproducible.

The Investigator and Trial team acknowledge and agree that prospective, planned deviations or waivers to the protocol are not permitted under applicable regulations on clinical studies. However, should there be an accidental protocol deviation, such deviation shall be adequately documented in the source documents and on the relevant forms and reported to the CI and Sponsor. Deviations should also be reported to the EC as part of the EC's continued review of the Trial (e.g. through the ASR, APR, etc.). Protocol deviations which are found to frequently recur, will require (immediate) action. Investigator acknowledges that such recurring protocol deviations could potentially be classified as a serious violation.

It is understood that "a serious violation" is likely to affect to a significant degree:

- the safety or physical or mental integrity of the Trial participants; or
- the scientific validity of the Trial

The Investigator is expected to take any immediate action required to protect the safety of any participant included in the Trial, even if this action represents a deviation from the protocol. In such cases, the Sponsor should be notified of this action and the EC at the Trial site should be informed according to local procedures and regulations.

10.4 Data protection and participant confidentiality

The Trial will be conducted in compliance with the requirements of the EU General Data Protection Regulation 2016/679 (GDPR), the relevant Belgian laws implementing the GDPR including the Belgian

Privacy Act of 30 July 2018 on the protection of privacy in relation to the processing of personal data. Any collection, processing and disclosure of personal data, such as participant health and medical information is subject to compliance with the aforementioned personal data protection laws (cfr. Data Processing Annex (DPA) in Appendix I). In case personal data is transferred outside the European Economic Area, safeguards will be taken to ensure that appropriate protection travels with the data in accordance with the GDPR. (https://ec.europa.eu/info/law/law-topic/data-protection/international-dimension-data-protection/rules-international-data-transfers_en#documents)

Any personal data shall be treated as confidential at all times including during collection, handling and use or processing, and the personal data (including in any electronic format) shall be stored securely at all times and with all technical and organizational security measures that would be necessary for compliance with EU and national data protection legislation (whichever is more stringent). The Sponsor shall take appropriate measures to ensure the security of all personal data and guard against unauthorized access thereto or disclosure thereof or loss or destruction while in its custody.

10.5 Insurance

The Participating Site, the Investigator and Sponsor shall have and maintain in full force and effect during the term of this Trial, and for a reasonable period following termination of the Trial, adequate insurance coverage for: (i) medical professional and/or medical malpractice liability, and (ii) general liability.

For Belgian Participating Sites

Art 29 of the Belgian Law relating to experiments on human persons dated May 7th, 2004 applies.
Prior to the start of the Trial, the Sponsor shall enter into an insurance contract in order to adequately cover Trial participants from Belgian sites in accordance with art. 29 of the said law.

For non-Belgian Participating Sites

The Participating Site shall have and maintain in full force and effect during the term of this Trial (and for a reasonable period following termination of the Trial, adequate insurance coverage for other possible damages resulting from the Trial at the Participating Site, as required by local law. Each such insurance coverage shall be in amounts appropriate to the conduct of the services of the Participating Site under this Trial. The Participating Site and Sponsor shall be solely responsible for any deductible or self-insured retention under any such policies.

10.6 Access to the Study Data by KCE and similar institutes in the EU

This section should be read in conjunction with the research agreement for funding, which supersedes the protocol in case of contradictory statements.

After the completion of the study the Sponsor will transfer the pseudonymized study data set to KCE. KCE will request approval from the competent chamber of the Information Security Committee to have the relevant study data linked with IMA data by a trusted third party (TTP, eHealth platform) using the patient national number.

The patient information and consent includes wording that the national number will be recorded on site by the investigator for later data linkage. The patient information and consent will also include that in case the patient is randomized, it is planned that a trusted third party (TTP, eHealth platform) will receive and use the national number to link with IMA administrative data. This data linkage is planned to obtain a more complete data set that will be used for the analysis of effectiveness and cost-effectiveness of the intervention by KCE.

KCE and Sponsor have entered into a research agreement detailing the roles and responsibilities of each party, as well as other legal aspects of this collaboration, including the right to use and access of KCE to the Study Data.

“Background” means any intellectual property (IP), data, materials, information owned or controlled by the Sponsor or a Site, and required to run this Study. Sponsor will identify such Background including the legal restrictions of which Sponsor or Sites are aware that may affect the use of the Background for the purpose of the Study or the rights granted to KCE under this Agreement.

The Study Data consist of this protocol, including amendments, the electronic forms for data capture, including the annotations and guidance for use, the electronic database of the pseudonymized clinical and non-clinical data collected using data capture, including the log of changes from data entry to database lock, study reports based on these pseudonymized data, and any data or reports generated at a later stage, eg based on exploratory analyses or stored samples.

“Foreground” means any Study Data, and any tangible biological, chemical and physical material and inventions, that are generated, acquired, discovered, conceived, developed, created, exemplified or derived as a result of carrying out the Clinical Study, whatever its form or nature, whether it can be protected or not, as well as any Foreground IP. Sponsor acknowledges that the main purpose of the research performed under this Agreement is to generate results that will serve the general public interests, and specifically the interests of the patients and public healthcare decision making bodies, and, therefore, undertakes not to exploit the Foreground in any way that is or could be detrimental to such interests.

The Sponsor owns the Study Data, but provides KCE with a copy of the pseudonymized database after database lock as well as a royalty-free unrestricted license to use the Study Data for non-commercial public health related purposes as detailed in the Agreement between KCE and UZ Gent. If judged appropriate, KCE will introduce the request to the competent chamber of the Information Security Committee and arrange for the data linkage. For the sake of clarity, the linked data are not part of the Study Data. However, KCE will discuss with the Sponsor the results of the analyses and the reporting of the linked data.

Access to Study Data by KCE and similar institutes in the EU is fully defined in the contract between KCE and the Sponsor. The research agreement template is publicly available on the KCE website. Link:
https://kce.fgov.be/en/resources-for-investigators

10.7 Amendments

Unless for urgent reasons as specified in ICH-GCP E6(R2) section 4.5.4, amendments must not be implemented prior to EC and/or CA review and/or approval, as applicable.
In accordance with the Belgian law of May 7th 2004 regarding experiments on humans, the Sponsor may develop a non-substantial amendment at any time during the Trial. If a substantial amendment to the clinical Trial agreement or the documents that supported the original application for the clinical Trial authorisation is needed, the Sponsor must submit a valid substantial amendment to the Competent Authority (CA) for consideration, and to the EC for review and approval. The CA and/or EC will provide a response in accordance with timelines defined by applicable regulations. It is the Sponsor’s responsibility to assess whether an amendment is substantial or non-substantial for the purpose of submission to the CA and/or EC.

Amendments to the Trial are regarded as ‘substantial’ when they are likely to have a significant impact on the safety or physical or mental integrity of the clinical Trial participants, or the scientific value of the Trial.

https://ec.europa.eu/health/sites/health/files/files/eudralex/vol-10/2010_c82_01/2010_c82_01_en.pdf

10.8 Post-Trial activities

Not applicable.

11 Research Registration, Dissemination of Results and Publication Policy

The Declaration of Helsinki (latest version) and European and Belgian regulations require that every research Trial involving human participants be registered in a publicly accessible database before recruitment of the first participant. The CI is responsible for registering the Trial.

In addition, the CI will fulfil their ethical obligation to disseminate and make the research results publicly available. As such the CI is accountable for the timeliness, completeness and accuracy of the reports. Researchers, authors, Sponsors, editors and publishers must adhere to accepted guidelines for ethical reporting. Negative and inconclusive, as well as positive results must be published or otherwise made publicly available. Sources of funding, institutional affiliations and conflicts of interest must be declared in publication.

Publications will be coordinated by the CI. Authorship to publications will be determined in accordance with the requirements published by the International Committee of Medical Journal Editors and in accordance with the requirements of the respective medical journal.

For multi-centric Trials, it is anticipated that the primary results of the overall Trial shall be published in a multi-centre publication.

Participating Sites are not allowed to publish any subset data or results from the Trial prior to such multicentre publication.

Any publication by a Participating Site must be submitted to the Sponsor for review at least thirty (30) calendar days prior to submission or disclosure. Sponsor shall have the right to delay the projected publication for a period of up to three (3) months from the date of first submission to the Sponsor in order to enable the Sponsor to take steps to protect its intellectual property rights and know-how.

12 Intellectual Property

Any know-how, inventions, methods, developments, innovations, discoveries and therapies, whether patentable or not, arising from the Trial or made in the performance of the Trial protocol ("Inventions") shall vest in the Sponsor. The Participating Site, its employees and Investigator(s) shall promptly disclose to the Sponsor any such Inventions. Parties have expressly agreed that any and all Trial data as collected and prepared in the performance of the Trial protocol shall be the sole property of Sponsor. Publication policy guidelines will be created.

13 Joint Commission International (JCI)

In order to ensure the same quality and safety standards in patient care for clinical research as commonly applied by the Sponsor in its regular activities, and in accordance with JCI standards, the Sponsor shall comply with the following obligations: (a) the Sponsor will use trained and qualified employees or contractors to manage and coordinate the Trial; (b) the Sponsor will ensure that multi-center Trial reporting is reliable and valid, statistically accurate, ethical, and unbiased. (c) the Sponsor will not grant incentives, other than standard compensations and reimbursement of costs, to Trial participants or to participating site's staff that would compromise the integrity of the research; (d) the Sponsor is responsible for monitoring and evaluating the quality, safety, and ethics of the Trial and will respect the participating site's policies and processes when performing such monitoring and evaluation activities; (e) the Sponsor will protect the privacy and confidentiality of the Trial participants in accordance with all applicable laws.

References

1. Zhou F, Yu T, Du R, et al. Clinical course and risk factors for mortality of adult inpatients with COVID-19 in Wuhan, China: a retrospective cohort study. *Lancet*. 2020;395(10229):1054-1062. doi:10.1016/s0140-6736(20)30566-3
2. Keyaerts E, Vijgen L, Maes P, Neyts J, Ranst M Van. In vitro inhibition of severe acute respiratory syndrome coronavirus by chloroquine. *Biochem Biophys Res Commun*. 2004;323(1):264-268. doi:10.1016/j.bbrc.2004.08.085
3. Mehta P, Mcauley DF, Brown M, et al. Correspondence COVID-19 : consider cytokine storm syndromes and. *Lancet*. 2020;6736(20):19-20. doi:10.1016/S0140-6736(20)30628-0
4. Tang N, Li D, Wang X, Sun Z. Abnormal coagulation parameters are associated with poor prognosis in patients with novel coronavirus pneumonia. *J Thromb Haemost*. 2020;(February):844-847. doi:10.1111/jth.14768
5. Tang N, Bai H, Chen X, Gong J, Li D, Sun Z. Anticoagulant treatment is associated with decreased mortality in severe coronavirus disease 2019 patients with coagulopathy. *J Thromb Haemost*. 2020. doi:10.1111/jth.14817
6. Wu C, Liu Y, Yang Y, et al. Analysis of therapeutic targets for SARS-CoV-2 and discovery of potential drugs by computational methods. *Acta Pharm Sin B*. 2020. doi:10.1016/j.apsb.2020.02.008
7. Takano T, Akiyama M, Doki T, Hohdatsu T. Antiviral activity of itraconazole against type i feline coronavirus infection. *Vet Res*. 2019;50(1):1-6. doi:10.1186/s13567-019-0625-3
8. Schloer S, Goretzko J, Kühnl A, Brunotte L, Ludwig S, Rescher U. The clinically licensed antifungal drug itraconazole inhibits influenza virus in vitro and in vivo. *Emerg Microbes Infect*. 2019;8(1):80-93. doi:10.1080/22221751.2018.1559709
9. Parnham MJ, Haber VE, Giamarellos-Bourboulis EJ, Perletti G, Verleden GM, Vos R. Azithromycin: Mechanisms of action and their relevance for clinical applications. *Pharmacol Ther*. 2014;143(2):225-245. doi:10.1016/j.pharmthera.2014.03.003
10. Albert RK, Connett J, Bailey WC, et al. Azithromycin for Prevention of Exacerbations of COPD. *N Engl J Med*. 2011;365(8):689-698. doi:10.1056/NEJMoa1104623
11. Brusselle GG, VanderStichele C, Jordens P, et al. Azithromycin for prevention of exacerbations in severe asthma (AZISAST): A multicentre randomised double-blind placebo-controlled trial. *Thorax*. 2013;68(4):322-329. doi:10.1136/thoraxjnl-2012-202698
12. Vermeersch K, Gabrivska M, Aumann J, et al. Azithromycin during Acute Chronic Obstructive Pulmonary Disease Exacerbations Requiring Hospitalization (BACE). A Multicenter, Randomized, Double-Blind, Placebo-controlled Trial. *Am J Respir Crit Care Med*. 2019;200(7):857-868.
13. Vos R, Vanaudenaerde BM, Verleden SE, et al. A randomised controlled trial of azithromycin to prevent chronic rejection after lung transplantation. *Eur Respir J*. 2011;37(1):164-172. doi:10.1183/09031936.00068310
14. Wong C, Jayaram L, Karalus N, et al. Azithromycin for prevention of exacerbations in non-cystic fi brosis bronchiectasis (EMBRACE): A randomised, double-blind, placebo-controlled trial. *Lancet*. 2012;380(9842):660-667. doi:10.1016/S0140-6736(12)60953-2
15. Menzel M, Akbarshahi H, Bjermer L, Uller L. Azithromycin induces anti-viral effects in cultured bronchial epithelial cells from COPD patients. *Sci Rep*. 2016;6(June):1-11. doi:10.1038/srep28698
16. Gielen V, Johnston SL, Edwards MR. Azithromycin induces anti-viral responses in bronchial epithelial cells. *Eur Respir J*. 2010;36(3):646-654. doi:10.1183/09031936.00095809
17. Van Herck A, Frick AE, Schaevers V, et al. Azithromycin and early allograft function after lung transplantation: A randomized, controlled trial. *J Hear Lung Transplant*. 2019;38(3):252-259. doi:10.1016/j.healun.2018.12.006
18. Vermeersch K, Belmans A, Bogaerts K, et al. Treatment failure and hospital readmissions in severe COPD exacerbations treated with azithromycin versus placebo - A post-hoc analysis of the BACE randomized controlled trial. *Respir Res*. 2019;20(1):1-12. doi:10.1186/s12931-019-1208-6
19. Posch M, Proschan MA. Unplanned adaptations before breaking the blind. *Stat Med*. 2012;31(30):4146-4153. doi:10.1002/sim.5361
20. Mulangu S, Dodd LE, Davey RT, et al. A randomized, controlled trial of Ebola virus disease therapeutics. *N Engl J Med*. 2019;381(24):2293-2303. doi:10.1056/NEJMoa1910993
21. Cao B, Wang Y, Wen D, et al. A Trial of Lopinavir-Ritonavir in Adults Hospitalized with Severe Covid-19. *N Engl J Med*. 2020:1-13. doi:10.1056/NEJMoa2001282

Some references have been documented by PubMed ID number. The corresponding article can be easily found on https://www.ncbi.nlm.nih.gov/pubmed/ .

APPENDICES

14 Appendix 1: Data Processing Annex (DPA)

Definitions:

- "Protocol" means the document entitled "A randomized, open-label, adaptive, proof-of-concept clinical trial of convalescent plasma against SARS-CoV-2" containing the details of the academic Trial as developed by the Sponsor and approved by the relevant Ethics Committee.
- "Sponsor" means University Hospitals Leuven (UZ Leuven).
- Participating site acts as a data processor as defined under article 4, 8) of the Regulation (EU) 2016/679 ("Data Processor") for the Sponsor who acts as data controller as defined under article 4, 7) of the Regulation (EU) 2016/679 ("Data Controller").
- "Applicable Law" means any applicable data protection or privacy laws, including:
    a) the Regulation (EU) 2016/679 also referred as the General Data Protection Regulation ("GDPR");
    b) other applicable laws that are similar or equivalent to or that are intended to or implement the laws that are identified in (a) of this definition;
- "Personal Data" means any information relating to an identified or identifiable natural person ("Data Participant"), including without limitation pseudonymized information, as defined in Applicable Law and described in the Protocol.

Rights and obligations:

1. The Data Processor is instructed to process the Personal Data for the term of the Trial and only for the purposes of providing the data processing tasks set out in the Protocol. The Data Processor may not process or use Personal Data for any purpose other than a Data Participant's medical records, or other than provided in the instructions of the Trial protocol, including with regard to transfers of personal data to a third country or an international organization, unless the Data Processor is required to do so according to Union or Member State law.
2. Data Processor shall at all times maintain a record of processing of Personal Data in accordance with Applicable Law and if the Data Processor considers an instruction from the Data Controller to be in violation of the Applicable Law, the Data Processor shall promptly inform the Data Controller in writing about this.
3. The Data Processor must ensure that persons authorized to process the Personal Data have committed themselves to confidentiality or are under an appropriate statutory obligation of confidentiality.
4. The Data Processor shall implement appropriate technical and organizational measures to prevent that the Personal Data processed is:
    (i) accidentally or unlawfully destroyed, lost or altered,
    (ii) disclosed or made available without authorization, or
    (iii) otherwise processed in violation of Applicable Law.
5. The appropriate technical and organizational security measures must be determined with due regard for:
    (i) the current state of the art,
    (ii) the cost of their implementation, and
    (iii) the nature, scope, context and purposes of processing as well as the risk of varying likelihood and severity for the rights and freedoms of natural persons.

6. Taking into account the nature of the processing, the Data Processor shall assist the Data Controller, by means of appropriate technical and organizational measures, insofar as this is possible, in fulfilling its obligation to respond to requests from Data Participants pursuant to laws and regulations in the area of privacy and data protection (such as, the right of access, the right to rectification, the right to erasure, the right to restrict the processing, the right to data portability and the right to object)
7. The Data Processor shall upon request provide the Data Controller with sufficient information to enable the Data Controller to ensure that the Data Processor's obligations under this DPA are complied with, including ensuring that the appropriate technical and organizational security measures have been implemented.
8. The Data Controller is entitled to appoint at its own cost an independent expert, reasonably acceptable to the Data Processor, who shall have access to the Data Processor's data processing facilities and receive the necessary information for the sole purpose of auditing whether the Data Processor has implemented and maintained said technical and organizational security measures. The expert shall upon the Data Processor's request sign a non-disclosure agreement provided by the Data Processor, and treat all information obtained or received from the Data Processor confidentially, and may only pass on, after conferral with the Data Processor, the findings as described under 10) (ii) below to the Data Controller.
9. The Data Processor must give authorities who by Union or Member State law have a right to enter the Data Controller's or the Data Controller's processors' facilities, or representatives of the authorities, access to the Data Processor's physical facilities against proper proof of identity and mandate, during normal business hours and upon reasonable prior written notice.
10. The Data Processor must without undue delay in writing notify the Data Controller about:
    (i) any request for disclosure of Personal Data processed under the Protocol by authorities, unless expressly prohibited under Union or Member State law,
    (ii) any finding of (a) breach of security that results in accidental or unlawful destruction, loss, alteration, unauthorized disclosure of, or access to, Personal Data transmitted, stored or otherwise processed by the Data Processor under the Protocol, or (b) other failure to comply with the Data Processor's obligations, or
    (iii) any request for access to the Personal Data (with the exception of medical records for which the Data Processor is considered data controller) received directly from the Data Participants or from third parties.
11. Such a notification from the Data Processor to the Data Controller with regard to a breach of security as meant in 10) (ii)(a) above will contain at least the following information:
    (i) the nature of the Personal Data breach, stating the categories and (by approximation) the number of Data Participants concerned, and stating the categories and (by approximation) the number of the personal data registers affected (datasets);
    (ii) the likely consequences of the Personal Data breach;
    (iii) a proposal for measures to be taken to address the Personal Data breach, including (where appropriate) measures to mitigate any possible adverse effects of such breach.
12. The Data Processor shall document (and shall keep such documentation available for the Data Controller) any Personal Data breaches, including the facts related to the Personal Data breach, its effects and the corrective measures taken. After consulting with the Data Controller, the Data Processor shall take any measures needed to limit the (possible) adverse effects of Personal Data breaches (unless such consultation cannot be awaited due to the nature of the Personal Data breach).
13. The Data Processor must promptly and reasonably assist the Data Controller (with the handling of (a) responses to any breach of security as described in 10) (ii) above and (b) any requests from Data Participants under Chapter III of the GDPR, including requests for access, rectification, blocking or deletion. The Data Processor must also reasonably assist the Data Controller by

implementing appropriate technical and organizational measures for the fulfilment of the Data Controller's obligation to respond to such requests.

14. The Data Processor must reasonably assist the Data Controller with meeting the other obligations that may be incumbent on the Data Controller according to Union or Member State law where the assistance of the Data Processor is implied, and where the assistance of the Data Processor is necessary for the Data Controller to comply with its obligations. This includes, but is not limited to, at the request to provide the Data Controller with all necessary information about an incident under 10) (ii), and all necessary information for an impact assessment in accordance with Article 35 and Article 36 of the GDPR.

Subprocessor:

15. The Data Processor may only engage a subprocessor, with prior specific or general written consent from the Data Controller. The Data Processor undertakes to inform the Data Controller of any intended changes concerning the addition or replacement of a subprocessor by providing a reasonable prior written notice to the Data Controller. The Data Controller may reasonably and in a duly substantiated manner object to the use of a subprocessor. The Data Processor must inform the Data Controller in writing of the discontinued use of a subprocessor.
16. Prior to the engagement of a subprocessor, the Data Processor shall conclude a written agreement with the subprocessor, in which at least the same data protection obligations as set out in this DPA shall be imposed on the subprocessor, including obligations to implement appropriate technical and organizational measures and to ensure that the transfer of Personal Data is done in such a manner that the processing will meet the requirements of the Applicable Law.
17. The Data Controller has the right to receive a copy of the relevant provisions of Data Processor's agreement with the subprocessor related to data protection obligations. The Data Processor shall remain fully liable to the Data Controller for the performance of the subprocessor obligations under this DPA. The fact that the Data Controller has given consent to the Data Processor's use of a subprocessor is without prejudice for the Data Processor's duty to comply with this DPA.
