# Supplementary material for: A randomized, multicentre, open-label phase II proof-of-concept trial investigating the clinical efficacy and safety of the addition of convalescent plasma to the standard of care in patients hospitalized with COVID-19: the Donated Antibodies Working against nCoV (DAWn-Plasma) trial
Source: Trials. 2020 Nov 27;21:981. doi: 10.1186/s13063-020-04876-0 (PMC7691949; doi:10.1186/s13063-020-04876-0)
Supplement: Supplementary file 3 — Additional file 3. [file 13063_2020_4876_MOESM3_ESM.pdf]

A randomized, open-label, adaptive, proof-of-concept clinical trial of Donated Antibodies Working agaiNst COVID-19: DAWN-PLASMA

Study number:

Sponsor of the Trial: Universitaire Ziekenhuizen Leuven (UZ Leuven)

Site Name:

Who can I contact in case of questions?

**Name**	**Function**	**In case of**	**Contact details**
Surname, First name	Principal Investigator of the site	Information, problems or concerns	Phone N°, E-mail
	The trial staff	Information, problems, concerns	Phone N°
	Emergency contact	Emergency	Phone N°
	Patient rights ombudsman	Concerns relating to your rights as a participant in a trial	Phone N°
Name and address of insurance company of the sponsor & contact of insurer	Insurance Company of the sponsor	In case of disagreement or complaint on a damage claim	Policy N°: 299.053.700
	Data protection officer of the **site**	Questions relating to the confidentiality of your data	Phone N°<br>E-mail: e-mail
	Belgian Data Protection Authority	Complaints relating to the confidentiality of your data	E-mail : contact@apd-gba.be

Table of contents

Who can I contact in case of questions? **2**

CHAPTER I – DESCRIPTION OF THE TRIAL AND YOUR RIGHTS WHEN PARTICIPATING **5**

1. Why are we doing this trial **5**

2. Why am I being asked to take part? **6**

3. Do I have to take part in a trial? **6**

4. What will happen during the trial? **7**

5. Will I benefit from the trial? **8**

6. What are the possible risks and discomforts of taking part? **9**

6.1. What are the possible side effects of convalescent plasma? 9

6.2. Can I take other medicines during the trial? 9

6.3. Will my participation to the trial have an impact on my daily activities? 9

7. What If something goes wrong within the trial? **9**

8. What if other treatment options or new information on the IMP become available during the course of the trial? **10**

9. Can my participation in the trial end prematurely? **10**

9.1. You decide to withdraw your consent 11

9.2. The investigator decides to end your trial participation 11

9.3. Other entities may interrupt or end the trial 11

10. Which treatment will I get after my participation in the trial? **12**

11. Will my participation in the trial involve extra costs for me? **12**

11.1. Examinations and treatments paid by the sponsor 12

12. Which data are collected about me during the trial and what will happen with them? **12**

12.1. Which data are collected and processed during the trial? 12

12.2. How will the investigator treat my personal data? 12

12.3. What will happen to information about me collected during the trial? 13

12.4. How will my data be handled? 13

12.5. Do I have access to my data collected and processed during the trial and can I rectify them ? 13

12.6. Who, other than the Investigator and his staff, has access to my personal data? 14

12.7. What will happen to the results of the trial? 15

12.8. Will my data be used for other purposes than for the trial in which I take part? 15

12.9. How long will my data be kept? 16

13. Which biological samples are collected from me during the trial and what will happen with them? **16**

13.1. Which biological samples are collected from me during the trial? 16

13.2. What will happen to the collected biological samples? 16

13.3. How will my biological samples be handled? 16
13.4. What happens with any remainders of biological samples once the analyses described in this document have been carried out? 16
Will any additional biological samples be collected and used for additional research? 17
14. Who has reviewed and approved the trial documents? 17
15. What happens in case of incidental findings? 17

CHAPTER II - INFORMED CONSENT 18
PARTICIPANT 18
LEGAL REPRESENTATIVE (REF.) 20
IMPARTIAL WITNESS / INTERPRETER (REF.) 21
INVESTIGATOR 22

GLOSSARY 23

REFERENCES 24

CHAPTER I – DESCRIPTION OF THE TRIAL AND YOUR RIGHTS WHEN PARTICIPATING

1. Why are we doing this trial

Which disease, and which treatment, are being studied?

In December 2019, a new coronavirus (the SARS-CoV-2 virus) emerged, which spread fast across the world, and has led to a pandemic. The disease caused by this virus, called COVID-19, has a mild course in a high number of infected people, but is known to have a more severe course in some. Currently, there are no successful treatments with a known effect on the course of the disease in patients suffering from COVID-19. In this study, we will examine whether plasma, a treatment that has been used in human subjects for other diseases (and is known to be safe), can be used to influence the course of COVID-19 towards a lower severity.

What is plasma?

Your blood consists for 55% of plasma, a yellow fluid. Plasma consists mainly of water (91.5%), and dissolved substances (8.5%) such as proteins, sugar, fat, salt, hormones and vitamins. Some of these substances are vital for the coagulation process, and the defence against micro-organisms. In plasma of people who already recovered from COVID-19 (called convalescent plasma), antibodies against the new coronavirus can be found. These are proteins, produced by the human body, to combat infections such as COVID-19. By collecting plasma of people who already healed, and to administer them to patients who are sick, these antibodies can be transferred, and the disease process could possibly be slowed down or even halted.

Which type of study, and why?

This clinical trial (further on referred to as “trial”) is an adaptive, randomized, open-label phase II proof-of-concept study to investigate the safety and effect of potentially interesting treatments, for the treatment of hospitalized adult patients suffering from COVID-19.

We explain further:

‘adaptive’: The question which experimental treatments could potentially be applied is being examined. Therefore, this study is designed as an **adaptive** trial, which implies that during the course of the trial, additional treatment options may be added to investigate the efficacy and safety of these other treatments, yet to be identified. For each change in the protocol, your permission will be requested.

‘randomized’: The study is ‘randomized’. This means that participants are attributed to one of the treatment arms by random chance. Such a procedure is necessary: to be able to investigate whether a treatment is effective, we need to compare with standard treatment

‘open label’: The study is ‘open label’. This means that doctors and patients will know whether they have received the investigational treatment, or standard treatment.

‘phase II proof-of-concept’: This study is designed to investigate safety and efficuacy of a treatment, compared to investigational treatments, in patients with COVID-19.

Why do we investigate whether plasma administration could be effective in case of a COVID-19 infection?

In the present study, we investigate if the administration of blood-plasma from patients recovered from COVID-19, could be effective to treat patients who are severely ill because of a COVID-19 infection. The general idea behind the transfusion, is that plasma of recovered patients contains antibodies that could eliminate the novel coronavirus causing COVID-19, and lead to a less severe course of the disease, or a faster healing. Simply put, in this study we would like to investigate whether ‘borrowed immunity’ from a person who has cured from this disease, could be applied to cure other patients more rapidly.

Plasma donation has been investigated before, on a smaller scale, in infectious diseases such as the flu, or ebola. In these studies, plasma donation from patients who already had the disease, appeared to lead to a lower mortality, or a better survival. However, large studies that have demonstrated a clear effect have not been performed, because vaccines or new medications have been developed for these conditions, or because the epidemic could be stopped at an early stage. For other conditions, such as immune system disease or conditions affecting coagulation, plasma donation is often applied.

Because an active treatment for COVID-19 is lacking, plasma donation is now being proposed. In a small group of Chinese patients with COVID-19, the treatment had a possible positive effect. However, this effect has not yet been demonstrated in a clinical trial. To investigate this effect of plasma, we will compare two groups of patients. One group will get standard treatment, plus plasma from a patient recently cured from COVID-19. A second group will get standard treatment alone. In this study, 2 patients out of 3 will receive plasma, and 1 patient out of 3 will receive standard treatment alone. After the study, we will be able to establish whether plasma donation is useful or not.

2. Why am I being asked to take part?

You have been diagnosed with COVID-19.

You are being asked to take part in this trial because you have been admitted to the hospital with COVID-19.

For this condition, no alternative experimental treatments have demonstrated a clear beneficial effect on the course of COVID-19, apart from supportive care.

The investigator or trial staff will discuss with you the requirements to be allowed to enter the trial.

3. Do I have to take part in a trial?

Your participation in a trial is voluntary and must remain free of any coercion. This means that you have the right not to take part in the trial or to withdraw at any time without giving a reason, even if you previously agreed to take part. Your decision will not affect your relationship with the investigator or your treating physician nor will it affect the quality of your future medical care.

4. What will happen during the trial?

This trial will include about 480 participants. In case the treatment groups would be adapted during the trial (since this is an adaptive trial), a larger or smaller number of participants might be involved. The trial will run in this hospital, and in other hospitals as well.

4.1. What exactly does the treatment consist of?

If you are randomized (by chance) to receive plasma, you will receive, within 12 hours after randomization, 2 units of plasma, which consist of approximately 250 ml of plasma, in total half a liter. Between 24 and 36 hours after randomization, you will receive 2 additional units of 250 ml each.

Before randomization (by chance) to determine your treatment group, all participants to the study will be evaluated, to assess whether they can participate to the study. This evaluation involves laboratory tests, that are part of your clinical care. Blood samples and naso-pharyngeal swabs (swabs of the nose and throat) will be done according the the clinical need, in order not to increase the workload of healthcare workers or to exhaust the supply.

The study could run for 18 months in total, but in your specific case, it will take about 90 days, from screening on the first day, to follow up calls on day 15, day 30 (plus or minus 3 days) and day 90 (plus or minus 3 days).

Should you be eligible for participation in this trial, and should you agree to participate, you will be subjected to the tests and exams mentioned above, and summarized in the table below. In case of important side-effects, the researcher could decide to request additional tests, that will be considered study-specific.

4.2. How will this study be done exactly?

Start of the study:

At the start of the study, it will be investigated whether you are eligible for participation. We will also check the results of laboratory tests. Additional blood will be taken, to determine your blood group, and to investigate if the plasma can be administered safely.

We will explain the study and run through this text, and request permission from you whether you would agree to participate. If you are eligible, and agree to participate, you will be attributed to one of the treatment arms (standard treatment or intervention with plasma).

Daily procedures

Apart from the administration of convalescent plasma, your care, treatments and follow-up during the study will be done exactly in the same way as in patients who do not participate. You will get the same examinations and treatments, if necessary.

For this study specifically, an additional blood sample will be collected before the plasma is administered, and on day 6 (only in case you are still in the hospital on that day). This is necessary to perform certain study-specific laboratory tests.

Follow-up

Data will collected on day 15, 30 and 90 after the start of the study. Should you be discharged from the hospital already, you will be called by telephone.

Below are the interventions that will be carried out during the study schematically listed:

	Screening	Baseline						
**Evaluations/ Procedures**	**Day -1 or 1**	**Day 1**	**24-36h after 1st plasma administration**	**Daily until hospital discharge**	**Day 6 +/-2**	**Day 15 +/-2**	**Day 30 +/- 3**	**Day 90 +/- 3**
**Does the patient apply?**	X							
Informed consent	X							
Demographics and medical history	X							
Review COVID-19 criteria	X							
In- and exclusion criteria	X							
Blood typing	X							
**STUDY INTERVENTION**								
Randomisation		X						
Plasma administration		2 units within 12h after randomization	2 units within 24-36h after 1st plasma	.				
**STUDY-PROCEDURES**								
Vital signs		X		Daily until discharge				
Clinical data collection		X		Daily until discharge				
Evaluation of plasma administration		X	X					
Evaluation of side effects		X		Daily until discharge				
Questionnaire quality of life		X					Optional	X
VAS Pain Score	X			Daily until discharge				
Telephone interview if no longer hospitalized						X	X	If in person meeting is not possible
**LAB**								
CRP, blood count, chemistry, kidney function, liver tests, nasal/throat swabs	X	According to treating physician		According to treating physician				X
nasal/throat swabs	X				If feasible			
Additional test for antibody titers	X				If feasible			
Pregnancy test for women of childbearing age	X							

5. Will I benefit from the trial?

The information obtained during a trial may contribute to a better understanding of the use of convalescent plasma for the treatment of yourself or future patients.

Convalescent plasma may or may not be beneficial in treating COVID-19 or relieving your symptoms. Even if it is beneficial to you, a potential return or worsening of symptoms, illness or disease is still possible.

There is always a risk of an increase in the disease with anti-SARS-CoV-2 antibodies (ADE: Antibody-Dependent Enhancement)

6. What are the possible risks and discomforts of taking part?

It is very important to report any new or worsening health problem to the researcher. This also applies, even if you think the problem has nothing to do with the study, and even if it has been described already in this document. Should you take other medications, please discuss with the researcher before taking them. This could be very important for a correct diagnosis or treatment, if necessary. The administration of convalescent plasma will only be performed in-hospital.

6.1. What are the possible side effects of convalescent plasma?

All medicinal products can have side effects. There is a long experience with plasma therapy, and the side effects are well-known, and rare. Some patients might experience an allergic reaction, such as shivering, fever, or itching rash. This can be treated with drugs, but in rare cases a more severe allergic reaction might occur. Administration of blood products always has a low risk of transmission of infections, even with maximal safety procedures.

In this study, transfusion-related side effects will be examined carefully, in order to follow-up on this study carefully, and guarantee maximum safety for all participants.

6.2. Can I take other medicines during the trial?

Plasma will only be administered during hospitalisation, and has no influence on treatments after discharge. Do not hesitate to ask your investigator for more explanation about the use of other medicines and food supplements.

6.3. Will my participation to the trial have an impact on my daily activities?

Plasma will only be administered during hospitalisation, and has no influence on treatments after discharge.

7. What If something goes wrong within the trial?

Even if there is no fault, the sponsor is liable for harm caused to you whether directly or indirectly related to your participation in the trial. The sponsor has taken an appropriate insurance (a so called “NO FAULT INSURANCE”) for this liability (Ref. 1). A copy of the insurance certificate can be obtained from the investigator or trial staff.

If you (or in the event of death, your rightful claimants) seek compensation for a harm to your health as a direct or indirect result of participating in the trial, you must inform your investigator or trial staff promptly.

If the investigator believes that a link between the new or worsened health problem(s) and the trial is possible, he/she will inform the trial sponsor. The sponsor will then immediately initiate the declaration procedure to its insurance company. If the company considers it necessary, it will appoint an expert to assess whether there is a link between your reported health problem(s) and the trial. The insurance does not

cover the natural progression of your disease/condition or the known side effects of the treatment you would have received without taking part to the trial (*that is* your standard treatment).

Whenever you feel it is appropriate or if you or your rightful claimants disagree either with the investigator or with the expert appointed by the insurance company, you may contact the insurance company or proceedings may be brought against the insurance company. You will find the contact details on the front page of this form.

8. What if other treatment options or new information on the IMP become available during the course of the trial?

During the course of the trial, important new information might become available, possibly affecting your decision to (further) participate. For example other treatments for COVID-19 or important new information on convalescent plasma may become available. It is the duty of the investigator to discuss this new information with you and to give you the opportunity to re-consider your participation in the trial.

If you decide to stop taking part in the trial or if you are no longer able to participate, your investigator will see to it that you continue to receive the best possible medical care.

9. Can my participation in the trial end prematurely?

As explained in detail below, your trial participation may end prematurely when

- you decide to withdraw your consent,
- the investigator decides to end your trial participation, or
- other entities interrupt or end the trial.

In any case, if your trial participation ends prematurely, the investigator will discuss your future medical care with you. The sponsor can continue to retain and use any data that have already been collected before the end of your participation. This is to avoid skewing / biasing results of the trial (as described in I. § 12.4., page 13).

If you experience a side effect at the moment of stopping the IMP, the investigator may contact you in the future to see if it has resolved or not after the end of the trial participation.

If you experience a new side effect after the end of your trial participation you may contact the investigator to ask for a follow-up.

Because COVID-19 is a serious condition, no pre-defined stopping rules for safety have been mentioned in the protocol. The Data Safety Monitoring Board (DSMB) will evaluate safety issues and can assess security data or establish when other specific safety problems would occur. Based on their judgment of safety, the DSMB can advise to temporarily interrupt, or permanently stop the trial.

9.1. You decide to withdraw your consent

You are entitled to withdraw your consent for any reason, at any time, without having to justify your decision. However, for your safety, you should inform the investigator of your decision. Although it is not mandatory, it may be useful for the investigator and for the sponsor to know the reason of your decision (for example side effects, frequency of clinical visits,…).

If you withdraw your consent, this means you decide to stop

- the treatment with the Study Intervention, and
- all trial-related visits and examinations.

Please discuss with your investigator to evaluate the practical modalities of your withdrawal (in light of your situation), including any follow up-visits or procedures.

In any case, no new data will be sent to the sponsor.

If your biological samples (e.g. blood samples, urine samples) have already been used or analysed before the withdrawal of your consent, the sponsor still has the right to use the results from those tests.

The biological samples that have been collected (but not tested) before the withdrawal of your consent and the data obtained from it, can also still be used by the sponsor. You may ask for a destruction of those samples. If this impacts the validity of the trial, the destruction may be postponed till the end of the trial.

9.2. The investigator decides to end your trial participation

The investigator may end your trial participation because

- it is better for your health,
- he/she determines that you are not following the instructions given to participants, or
- any other reason that will be explained.

9.3. Other entities may interrupt or end the trial

The sponsor, the competent ethics committee and the competent Belgian health authorities may interrupt or end the trial because

- the information gathered shows that the Study Intervention is not effective (does not deliver a sufficient level of improvement in the health of the trial participants),
- the Study Intervention causes more (serious) side effects than anticipated, or
- any other reason that will be duly motivated by such party.

10. Which treatment will I get after my participation in the trial?

After you stopped the treatment with the study intervention, you will receive the best standard treatment available.

11. Will my participation in the trial involve extra costs for me?

11.1. Examinations and treatments paid by the sponsor

The treatments and examinations that are trial specific will be paid by the sponsor and will not be charged to you. The standard procedures or examinations for your condition (i.e. standard of care) will be charged to you or your mutual insurance fund (Belgian social security). Since there is no single standard treatment for COVID-19 and the standard treatment is determined by the treating hospital, it is not possible to list here which therapy and treatments are study specific.

If you need more details or if you are not affiliated with a mutual insurance fund (Belgian social security), please contact the trial staff.

The visits and treatments which are a consequence of a side effect are also considered as trial specific.

12. Which data are collected about me during the trial and what will happen with them?

12.1. Which data are collected and processed during the trial?

The collected and processed personal data concern information about your health and medical condition. This includes your medical history, some of your background information (for example your age, sex, and ethnic origin) and the results of examinations required by the trial.

12.2. How will the investigator treat my personal data?

The investigator is bound by professional secrecy about the data collected.

This means that he/she will never reveal your identity, including in a scientific publication or a lecture and that he/she will encode your data (*that is* by replacing your identity by an identification code in the trial) before sending them to the sponsor.

Therefore, the investigator and the trial staff under the responsibility of the investigator, will be the only ones able to establish a link between your identity and the data transmitted during the trial, with the exceptions listed under section 12.6.

The data transmitted to the sponsor will not allow the sponsor to identify you.

12.3. What will happen to information about me collected during the trial?

Your participation in the trial means that your personal data

- are collected by the investigator, and
- are used in an encoded form by the trial sponsor.

The investigator and the sponsor can only use the encoded personal data for research purposes in connection with scientific publications within the context of the trial that you participate in, or for a broader use of the encoded data if described below.

In addition, the sponsor may provide access to the encoded data to external researchers (that are not involved in this trial). In the event an external researcher wants to use the data in a project not yet described in this document, this project will have to be approved by an Ethics Committee. The data can only be used for studies related to the same pathology. If your encoded trial data are sold, you will not benefit from this.

12.4. How will my data be handled?

Your trial data will be processed in accordance with the General Data Protection Regulation (GDPR, Ref. 2) and the Belgian law on data protection of 30th July 2018 (Ref. 3). The sponsor is responsible for this processing.

Processing your personal data in this trial is allowed because we are conducting scientific research and

- we have to perform a task carried out in the **public interest**

The processing of your personal data is necessary in order to achieve the scientific research purposes described herein. Conducting academic research is part of UZ Leuven's legal assignments. As a university hospital affiliated with KU Leuven, UZ Leuven should support science and education in the public interest.

UZ Leuven pleases you to clarify that the necessity of processing for conducting scientific research and this as a task of general interest constitutes the legal admission ground on the basis of which UZ Leuven processes your data in the context of this research. In addition, UZ Leuven is subject to specific legal obligations that make the processing of your data possible in the context of safety reporting (such as reporting adverse reactions to supervisory authorities)

12.5. Do I have access to my data collected and processed during the trial and can I rectify them ?

You are entitled to ask the investigator what data are being collected about you and how those data will be used in connection with the trial.

You have the right

- to inspect and access these data

- to have all your data erased
- to ask for correction if they are incorrect
- to restrict the processing of your data.

Please ask your investigator when you can have access to your personal data.

12.6. Who, other than the Investigator and his staff, has access to my personal data?

To verify the quality of the trial, it is possible that your personal **un**coded data or information in your medical records relevant for the trial, will be examined by people outside the trial staff but under the responsibility of the investigator. These persons must be subject to professional secrecy or a confidentiality agreement. The following might be considered:

- the personnel designated by the sponsor of the trial (MONITORS and AUDITORS), and people or organisations providing services for or collaborating with the sponsor. They will however never transfer your name and contact details to the sponsor.
- inspectors of competent health authorities worldwide
- an independent audit group
- people designated by the Ethics Committee

For the needs of the trial, the encoded trial data may be sent to other EU and non-EU countries and may be reviewed by

- personnel (other than the inspectors) of competent health authorities of Belgium (Federal agency for medicines and health products, FAMHP) and other EU and non-EU countries,
- the evaluating Belgian Ethics Committee(s),
- external researchers,
- the sponsor of the trial, personnel designated by the sponsor, and people or organisations providing services for or collaborating with the sponsor, and/or

The European regulation and the Belgian legislation on data protection have requirements for transferring data to non-EU countries. The sponsor must ensure equivalent guarantees regarding personal data protection standards before transferring the encoded data to non-EU countries. If for this purpose, there is a data protection agreement, a copy of this agreement may be obtained via the investigator. You can always contact your investigator to obtain more information about any such transfers.

12.7. What will happen to the results of the trial?

After trial closure, a description and the results of this clinical trial will be published in specialised medical journals. A copy of the scientific publication or a summary for laypersons can be obtained from the investigator or the trial staff.

A description of the trial will also be available on https://www.clinicaltrialsregister.eu/ and/or https://www.Clinicaltrials.gov. You can search these websites at any time using the trial number given on the front page of the informed consent form. The websites will include a summary of the results within 1 year after the end of the trial (Ref. 4).

These websites or publications will not include information that can identify you.

12.8. Will my data be used for other purposes than for the trial in which I take part?

The results of the trial will be used to answer the scientific questions of the trial. In addition, the sponsor would like to use your data obtained from this trial, in connection with other research and development activities (and the associated scientific publications). These activities may concern

- the way this study intervention and study interventions of the same group work,
- the disease/condition for which the study intervention is evaluated in this trial or
- other diseases and health problems which could benefit from the study intervention or from related diagnostic tests.
-

Any additional research outside of the trial, must be approved by a Belgian recognized Ethics Committee.

At the end of this form you agree or disagree to the use of your trial data for other purposes by ticking the appropriate check-box in Chapter II.

By consenting to participate in this study you also consent that your data from this study can be used by the funder (KCE) or similar public healthcare research institutes in Europe for further analyses, for example to determine whether one of the treatments studied provides better value.
You agree that for such further analyses your Belgian national number is collected and used by a trusted third party (NAME TTP) to link your study data to data from other sources (healthcare billing data and minimal clinical data sets collected during hospital stay).
Under no circumstances the researchers performing the additional analyses will see your identity and all researchers will be bound by professional confidentiality.

12.9. How long will my data be kept?

After the end of the trial your encoded data will be retained for at least 25 years (Ref. 5) to ensure the validity of the research. This will also be the case if you stopped trial participation prematurely.

13. Which biological samples are collected from me during the trial and what will happen with them?

13.1. Which biological samples are collected from me during the trial?

In this trial, the following biological sample(s) will be taken: blood samples, and nose-throat swabs, for direct analysis.

13.2. What will happen to the collected biological samples?

The collected biological samples will be managed and stored at the biobank of each of the participating institutions, managing the biological material for the sponsor] for 30 years.

These biological samples will be analysed for the objectives of the trial.

13.3. How will my biological samples be handled?

The procedure to encode your biological samples is the same as that used for your personal data (see I § 12.3, page 13, Ref. 6). Samples sent to the sponsor or to organisations working in collaboration with the sponsor, will only be labelled with your trial identification code.

As part of the trial, the sponsor might transfer (a part of) your samples to a laboratory that is working with them. This laboratory may only use your samples as specified in this document. The tracking of your samples will be ensured by the sponsor.

Your biological samples are deemed to be a “donation”. You will not receive any financial benefit associated with the development of new therapies derived from the use of your biological samples, and which may have commercial value.

13.4. What happens with any remainders of biological samples once the analyses described in this document have been carried out?

The sponsor shall use the biological samples within the context of the trial as described above.

Since scientific progress in this area is constant, the sponsor would like to, with your consent retain the remainders of your biological samples for 30 years. The sponsor will use them for future research, outside the trial that you will participate in, to better understand the disease, its treatment and the responses to this treatment. The

retention of the remainders of your samples goes together with the retention of the accompanying encoded personal data.

You agree to the retention of the remainders of your biological samples for future research by consenting for the trial.

If you agree, any future research, additional to what is described above, may only be conducted according to the legislation on the use of human tissue material (Ref. 7) and with the approval of a Belgian recognized Ethics Committee.

Will any additional biological samples be collected and used for additional research?

In this study, blood samples and – optionally – a nasal throat swab will be collected (day 6 of the study), but only if these samples can be done without any other impact on care.

14. Who has reviewed and approved the trial documents?

The documents of the trial have been reviewed by

- The Belgian competent health authorities if applicable, by the competent national health authorities of other EU members states and
- An independent Belgian Ethics Committee

It is the task of the competent health authorities and the Ethics Committees to protect people who take part in a trial. The health authorities will ensure that the trial is conducted in accordance with the applicable legislation.

You should not under any circumstances take their approval as an incentive to take part in the trial.

15. What happens in case of incidental findings?

If by chance and in addition to the trial objectives a result is discovered during the trial that may be important to your health or the health of your blood relatives (called ”incidental findings”), the sponsor will inform the investigator. With your consent the investigator will notify you and your treating physician about your results and potential consequences. If necessary, the investigator and/or the treating physician will advise you on the next steps.

CHAPTER II - INFORMED CONSENT

PARTICIPANT

PREREQUISITES FOR YOUR PARTICIPATION IN THE TRIAL

- I declare that I have been informed of and that I understand the purpose of the clinical trial, its duration, possible risks and discomforts, the precautions that I have to take and what is expected of me. My rights have been explained to me and I have understood those rights.
- I have had enough time to think about taking part in this trial and to discuss it with a trusted person (for example friends, relatives, treating physician, …).
- I have had the opportunity to ask any questions that came to mind and have obtained a satisfactory response to my questions.
- I understand that my participation in this trial is voluntarily and free from any coercion and that I am free to stop at any time my trial participation.
- I understand that data about me will be collected and that they will be treated confidentially.
- I understand that for UZ Leuven conduct of this study serves the public interest and the processing of my personal data is necessary for carrying out this study.
- I authorise the transfer to and processing of my encrypted data in countries outside the EU without equivalent level of protection as necessary for the study.
- I understand that the sponsor has taken out an insurance in case I should suffer any damage in connection with my participation in this trial.
- I understand that when participating in this trial, I will not have any costs except those related to the standard of care treatment of my disease
- I agree to my treating physician(s) being informed of my participation in this trial.
- I agree not to take part in any other trial at the same time without first informing the investigator or the trial staff, who might not permit me to participate for a good reason.
- I understand that I need to cooperate and follow the investigator's and trial staff's instructions regarding the trial.
- I understand that participation to the trial might end for me without my consent if I need other treatment, do not follow the trial plan, have a trial-related injury, or for any other justified reason.
- I certify that all the information I have given about my medical history is correct. I understand that my failure to inform the investigator or designee about any exclusion criteria may harm myself.
- If written consent is not possible due to security risks associated with transmission of the new corona virus, written consent will be delayed until this can be done safely. Pending written consent, your oral consent (or that of your legal representative) will be required. This oral consent is provided in the

presence of a witness and will be documented in the medical file before starting the study procedures.

As specified in Chapter I, § 13.4, page 16, the sponsor would like to retain the remainders of your biological samples for 30 years for future research outside the trial that you will participate in. The samples will be used to better understand the disease, its treatment and the responses to this treatment.

Do you agree with the retention of the remainders of your biological samples and the accompanying personal data for future research outside the trial?

(Tick as appropriate. If you leave this question open, we assume the answer is ‘I do not agree’.)

☐ **I agree**	☐ **I do not agree**

I consent to take part in the trial, and I have received a signed and dated copy of all pages of this document.

Participant’s surname and first name:

Date (DD/MMM/YYYY):

Time:

Participant’s signature:

LEGAL REPRESENTATIVE (REF. 8)

I declare that I have been informed that I am being asked to take a decision on whether or not to take part in the clinical trial for the person I represent, considering his/her best interests and taking into consideration his/her likely wishes. My consent applies to all the items listed in the consent of the participant.

I have also been informed that as soon as the clinical situation allows, the person I represent will be made aware of his/her participation in a clinical trial and from that point will be free to continue with this participation or end it by signing or refusing to sign this consent form.

I have received a signed and dated copy of this document.

Legal representative's surname and first name:

Relationship to the participant:

Date (DD/MMM/YYYY):

Time:

Legal representative's signature:

IMPARTIAL WITNESS / INTERPRETER (REF. 9)

I, the undersigned (Tick as appropriate),

☐ Impartial Witness

☐ Interpreter

was present during the entire process of informing the participant and I confirm that the information on the objectives and procedures of the trial was adequately provided, that the participant (or his/her legal representative) apparently understood the trial and that consent to participate in the trial was freely given.

Impartial Witness / Interpreter surname and first name:

Impartial Witness / Interpreter qualification:

Date (DD/MMM/YYYY):

Time:

Impartial Witness / Interpreter signature:

INVESTIGATOR

I, the undersigned investigator, confirm that

- the participant has been verbally provided with the necessary information about the trial, has been explained the content and has been given an original signed document.
- I have verified that the participant has understood the trial.
- I have given the participant sufficient time to agree to take part and to ask any questions.
- no pressure was applied to persuade the participant to agree to take part in the trial.
- I operate in accordance with the ethical principles set out in the latest version of the “Helsinki Declaration”, the “Good Clinical Practices” and the Belgian Law (Ref. 10).

Investigator’s delegate, surname and first name:

Investigator’s delegate, qualification:

Date (DD/MMM/YYYY):

Time:

Investigator’s delegate signature:

Investigator’s, Surname and first name:

Date (DD/MMM/YYYY):

Time:

Investigator’s signature:

GLOSSARY

DPA: The Data Protection Authority ensures that personal data are handled with care and thoroughly protected, and that your future privacy also remains guaranteed.

KCE: Federaal Kenniscentrum voor de gezondheidszorg - Federal Knowledge Centre for Health Care

NO FAULT INSURANCE:

The sponsor is liable for any injury or any damage that the participant has suffered, and which is directly or indirectly related to the clinical trial. You do not have to prove any mistake in this respect.

MONITOR and AUDITOR

Both the monitor and auditor work for the sponsor.
The monitor takes care of a continuous quality check during the course of a trial. The auditor performs a quality check after the trial. They verify if the trial is being/was conducted according to the protocol, if the reported data are liable and if the clinical trial was conducted according the applicable rules.

REFERENCES

[1] This is in accordance with Article 29 of the Belgian Law of 7 May 2004 related to experiments on humans.

[2] General Data Protection Regulation No 2016/679 of the European Parliament and of the council of 27 April 2016 on the protection of natural persons with regard to the processing of personal data and on the free movement of such data, and repealing Directive 95/46/EC.

[3] The Belgian Law of 30 July 2018 on the protection of natural persons with regard to the processing of personal data.

[4] In accordance with section 4.3. of the Commission Guideline: Guidance on posting and publication of result-related information on clinical trials in relation to the implementation of Article 57(2) of Regulation (EC) No 726/2004 and Article 41(2) of Regulation (EC) No 1901/2006 - (2012/C 302/03). [From the moment the Clinical trial regulation enters into force : In accordance with article 37 of the Clinical trial regulation No 536/2014 of the European Parliament and of the council of 16 April 2014 on clinical trials on medicinal products for human use, and repealing Directive 2001/20/EC; sponsor have to provide summary results of clinical trials in a format understandable to laypersons.]

[5] In accordance with article 58 of the Clinical trial regulation No 536/2014 of the European Parliament and of the council of 16 April 2014 on clinical trials on medicinal products for human use, and repealing Directive 2001/20/EC.

[6] Belgian Law of 19 December 2008 on the acquisition and use of human body material with a view to medical application to humans or scientific research, and the applicable royal decrees.

[7] This is in accordance with Article 21 of the Belgian Law of 19 December 2008 on the acquisition and use of human body material with a view to medical application to humans or scientific research, and the applicable royal decrees.

[8] When a person of full age is incapable of expressing his will, legal representation must be used which is determined in successive order (administrator, or failing that, the spouse, the legal cohabiting partner, de facto cohabiting partner, an adult child, a parent, an adult brother or sister). The regulation is laid down in article 8 of the law of 7 May 2004 on experiments on the human person.

[9] Use of an impartial witness is necessary when either the subject or the subject's legally authorized representative speaks and/or fully understands the language of the approved informed consent form, but cannot read and write due to any physical impairment or is visually impaired. An interpreter is necessary when the investigator doesn't speak the language of the patient.

[10] Belgian Law of 7 May 2004 related to experiments on humans, and the applicable royal decrees.
