# Supplementary material for: A randomized, multicentre, open-label phase II proof-of-concept trial investigating the clinical efficacy and safety of the addition of convalescent plasma to the standard of care in patients hospitalized with COVID-19: the Donated Antibodies Working against nCoV (DAWn-Plasma) trial
Source: Trials. 2020 Nov 27;21:981. doi: 10.1186/s13063-020-04876-0 (PMC7691949; doi:10.1186/s13063-020-04876-0)
Supplement: Supplementary file 4 — Additional file 4. [file 13063_2020_4876_MOESM4_ESM.pdf]

Screening for Eligibility

Subject number ______________________

eCRF v. 1.0 - Protocol v. 2.3 - 27-04-2020

INFORMED CONSENT

Has informed consent been obtained (verbal or written)? ◯ Yes ◯ No

DO NOT PROCEED IF INFORMED CONSENT HAS NOT BEEN OBTAINED

Date verbal informed consent obtained ______________________

Has signed informed consent been obtained? ◯ Yes ◯ No

Date signed informed consent obtained ______________________

If No, give reason ______________________

SUBJECT ELIGIBILITY

Have all inclusion criteria been met and are none of the exclusion criteria met? ◯ Yes ◯ No
(DO NOT ENROLL PATIENT IF ALL ELIGIBILITY CRITERIA HAVE NOT BEEN MET)

Indicate which inclusion criteria were NOT MET:

- ☐ Subject (≥18 years old) or legally authorized representative provides informed consent prior to initiation of any study procedures. When signed informed consent is not possible (e.g. due to restrictions to prevent viral transmission), verbal informed consent in the presence of a witness will be obtained and documented in the medical files. Signed informed consent will be obtained as soon as the safety concerns are mitigated.
- ☐ Subject (or legally authorized representative) understands and agrees to comply with planned study procedures.
- ☐ Male or non-pregnant female adult ≥18 years of age at time of enrolment.
- ☐ Patient should be hospitalized
- ☐ Has a confirmed diagnosis of SARS-CoV-2 infection, defined as either a) lab laboratory-confirmed SARS-CoV-2 infection as determined by PCR, or other commercial or public health assay in any specimen as diagnosed within 60 hours prior to randomization or b) the combination of upper or lower respiratory infection symptoms (fever, cough, dyspnea, desaturation) and typical findings on chest CT scan and absence of other plausible diagnoses
- ☐ Illness of any duration, and at least one of the following: a) Radiographic infiltrates by imaging (chest x-ray, CT scan, etc.), OR b) Clinical assessment (evidence of rales/crackles on exam) AND SpO2 ≤ 94% on room air OR c) Requiring mechanical ventilation and/or supplemental oxygen.
- ☐ ABO D typing of the patient should be done at least once and the result should be known.

Indicate which exclusion criteria WERE MET:

☐ Receiving invasive (any mode where a patient has been intubated endotracheally, or via tracheostomy) or non-invasive (for instance, but not restricted to CPAP, PSV, PCM, SiMV) mechanical ventilation before or upon randomization
☐ Pregnancy or breast feeding
☐ Any medical condition which would impose an unacceptable safety hazard by participation to the study
☐ Patients with a documented grade 3 allergic reaction after the administration of fresh frozen plasma (i.e. systemic reaction with cardiovascular and/or respiratory involvement)
☐ Patients that have treatment restriction that excludes mechanical ventilation and/or endotracheal intubation

SCREEN FAILURE

Did the subject fail screening for any reason other than not meeting eligibility criteria? ◯ Yes ◯ No

Main reason for screen failure
◯ Physician decision
◯ Subject decision (withdrawal)
◯ Death
◯ Lost contact with subject
◯ Technical concerns
◯ Logistical concerns / lack of manpower
◯ Needed mechanical ventilation before plasma infusion
◯ No compatible convalescent plasma
◯ Other reason

Date of death
____

If reason for screen failure is "Other reason", please specify:
____

DEMOGRAPHICS

Date of admission
____

What is the participant's age
____
(years (must be > or = to 18))

Age range
◯ 18-19
◯ 20-29
◯ 30-39
◯ 40-49
◯ 50-59
◯ 60-69
◯ 70-79
◯ 80-89
◯ 90-99
◯ 100-109
(e.g. 30-39, 40-49, etc.)

Participant's gender
◯ Female
◯ Male
◯ Undifferentiated
◯ Unknown

What is the participant's ethnicity?	◯ 1. Causasian<br>◯ 2. North African<br>◯ 3. Middle east<br>◯ 4. Black or sub-sahara (africa)<br>◯ 5. Asian<br>◯ 6. Latino or hispanic<br>◯ 7. Pacific islands<br>◯ 8. Native american or Alaska native
Is the participant pregnant?	◯ Yes ◯ No ◯ Unknown
Body weight	______________________________<br>(kg.)

MEDICAL HISTORY

History of diabetes	◯ Yes ◯ No ◯ Unknown
Is the participant insulin-dependent	◯ Yes ◯ No
History of arterial hypertension	◯ Yes ◯ No ◯ Unknown
History of arrhythmia	◯ Yes ◯ No ◯ Unknown
Smoking	◯ Active ◯ Former ◯ Never
Chronic pulmonary disease (not asthma or COPD)	◯ Yes ◯ No ◯ Unknown
If Chronic pulmonary disease, please specify:	______________________________
COPD	◯ Yes ◯ No ◯ Unknown
Asthma	◯ Yes ◯ No ◯ Unknown
heart failure	◯ Yes ◯ No ◯ Unknown
Ischemic heart disease	◯ Yes ◯ No ◯ Unknown
Moderate or severe liver disease	◯ Yes ◯ No ◯ Unknown
mild liver disease	◯ Yes ◯ No ◯ Unknown
Chronic kidney disease	◯ Yes ◯ No ◯ Unknown
Please specify last known eGRF:	______________________________
Does the participant require dialysis for chronic kidney disease?	◯ Yes ◯ No

Active cancer	◯ Yes ◯ No ◯ Unknown
Please specify which type of cancer:	__________________
History of cancer	◯ Yes ◯ No ◯ Unknown
Please specify type of cancer	__________________
Chronic hematologic disease	◯ Yes ◯ No ◯ Unknown
Chronic neurologic disorder	◯ Yes ◯ No ◯ Unknown
HIV/Aids	◯ Yes ◯ No ◯ Unknown
Other relevant chronic diseases	◯ Yes ◯ No ◯ Unknown
Specify which other relevant chronic disease	__________________

MAINTENANCE MEDICATION

Is the participant treated with antihypertensive drugs	◯ yes ◯ no ◯ Unknown
Please specify which antihypertensive drugs	☐ ACE inhibitor ☐ Angiotensin receptor blocker ☐ Other
Please specify which 'other' antihypertensive drugs	__________________
Please specify which 'other' antihypertensive drugs 2 (if applicable)	__________________
Antiplatelet agent	◯ Yes ◯ No ◯ Unknown
Anticoagulation	◯ Yes ◯ No ◯ Unknown
Statines	◯ Yes ◯ No ◯ Unknown
Oral Antidiabetics	◯ Yes ◯ No ◯ Unknown
Insulin	◯ Yes ◯ No ◯ Unknown
Chronic systemic corticosteroid therapy	◯ Yes ◯ No ◯ Unknown
Other immune-suppressing therapy	◯ Yes ◯ No ◯ Unknown
Please specify which other immune-suppressing therapy	__________________

Antibiotics	◯ Yes ◯ No ◯ Unknown
Please specify : antibiotic 1	__________________
Please specify: antibiotic 2 (if applicable)	__________________

THROMBOEMBOLIC EVENT

Was the patient diagnosed with a thrombeombolic event at the baseline visit	◯ Yes ◯ No
Specify which thromboembolic event	__________________

EQ-5D-5L questionnaire

eCRF v. 1.0 - Protocol v. 2.3 - 27-04-2020

EQ-5D-5L questionnaire completed?	◯ Yes ◯ No
Completion date	________________________
Indicate why questionnaire was not completed	☐ patient's condiction<br>☐ logistical reasons<br>☐ other
specify other	________________________

EQ-5D-5L questionnaire

MOBILITY	◯ I have no problems in walking about<br>◯ I have slight problems in walking about<br>◯ I have moderate problems in walking about<br>◯ I have severe problems in walking about<br>◯ I am unable to walk about
SELF-CARE	◯ I have no problems washing or pressing myself<br>◯ I have slight problems washing or pressing myself<br>◯ I have moderate problems washing or pressing myself<br>◯ I have severe problems washing or pressing myself<br>◯ I am unable to wash or dress myself
USUAL ACTIVITIES	◯ I have no problems doing my usual activities<br>◯ I have slight problems doing my usual activities<br>◯ I have moderate problems doing my usual activities<br>◯ I have severe problems doing my usual activities<br>◯ I am unable to do my usual activities<br>(e.g. work, study, housework, family or leisure activities)
PAIN/DISCOMFORT	◯ I have no pain or discomfort<br>◯ I have slight pain or discomfort<br>◯ I have moderate pain or discomfort<br>◯ I have severe pain or discomfort<br>◯ I have extreme pain or discomfort
ANXIETY/DEPRESSION	◯ I am not anxious or depressed<br>◯ I am slightly anxious or depressed<br>◯ I am moderately anxious or depressed<br>◯ I am severely anxious or depressedd<br>◯ I am extremely anxious or depressed

We would like to know how good or bad your health is today.
This scale is numbered from 0 to 100.
100 means the best health you can imagine.
0 means the worst health you can imagine.

Mark an X on the scale to indicate how your health is TODAY

0 Your Health TODAY 100

(Place a mark on the scale above)

Please write the number you marked on the scale

____

Baseline

eCRF v. 1.0 - Protocol v. 2.3 - 27-04-2020

BASELINE (before administration of plasma)

Date baseline visit

____

Onset date of first/earliest symptoms

____

Highest temperature measured at admission

____

VITAL SIGNS AT BASELINE

Systolic blood pressure on first evalution (mmHg)

____

Diastolic blood pressure on first evaluation (mmHg)

____

Oxygen saturation at first presentation

____
(%)

Lowest measured oxygen saturation when breathing room air

____
(%)

Does the participant need Oxygen therapy?

○ No ○ Flow (L/min)
○ FiO2 (%)

Provide value for Oxygen flow (L/min)

____
(% L/min)

Signs of respiratory distress at first presentation

(i.e. Sat< 93% OR pO2/FiO2 < 300 OR RR >3 0/min)

○ Yes ○ No ○ Unknown

Consciousness level

○ Alert ○ Verbal ○ Pain
○ Unresponsive

Hospitalization status

○ Ward ○ Intensive care unit
○ ER

CHRONIC RESPIRATORY SUPPORT AT HOME

Did the patient receive chronic respiratory support at home? ◯ yes ◯ no ◯ unknown

Specify
◯ Oxygen support
◯ Non-invasive ventilation (BIPAP/CIPAP)

MEDICATION

Specific treatment for COVID-19 used ◯ Yes ◯ No

Indicate which specific treatment (s) for COVID-19 is used
☐ Chloroquine
☐ Hydroxychloroquine
☐ Favipiravir
☐ Remdesivir
☐ Tocilizumab
☐ Lopinavir/Ritonavir
☐ Other

Which specific other treatment(s) for COVID-19 is used __________________________________

Other antiviral drugs used ◯ Yes ◯ No

Indicate which other antiviral drugs is used __________________________________

Indicate which other antiviral drugs is used (if applicable) __________________________________

Antibiotics used at admission ◯ Yes ◯ No

Antibiotic 1 __________________________________

Antibiotic 2 (if applicable) __________________________________

Antibiotic 3 (if applicable) __________________________________

Antifungal treatment used ◯ Yes ◯ No

Indicate which antifungal treatment used __________________________________

Indicate which antifungal treatment used (if applicable) __________________________________

Randomisation

Randomization The randomization button will appear after all necessary fields have been completed.

eCRF v. 1.0 - Protocol v. 2.3 - 27-04-2020

FIRST ABO D TYPING

Date and time first ABO D typing	__________________
Indicate blood type	◯ A ◯ B ◯ AB ◯ O
Re-enter blood type of first blood sample for confirmation	◯ A ◯ B ◯ AB ◯ O
Indicate Rhesus factor	◯ positive ◯ negative
Re-enter Rhesus factor of first blood sample for confirmation	◯ positive ◯ negative
Is second ABO D typing performed?	◯ Yes ◯ No
Date and time second ABO D typing	__________________
Blood type second ABO D typing	◯ A ◯ B ◯ AB ◯ 0
Rhesus factor second ABO D typing	◯ positive ◯ negative

AVAILABILITY COMPATIBLE CONVALESCENT PLASMA!

Is sufficiant compatible convalescent plasma available to be delivered to the hospital for bloodtype [ran_bloodtype], rhesus factor [ran_rf1]	◯ Yes ◯ No
Email(Work variable, used for sending randomization confirmation)	__________________
Site (Work variable, used for sending randomization confirmation)	__________________
Name of randomizing person	__________________
DAWN-PLASMA randomization	
Date and time of Randomization	__________________

After randomization: Please, don't forget to set the Form Status to 'COMPLETED' before saving this page. A confirmation email and RED CROSS email is sent only after the page Form Status is set to 'Complete'.

To randomize, click the 'randomize' button

Studyarm	◯ SOC+PLASMA<br>◯ SOC

Second ABO D Typing

eCRF v. 1.0 - Protocol v. 2.3 - 27-04-2020

SECOND ABO D TYPING

Date and time second ABO D typing ______________________

Blood type second ABO D typing ◯ A ◯ B ◯ AB ◯ 0

Rhesus factor second ABO D typing ◯ positive ◯ negative

ECG monitoring

eCRF version 1.0 - Protocol version 2.2 - 24-04-2020

ECG performed?	◯ Yes ◯ No
QTc value (ms)<br>(Fredericia formule : QTc = QT interval / (RR interval)1/3) calculator	__________________<br>(ms)

1st Transfusion Plasma

eCRF v. 1.0 - Protocol v. 2.3 - 27-04-2020

Plasma treatment arm	◯ Yes ◯ No

ADMINISTRATION OF BLOOD PRODUCT WITHIN 12 HRS AFTER RANDOMIZATION

First transfusion of convalescent plasma given?	◯ Yes ◯ No
Give reason	______________________
Date and time first transfusion of convalescent plama	______________________
Number of transfused units	______________________
Volume of transfused units	______________________
Duration of transfusion	______________________
Anti-body titre of transfused units	______________________
Did transfusion related side effects occur?	◯ Yes ◯ No<br>(e.g. acute lung injury, serious allergic transfusion reactions, transfusion associated circulatory overload.)
Please specify	☐ Acute lung injury<br>☐ Serious allergic transfusion reactions<br>☐ Transfusion associated circulatory overload<br>☐ Other
Specify other	______________________

If this is considered an Adverse Event Grade 4, please complete the Adverse Events page [adverse_event_arm_1][form-link:adverse_events: Link to all patient adverse_events (first record)] - Total number of records: [adverse_event_arm_1][ae_total][last-instance]

2nd Transfusion Plasma

eCRF v. 1.0 - Protocol v. 2.3 - 27-04-2020

Plasma treatment arm	◯ Yes ◯ No

ADMINISTRATION OF BLOOD PRODUCT 24-36 HRS AFTER FIRST ADMINISTRATION

Second transfusion of convalescent plasma given?	◯ Yes ◯ No
Give reason	__________________________________
Date and time second transfusion of convalescent plama	____________________________
Number of transfused units	____________________________
Volume of transfused units	____________________________
Duration of transfusion	____________________________
Anti-body titre of transfused units	____________________________
Did transfusion related side effects occur?	◯ Yes ◯ No<br>(e.g. acute lung injury, serious allergic transfusion reactions, transfusion associated circulatory overload.)
Please specify	☐ Acute lung injury<br>☐ Serious allergic transfusion reactions<br>☐ Transfusion associated circulatory overload<br>☐ Other
Specify other	____________________________

If this is considered an Adverse Event Grade 4, please complete the Adverse Events page [adverse_event_arm_1][form-link:adverse_events: Link to all patient adverse_events (first record)] - Total number of records: [adverse_event_arm_1][ae_total][last-instance]

Clinical Status

eCRF v. 1.0 - Protocol v. 2.3 - 27-04-2020

CLINICAL STATUS

Clinical status recorded on	______________________
Clinical status	◯ Uninfected.Non viral RNA detected<br>◯ Ambulatory, Asymptomatic, viral RNA detected<br>◯ Ambulatory, Symptomatic, Independent<br>◯ Ambulatory, Symptomatic, Assistance needed<br>◯ Hospitalized, mild disease, No oxygen therapy needed<br>◯ Hospitalized, mild disease, Oxygen by mask or nasal prongs<br>◯ Hospitalized, severe disease, Oxygen by NIV or High flow<br>◯ Hospitalized, severe disease, Intubation and mechanical ventilation (pO2/FiO2>=150 OR SpO2/FiO2>=200)<br>◯ Hospitalized, severe disease, Mechanical ventilation (pO2/FiO2< 150 OR SpO2/FiO2< 200) OR vasopressors (norepinephrine > 0.3microg/kg/min)<br>◯ Hospitalized, severe disease, Mechanical ventilation (pO2/FiO2< 150 AND vasopressors (norepinephrine > 0.3microg/kg/min), OR Dialysis OR ECMO<br>◯ Death, Dead
Oxygen support at home?	◯ Yes ◯ No
Hospitalization status at 08:00 am	◯ Ward<br>◯ Intensive Care Unit
SOFA-score (enter a number from 0-24	______________________<br>(Enter a number from 0 to 24)
APACHE-II score	______________________<br>(Enter a number from 0 to 71)
Cause of death	◯ respiratory failure<br>◯ multiorgan failure<br>◯ heart failure<br>◯ sudden unexpected or arrhythmic death
Amount of oxygen given (FiO2)	______________________<br>(Complete this field for the first 14 days of admission)
Highest body temperature measured	______________________<br>(°c)

Visit

eCRF v. 1.0 - Protocol v. 2.3 - 27-04-2020

VISIT (complete daily until discharge)

Visit performed	◯ Yes ◯ No
Visit date	______________________
If visit was not done, please provide reason	◯ Subject is lost to follow up<br>◯ Subject withdrew consent<br>◯ Subject died<br>◯ Other<br>◯ Subject left the hospital (dismissed)
Date of discharge	______________________
Specify "Other" reason why visit was not done	______________________
Was the patient in ICU for the past 24 hours ?	◯ Yes ◯ No
Signs of respiratory distress<br><br>(i.e. Sat < 93% OR pO2/FiO2 < 300 or RR > 30/min)	◯ Yes ◯ No ◯ Unknown

Support

Is the patient currently receiving, or has received (between 00:00 to 24:00 on the day of assessment) (apply to all questions in this section):

Oxygen support	◯ Yes ◯ No ◯ Unknown
High flow oxygen support(e.g. optiflow)	◯ Yes ◯ No ◯ Unknown
Non invasive ventilation (e.g. BIPAP, CPAP)	◯ Yes ◯ No ◯ Unknown
Invasive ventilation	◯ Yes ◯ No ◯ Unknown
Prone ventilation	◯ Yes ◯ No ◯ Unknown
Extra corporeal life support(ECMO)	◯ Yes ◯ No ◯ Unknown
Inhaled nitric oxide	◯ Yes ◯ No ◯ Unknown
Dialysis/Hemofiltration	◯ Yes ◯ No ◯ Unknown
Specific treatment used other than plasma	◯ Yes ◯ No

Specify treatments	☐ Chloroquine<br>☐ Hydroxychloroquine<br>☐ Favipiravir<br>☐ Remdesivir<br>☐ Tocilizumab<br>☐ Lopinavir/Ritonavir<br>☐ Other
Specify 'Other'	__________________________________
Other antiviral drugs used	○ Yes ○ No
Specifiy other antiviral drug 1	__________________________________
Specify other antiviral drug 2 (if applicable)	__________________________________
Antibiotics used	○ Yes ○ No
Specifiy antibiotics 1	__________________________________
Specify antibiotics 2 (if applicable)	__________________________________
Antifungal treatment used	○ Yes ○ No
Specify antifungal treatment 1	__________________________________
Specify antifungal treatment 2 (if applicable)	__________________________________
Is the patient receiving ACE-inbitors or Angiotensine Receptor-Blockers?	○ Yes ○ No
Specify	__________________________________
Is the patient receiving NSAID?	○ Yes ○ No
Specify NSAID 1	__________________________________
Specify NSAID 2 (if applicable)	__________________________________
Is the patient receiving systemic corticosteroids?	○ Yes ○ No
Specify	__________________________________

SERIOUS ADVERSE EVENTS AND ADVERSE EVENTS GRADED AS SEVERE

Did new adverse events occur since previous visit, or have there been changes in previously reported conditions?	◯ Yes ◯ No
Was Adverse Events page completed?	◯ Yes<br>◯ No<br>◯ Not applicable (non-reportable event)

Please complete Adverse Events page
[adverse_event_arm_1][form-link:adverse_events: Link to all patient adverse_events (first record)] - Total number of records: [adverse_event_arm_1][ae_total][last-instance]

Visit D15

eCRF v. 1.0 - Protocol v. 2.3 - 27-04-2020

VISIT D15

Visit performed (either by phone or in hospital)	◯ Yes ◯ No
Patient still hospitalized	◯ Yes ◯ No
Visit date	________________
If visit was not done, please provide reason	◯ Subject is lost to follow up<br>◯ Subject withdrew consent<br>◯ Subject died<br>◯ Other<br>◯ Subject left the hospital (dismissed)
Date of death	________________
Date of discharge	________________
If "Other" reason, please explain	________________
Was the patient readmitted in the hospital after discharge?	◯ Yes ◯ No
Date of readmission	________________
Was the patient readmitted due to COVID-19?	◯ Yes ◯ No
If not, what was the reason for hospitalisation?	________________

SUPPORT

Is the patient currently receiving, or has received (between 00:00 to 24:00 on the day of assessment) (apply to all questions in this section):

Oxygen support	◯ Yes ◯ No ◯ Unknown
High flow oxygen support(e.g. optiflow)	◯ Yes ◯ No ◯ Unknown
Non invasive ventilation (e.g. BIPAP, CPAP)	◯ Yes ◯ No ◯ Unknown
Invasive ventilation	◯ Yes ◯ No ◯ Unknown

Prone ventilation	◯ Yes ◯ No ◯ Unknown
Extra corporeal life support(ECMO)	◯ Yes ◯ No ◯ Unknown
Inhaled nitric oxide	◯ Yes ◯ No ◯ Unknown
Dialysis/Hemofiltration	◯ Yes ◯ No ◯ Unknown

SYMPTOMS

Signs of respiratory distress<br><br>(i.e. Sat < 93% OR pO2/FiO2 < 300 or RR > 30/min)	◯ Yes ◯ No ◯ Unknown
Select symptoms that occured during last 24 hours	☐ Fever<br>☐ Sore throat<br>☐ Runny nose<br>☐ Shortness of breath<br>☐ Cough<br>☐ Wheezing<br>☐ Chest pain<br>☐ Muscle aches (Myalgia)<br>☐ Joint pain (Arthralagia)<br>☐ Fatigue/Malaise<br>☐ Headache<br>☐ Altered conciousness/confusion<br>☐ Seizures<br>☐ Abdominal pain<br>☐ Nausea<br>☐ Decreased apitite<br>☐ Vomiting<br>☐ Diarrhea<br>☐ Conjunctivitis<br>☐ Skin rash<br>☐ Skin ulcers<br>☐ Lymphadenopathy<br>☐ Sleep disorders and disturbances<br>☐ Bleeding(Haemorrhage)

SERIOUS ADVERSE EVENTS AND ADVERSE EVENTS GRADED AS SEVERE (i.e. AE that are life-threatening and/or require an urgent intervention)

Did new adverse events occur since previous visit, or have there been changes in previously reported conditions?	◯ Yes ◯ No
Was Adverse Events page completed?	◯ Yes ◯ No ◯ Not applicable (non-reportable event)

Please complete Adverse Events page [adverse_event_arm_1][form-link:adverse_events: Link to all patient adverse_events (first record)] - Total number of records: [adverse_event_arm_1][ae_total][last-instance]

Visit D30

eCRF v. 1.0 - Protocol v. 2.3 - 27-04-2020

VISIT D30

Visit performed (either by phone or in hospital)	◯ Yes ◯ No
Patient still hospitalized	◯ Yes ◯ No
Visit date	__________________
If visit was not done, please provide reason	◯ Subject is lost to follow up<br>◯ Subject withdrew consent<br>◯ Subject died<br>◯ Other<br>◯ Subject left the hospital (dismissed)
Date of death	__________________
Date of discharge	__________________
Specify "Other" reason why visit was not done	__________________
Was the patient readmitted in the hospital after discharge?	◯ Yes ◯ No
Date of readmission	__________________
Was the patient readmitted due to COVID-19?	◯ Yes ◯ No
If not, what was the reason for hospitalisation?	__________________

SUPPORT

Is the patient currently receiving, or has received (between 00:00 to 24:00 on the day of assessment) (apply to all questions in this section):

Oxygen support	◯ Yes ◯ No ◯ Unknown
High flow oxygen support(e.g. optiflow)	◯ Yes ◯ No ◯ Unknown
Non invasive ventilation (e.g. BIPAP, CPAP)	◯ Yes ◯ No ◯ Unknown
Invasive ventilation	◯ Yes ◯ No ◯ Unknown

Prone ventilation	◯ Yes ◯ No ◯ Unknown
Extra corporeal life support(ECMO)	◯ Yes ◯ No ◯ Unknown
Inhaled nitric oxide	◯ Yes ◯ No ◯ Unknown
Dialysis/Hemofiltration	◯ Yes ◯ No ◯ Unknown

SYMPTOMS

Signs of respiratory distress<br><br>(i.e. Sat < 93% OR pO2/FiO2 < 300 or RR > 30/min)	◯ Yes ◯ No ◯ Unknown
Select symptoms that occured during last 24 hours	☐ Fever<br>☐ Sore throat<br>☐ Runny nose<br>☐ Shortness of breath<br>☐ Cough<br>☐ Wheezing<br>☐ Chest pain<br>☐ Muscle aches (Myalgia)<br>☐ Joint pain (Arthralagia)<br>☐ Fatigue/Malaise<br>☐ Headache<br>☐ Altered conciousness/confusion<br>☐ Seizures<br>☐ Abdominal pain<br>☐ Nausea<br>☐ Decreased apitite<br>☐ Vomiting<br>☐ Diarrhea<br>☐ Conjunctivitis<br>☐ Skin rash<br>☐ Skin ulcers<br>☐ Lymphadenopathy<br>☐ Sleep disorders and disturbances<br>☐ Bleeding(Haemorrhage)

SERIOUS ADVERSE EVENTS AND ADVERSE EVENTS GRADED AS SEVERE (i.e. AE that are life-threatening and/or require an urgent intervention)

Did new adverse events occur since previous visit, or have there been changes in previously reported conditions?	◯ Yes ◯ No
Was Adverse Events page completed?	◯ Yes ◯ No ◯ Not applicable (non-reportable event)

Please complete Adverse Events page [adverse_event_arm_1][form-link:adverse_events: Link to all patient adverse_events (first record)] - Total number of records: [adverse_event_arm_1][ae_total][last-instance]

Visit D90

eCRF v. 1.0 - Protocol v. 2.3 - 27-04-2020

VISIT D90

Visit performed (either by phone or in hospital)	◯ Yes ◯ No
Patient still hospitalized	◯ Yes ◯ No
Visit date	______________________
If visit was not done, please provide reason	◯ Subject is lost to follow up<br>◯ Subject withdrew consent<br>◯ Subject died<br>◯ Other<br>◯ Subject left the hospital (dismissed)
Date of death	______________________
Date of discharge	______________________
Specify "Other" reason why visit was not done	______________________
Was the patient readmitted in the hospital after discharge?	◯ Yes ◯ No
Date of readmission	______________________
Was the patient readmitted due to COVID-19?	◯ Yes ◯ No
If not, what was the reason for hospitalisation?	______________________

SUPPORT

Is the patient currently receiving, or has received (between 00:00 to 24:00 on the day of assessment) (apply to all questions in this section):

Oxygen support	◯ Yes ◯ No ◯ Unknown
High flow oxygen support(e.g. optiflow)	◯ Yes ◯ No ◯ Unknown
Non invasive ventilation (e.g. BIPAP, CPAP)	◯ Yes ◯ No ◯ Unknown
Invasive ventilation	◯ Yes ◯ No ◯ Unknown

Prone ventilation	◯ Yes ◯ No ◯ Unknown
Extra corporeal life support(ECMO)	◯ Yes ◯ No ◯ Unknown
Inhaled nitric oxide	◯ Yes ◯ No ◯ Unknown
Dialysis/Hemofiltration	◯ Yes ◯ No ◯ Unknown

SYMPTOMS

Signs of respiratory distress<br><br>(i.e. Sat < 93% OR pO2/FiO2 < 300 or RR > 30/min)	◯ Yes ◯ No ◯ Unknown
Select symptoms that occured during last 24 hours	☐ Fever<br>☐ Sore throat<br>☐ Runny nose<br>☐ Shortness of breath<br>☐ Cough<br>☐ Wheezing<br>☐ Chest pain<br>☐ Muscle aches (Myalgia)<br>☐ Joint pain (Arthralagia)<br>☐ Fatigue/Malaise<br>☐ Headache<br>☐ Altered conciousness/confusion<br>☐ Seizures<br>☐ Abdominal pain<br>☐ Nausea<br>☐ Decreased apitite<br>☐ Vomiting<br>☐ Diarrhea<br>☐ Conjunctivitis<br>☐ Skin rash<br>☐ Skin ulcers<br>☐ Lymphadenopathy<br>☐ Sleep disorders and disturbances<br>☐ Bleeding(Haemorrhage)

SERIOUS ADVERSE EVENTS AND ADVERSE EVENTS GRADED AS SEVERE (i.e. AE that are life-threatening and/or require an urgent intervention)

Did new adverse events occur since previous visit, or have there been changes in previously reported conditions?	◯ Yes ◯ No
Was Adverse Events page completed?	◯ Yes ◯ No ◯ Not applicable (non-reportable event)

Please complete Adverse Events page [adverse_event_arm_1][form-link:adverse_events: Link to all patient adverse_events (first record)] - Total number of records: [adverse_event_arm_1][ae_total][last-instance]

PLEASE COMPLETE END OF STUDY VISIT.

Please complete End of Study Visit

Lab values

eCRF v. 1.0 - Protocol v. 2.3 - 27-04-2020

Blood sample taken?	◯ Yes ◯ No
Date lab values	________________
CRP (mg/L)	________________ (mg/L)
White blood cell count (x10^9/L)	________________
Absolute lymphocyte count (10*9/L)	________________
Absolute neutrophil count (10*9/L)	________________
Absolute eosinophil count (10*9/L)	________________
Hemoglobin (g/dl)	________________
Platelet count (x10^9/L)	________________
Serum creatinin (mg/dl)	________________
AST (U/L)	________________
ALT (U/L)	________________
Alkaline phosphatase (U/L)	________________
Potassium	________________ (mmol/L)
Total Bilirubin	________________ (mg/dL)
Troponin T hs (µg/L)	________________

LDH

____
(U/L)

Glucose (mg/dl)

____

Ferritine (µg/L)

____

D-dimer (µg/L)

____

Fibrinogen (g/L)

____

eGFR (CKD-EPI) (ml/min)

____

Pathogen Testing

eCRF v. 1.0 - Protocol v. 2.3 - 27-04-2020

PATHOGEN TESTING: SARS-CoV-2- DETECTION
(Enter every sample sent for SARS-CoV-2)

Date of detection	______________________
Sample method	○ NP swap ○ BAL ○ Other
Specify which other method	______________________
Result	○ positive ○ negative
If Yes, specify<br>☐ COVID<br>☐ non-COVID virus<br>☐ bacteria<br>☐ fungus<br>☐ other	
Specify other 1	______________________
Specify other 2 (if applicable)	______________________
Specify other 3 (if applicable)	______________________

Neutralisation antibody titer

Neutralisation antibody titer

Sample taken?	◯ Yes ◯ No
NT50	______________________
NT90	______________________

Adverse Events

eCRF v. 1.0 - Protocol v. 2.3 - 27-04-2020

ADVERSE EVENTS
Definition of 'ADVERSE EVENT' (AE): any untoward medical occurrence in a patient administered a pharmaceutical product and which does not necessarily have a causal relation with this treatment.

Any SERIOUS adverse event (SAE) must be reported within 24 hours of awareness.

Definition of 'SERIOUS adverse event':
A serious adverse event (SAE) is any event that results in any of the following:

Results in death Is life-threatening Requires inpatient hospitalisation or prolongation of existing hospitalisation Results in persistent or significant disability or incapacity Is a congenital anomaly or birth defect Is an important medical event

Legend for severity of AE :

Mild: no or transient symptoms, no interference with the subject's daily activities Moderate: marked symptoms, moderate interference with the subject's daily activities Severe: considerable interference with the subject's daily activities, unacceptable For this trial, only AE graded as severe shall be collected, i.e. adverse events that are life-threatening and/or require an urgent intervention.

Total number of pages ______________________

Describe Event ______________________

MedDRA LLT ______________________
(Most recent version of MedDRA dictionary is uploaded automatically during routine system updates)

Start date ______________________

Stop date ______________________

Outcome	◯ Recovered<br>◯ Recovered with sequelae<br>◯ Not yet recovered<br>◯ Fatal
Action taken	☐ No action taken<br>☐ Medication<br>☐ Non-drug therapy<br>☐ Further investigation performed<br>☐ Stop study participation due to AE<br>☐ Other

Ensure to update Concomitant Therapy page [concomitant_medica_arm_1][form-link:concomitant_therapy: Link to all patient Concomitant Medication (first record)] - Total number of records: [concomitant_medica_arm_1][contx_total][last-instance]

Other	__________________________________
Action taken regarding study treatment	☐ No action taken<br>☐ Temporarily interrupted<br>☐ Stopped permanently<br>☐ Other<br>☐ N/a (study treatment not started yet)
Other	__________________________________

Investigator's assessment

Seriousness

Serious AE?	◯ Yes<br>◯ No
Date of awareness	___________________________

If yes, tick all criteria that apply:

	☐ Resulted in death
Date of death	___________________________
	☐ Is life-threatening
	☐ Results in persistent or significant disability/incapacity
	☐ Requires or prolongs in patient hospitalization
Date of hospitalization	___________________________

Is hospitalization ongoing?	◯ Yes<br>◯ No
Date of discharge	__________________________________
	☐ Is a congenital anomaly or birth defect
	☐ Is considered as an important medical event
Severity	
Severity	◯ Mild<br>◯ Moderate<br>◯ Severe
Causality to study treatment	
Causality	◯ Not Related<br>◯ Unlikely<br>◯ Possibly<br>◯ Probably<br>◯ Definitely<br>◯ Unknown
Expectedness of event	◯ Expected ◯ Unexpected
Causality to study procedure	
Causality to study procedure?	◯ Yes<br>◯ No<br>◯ Unknown<br>(Study procedure = a procedure performed specifically for the study, outside the standard of care)
If yes, specify study-specific procedure	____
Any other comments	____

Concomitant Therapy

eCRF v. 1.0 - Protocol v. 2.3 - 27-04-2020

Total number of pages ______________________

IMPORTANT: Please, list all medication / non-drug therapy taken by the subject while participating in the study

Generic medication name / non-drug therapy ______________________

Reason for concomitant therapy
- ◯ Pre-existing condition
- ◯ Adverse event
- ◯ Other

Define pre-existing condition ___________________________

Adverse event description ______________________

If "Other" reason for concomitant therapy, please specify ___________________________

Start date ______________________

Stop date ______________________

☐ ongoing

Dose ______________________

Dose unit
- ◯ mg
- ◯ gr
- ◯ ml
- ◯ cl
- ◯ IU
- ◯ Appl
- ◯ dr
- ◯ %
- ◯ Other

If "Other", please specify unit: ______________________

End of Study

eCRF v. 1.0 - Protocol v. 2.3 - 27-04-2020

END OF STUDY

Date End of Trial visit ____________________

Primary reason for trial participation termination
- ◯ Subject completed study as per protocol
- ◯ Physician decision
- ◯ Subject decision (withdrew consent)
- ◯ Lost contact with subject
- ◯ Adverse Event
- ◯ Death
- ◯ Protocol violation(s)
- ◯ Other

Date of death ____________________

If "Other" reason for trial participation termination, please specify ____________________

OUTCOME

Outcome
- ◯ Discharged alive
- ◯ Hospitalization
- ◯ Transfer to other facility
- ◯ Death
- ◯ Palliative discharge
- ◯ Unknown

Outcome date ____________________

Date of discharge ____________________

Post-discharged alive
- ☐ Oxygen therapy
- ☐ Dialysis/renal treatment
- ☐ Other intervention/procedure

Specify other intervention/procedure ____________________

Specify transfer facility name ____________________
